# Synthesis, Antifungal Activities and Molecular Docking Studies of Benzoxazole and Benzothiazole Derivatives

**DOI:** 10.3390/molecules23102457

**Published:** 2018-09-25

**Authors:** Bo Luo, Ding Li, An-Ling Zhang, Jin-Ming Gao

**Affiliations:** 1 College of Life Sciences, Xinyang Normal University, Tea Plant Biology Key Laboratory of Henan Province, Xinyang 464000, China; luobo2011@163.com; 2 Shaanxi Key Labotory of Natural Products & Chemical Biology, Shaanxi Engineering Center of Bioresource Chemistry & Sustainable Utilization, College of Chemistry & Pharmacy, Northwest A&F University, Yangling 712100, China; linganzh@163.com

**Keywords:** benzoxazole derivatives, benzothiazole derivatives, fungicidal activity, plant pathogens, structure-activity relationships, molecular docking

## Abstract

Based on benzoxazole and benzothiazole scaffold as an important pharmacophore, two series of 2-(aryloxymethyl) benzoxazole and benzothiazole derivatives were synthesized and their antifungal effects against eight phytopathogenic fungi were evaluated. Compounds **5a**, **5b**, **5h**, and **5i** exhibited significant antifungal activities against most of the pathogens tested. Especially **5a**, **5b**, **5h**, **5i**, **5j**, and **6h** inhibited the growth of *F. solani* with IC_50_ of 4.34–17.61 μg/mL, which were stronger than that of the positive control, hymexazol (IC_50_ of 38.92 μg/mL). **5h** was the most potent inhibitor (IC_50_ of 4.34 μg/mL) against *F. Solani*, which was about nine times more potent than hymexazol. Most of the test compounds displayed significant antifungal effects against *B. cinerea* (IC_50_ of 19.92–77.41 μg/mL), among them, **5a** was the best one (IC_50_ of 19.92 μg/mL). The structure-activity relationships (SARs) were compared and analyzed. The result indicates that the electron-drawing ability and position of the substituents have a significant impact on biological activities. Furthermore, docking studies were carried out on the lipid transfer protein sec14p from *S. cerevisiae*, and preliminarily verified the antifungal activities. Taken together, these results provide 2-(phenoxymethyl)benzo[d]oxazole as an encouraging framework that could lead to the development of potent novel antifungal agents.

## 1. Introduction

Plant diseases lead to serious losses to agriculture worldwide and pose an emerging threat to the global food security [1]. Especially, phytopathogenic fungi, which are extremely difficult to manage in agricultural production, have caused the most serious problems, accounting for 70–80% of plant diseases. Several important fungal plant pathogens, such as the genera *Colletotrichum*, *Botrytis*, *Alternaria*, and *Fusarium* have attracted great attention due to their typical infected disease symptoms (e.g., anthracnose caused by *Colletotrichum* species characterized by sunken necrotic lesions usually surrounded by a red margin) [2]. Chemical treatments have shown promising prospect in the control of the disease [3]. However, the chemicals have also led to fungicide resistance and environmental pollution, which may pose a serious risk to animal and human health [4]. Therefore, there is an increasing need for more safe and environment friendly antifungal agents to effectively control these plant diseases. 

**Figure 1:**
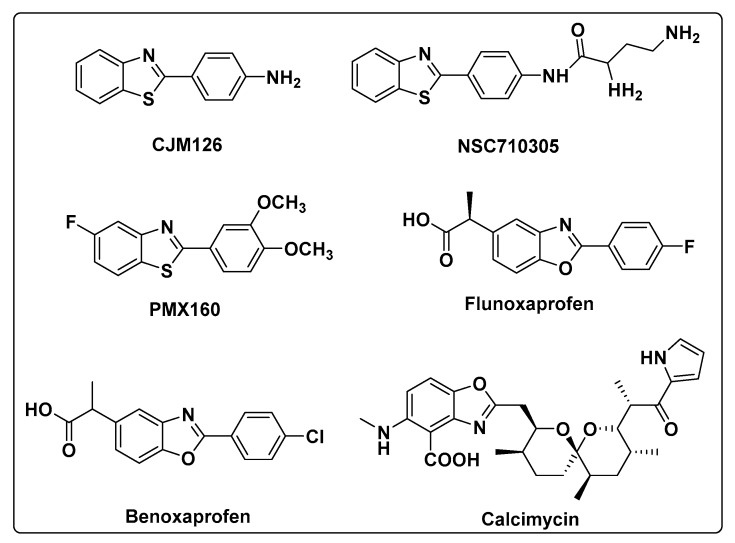
Chemical structures of some drugs bearing benzoxazole and benzothiazole scaffolds.

The benzoxazole and benzothiazole scaffolds are found in various synthetic drugs displaying a broad-spectrum biological activities including antiparasitic, antibacterial, antiviral, cytotoxic, and herbicidal properties [5,6,7]. Thus they usually act as the important guide frame and parent skeleton, playing very important roles in medicinal chemistry and agrochemicals. For example, they can emerge as medicament because of their anti-inflammatory, antimalarial, and antitumor properties [8,9,10]. What is more, they often have such advantage as good selectivity, high activity and are eco-friendly compared with pesticides. Chemical structures of some drugs bearing benzoxazole and benzothiazole scaffolds are showed in Figure 1. 

**Figure 2:**
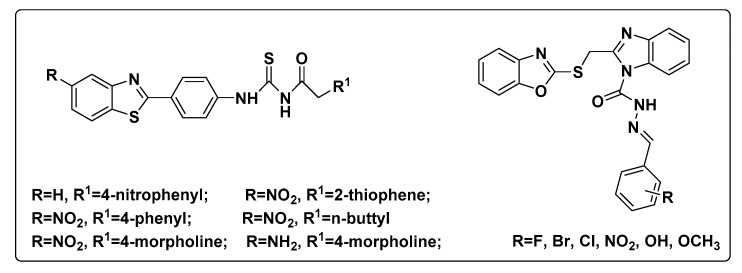
Chemical structures of the antifungal compounds bearing benzoxazole and benzothiazole scaffolds.

However, to the best of our knowledge, few reports [11,12] on fungicidal activity of benzoxazole and benzothiazole derivatives against plant pathogenic fungi have been published. The chemical structures of these antifungal compounds were showed in Figure 2. Thus, it is of great significance to design and synthesize antifungal compounds bearing benzoxazole and benzothiazole scaffolds. 

**Scheme 1:**
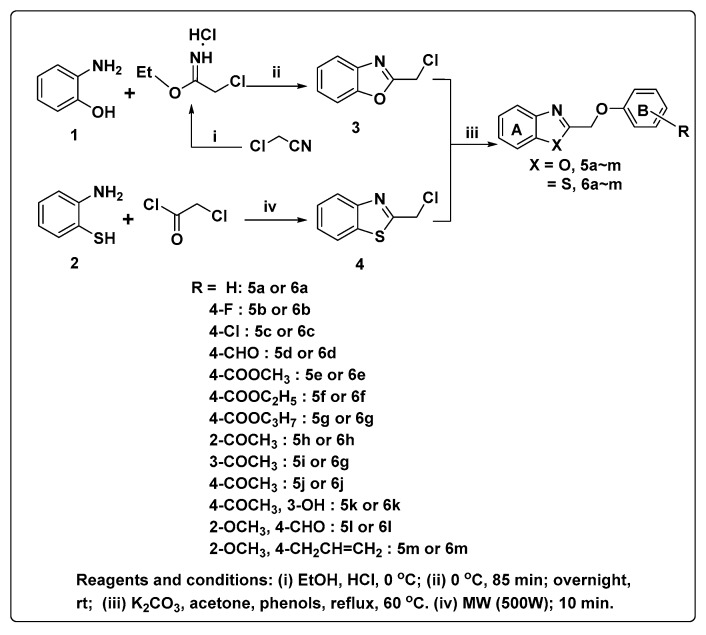
Synthetic routes for 2-(Aryloxymethyl)-benzoxazole and -benzothiazole derivatives.

In our previous studies, benzimidazole derivatives and alkyl or benzyl acetophenone ether derivatives were synthesized, and some compounds showed promising fungicidal activities to plant pathogens [13,14]. In continuation of our investigation on antifungal compounds, two series of benzoxazole and benzothiazole derivatives were designed and synthesized from intermediates 2-chloromethyl-benzoxazole and 2-chloromethyl-benzothiazole through etherification reaction (Scheme 1), their structures were characterized by ^1^H and ^13^C-NMR spectroscopy and mass spectroscopy (MS). Their fungicidal activities against eight agricultural phytopathogenic fungi were evaluated by the mycelium growth rate method, and the structure-activity relationships of these derivatives were also discussed. 

## 2. Results and Discussion

### 2.1. Chemistry

The synthetic pathways for the preparation of the target products **5a**–**m** and **6a**–**m** are shown in Scheme 1. In general, intermediate 2-chloromethyl-benzoxazole **3** can be commonly obtained by condensation of 2-aminophenol with chloroacetyl chloride [15,16] or chloroacetic acid [17]. We tried to adapt the methods reported, but the yield was unsatisfactory. In addition, both methods used polyphosphoric acid (PPA) as a reactant, which not only caused difficulty in aftertreatment but was also unfriendly to the environment [18,19]. In order to overcome these disadvantages, we reacted 2-aminophenol with ethyl chloroacetimidate hydrochloride in methylene chloride at 0 °C to provide the compound 3 with 86.5% yield according to the method described by Sheng [20]. 

Similarly, 2-chloromethyl-benzothiazole **4** can also be obtained from the condensation of 2-aminothiophenols with chloroacetyl chloride [21,22,23], chloroacetic acid [24,25,26], or chloroacetonitrile [9]. Firstly, we tried to prepare **4** according to these literatures, but the yield was not ideal. Then we employed a microwave-assisted procedure developed by Gellis [27], treating 2-aminobenzenethiol **2** with 2-chloroacetyl chloride in acetic acid under microwave irradiation for 10 min to provide the required intermediate **4**, to our delight, the yield was up to 87%. Compared with the traditional method, the microwave-assisted procedure was environmentally friendly and efficient, with a less time and higher yield. Furthermore, we also tried to prepare **3** by exploiting the microwave-assisted method, but it did not work. At last, Williamson reaction of **3** or **4** with different substituted phenols in dry acetone in the presence of anhydrous K_2_CO_3_ at reflux gave the desired compounds **5a**–**m** or **6a**–**m**.

The structures of the synthetic compounds and key intermediates were confirmed by ^1^H-NMR, ^13^C-NMR and MS, as well as HRMS (High Resolution Mass Spectrum). Out of the 26 synthesized derivatives, 10 compounds (**5d**, **5f**–**i**, **5k**, **5m**, **6g**, **6k**, and **6m**) were novel. 

### 2.2. Antifungal Activity and Structure-Activity Relationships

All the 2-(aryloxymethyl) benzoxazole and benzothiazole derivatives (**5a**–**m, 6a**–**m**) obtained were screened for their preliminary antifungal activity in vitro. Their activities were evaluated on eight different phytopathogenic fungi: *Fusarium oxysporum f.* sp. *niveum*, *Fusarium. graminearum*, *Fusarium. solani*, *Alternaria solani*, *Colletotrichum gloeosporioides*, *Valsa mali*, *Magnaporthe oryzae*, and *Botrytis cinerea* (Table 1).

The initial results showed in Table 1 indicated that some of these synthetic compounds presented significant antifungal effects. These results encouraged us to continue our investigations into further evaluation of their antifungal effects according to the inhibition rates over 60% that were determined for IC_50_ values (Table 2). 

Compounds (**5a**, **5b**, **5c**, **5f**, **5g**, **5h**, **5i**, **5j**, **5m**, **6a**, **6c**, **6h,** and **6l**) displayed varying degrees of antifungal activities against several plant pathogenic fungi tested, while the other compounds showed lower activities, compared with the positive control hymexazol.

#### 2.2.1. Antifungal Activity of the Benzoxazole Derivatives

As shown in Table 2, of the test benzoxazole derivatives, compound **5a** with a non-substituted phenyl ring B exhibited a broad spectrum of biological activity on all of the eight species of plant pathogens (IC_50_ of 12.27–65.25 μg/mL). **5b** with a fluorine group had good antifungal activities against *F. Solani*, *A. solani*, *C. gloeosporioides*, *M. oryzae*, and *B. cinerea*, showing IC_50_ values in the range of 15.98 to 32.78 μg/mL. However, **5c** only effected to *B. Cinerea* (IC_50_ of 50.04 μg/mL). Notably, the order of antifungal activity of **5a**, **5b**, and **5c** against most of the eight plant pathogens was **5a** > **5b** > **5c**. The results suggested that the introduction of a chlorine or a fluorine atom into the phenyl ring of **5a** decreased antifungal activity and the introduction of F into **5a** produced higher broad-spectrum antifungal properties than that of Cl. In general, introduction of halogens such as chlorine or fluorine atom into pesticides can enhance activity. But our results were diametric. We supposed one reason was that introducing the electron-withdrawing group on the benzene ring will change the extension direction of the benzene ring, thus weakening the van der Waals interactions with residues Ser201, Gln202, Try205, Pro205, Arg208, and Met209.

Inhibitory activity of **5f** and **5g**, possessing an aliphatic formate group (e.g., –CO_2_C_2_H_5_), against *B. cinerea* was almost similar, with IC_50_ values of 73.04 and 77.41 μg/mL, respectively, but were inactive against the other fungi tested, indicating the aliphatic formate on the *p*-position of B-ring of benzoxazole derivatives is detrimental to activity.

In addition, the order of antifungal activity of **5h**, **5i**, and **5j**, which all carried a COMe group, against *F. solani* was **5h** > **5j** > **5i**, with their IC_50_ data of 4.34, 16.53 and 17.61 μg/mL, respectively, which were all better than the control (IC_50_ of 38.92 μg/mL), indicating **5h** to be the most active compound. In comparison to the other pathogenic organisms tested, **5h** and **5i** produced more broad-spectrum antifungal properties than that of **5j**. These observations suggested that the acetyl group on *o*-position and *m*-position of acetophenones of benzoxazoles is better than *p*-position in antifungal activity. 

#### 2.2.2. Antifungal Activity of the Benzothiazole Derivatives

Most of the test benzothiazole derivatives exerted weak antifungal activity (Table 1 and Table 2). However, among the test compounds **6a**, **6c**, **6h**, and **6l**, compound **6h**, containing an electron-withdrawing acetyl group on the 2-position of the phenyl ring, significantly inhibited the mycelial growth of of *F. graminearum*, *F. Solani* and *C. gloeosporioides*, with IC_50_ values of 23.39, 15.55 and 29.61 μg/mL, respectively, but were essentially inactive against the other five fungi (Table 2). Additionally, **6a**, **6c**, and **6l** elicited selective and moderate inhibitory effects against *B. cinerea*, with IC_50_ values of 62.62, 61.20, and 50.42 μg/mL, respectively, while being almost inactive to the other organisms tested. 

On the other hand, most of the test compounds exhibited moderate to strong inhibitory effects on *F. solani* (IC_50_ of 4.34–17.61 μg/mL) and *B. cinerea* (IC_50_ of 19.92–77.41 μg/mL), implying that they are more sensitive to *F. solani* and *B. cinerea* than the other six organisms. Interestingly, between the two species of the genus *Fusarium* tested, *F. solani* is more sensitive.

**Figure 3:**
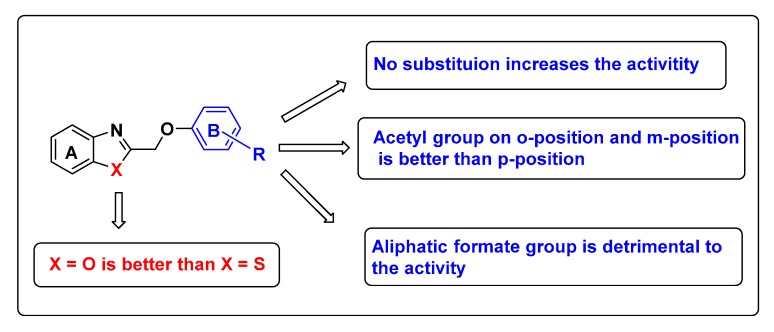
Structure activity relationship of the synthesized compounds.

Comparing the antifungal activity of benzoxazole derivatives with benzothiazole derivatives, antifungal effects of most of benzoxazole derivatives were better than those of benzothiazoles, showing that the benzoxazole derivatives may have a more promising future as fungicides. For example, Compound **5a** was three-fold more active than the corresponding **6a** in antifungal potency against *B. cinerea* (IC_50_ of 19.92 and 62.62 μg/mL respectively). The structure activity relationship of the synthesized compounds is shown in Figure 3.

### 2.3. Molecular Docking Studies

Molecular docking is a fast and efficient computational method to predict the bioactive compounds to a specific protein or reversely predict the target proteins for one bioactive compound [28]. The significant experimental antifungal activities of compounds **5a**, **5b**, **5h**, **5i**, **5j**, and **6h** provide a hint to performing molecular docking studies to identify the potential target. On the one hand, reverse docking was applied to predict the probable targets of the title compounds from antifungal drug proteins, such as sterol 14-alpha demethylase, squalene epoxidase, chitin synthase, fattyacid synthase, *N*-myristoyltransferase, and tubulin, etc. On the other hand, after reviewing the different chemical classes that docked into the Sec14p active site, such as nitrophenyl(4-(2-methoxyphenyl)piperazin-1-yl)methanones (NPPMs), picolinamide, and benzamide chemotypes, we examined our compounds on the active site because some structural similarities exist. The results showed that lipid transfer protein Sec14p might be a potential target for our compounds. Sec14-like phosphatidylinositol transfer proteins (PITPs) integrate diverse territories of intracellular lipid metabolism with stimulated phosphatidylinositol-4-phosphate production and are discriminating portals for interrogating phosphoinositide signaling [29]. Sec14p, the major PITP, executes essential functions in several pathogenic fungi and it is required for the efficient secretion of pathogenicity factors. It is an attractive antifungal target as it has been shown to be druggable [30,31].

**Figure 4:**
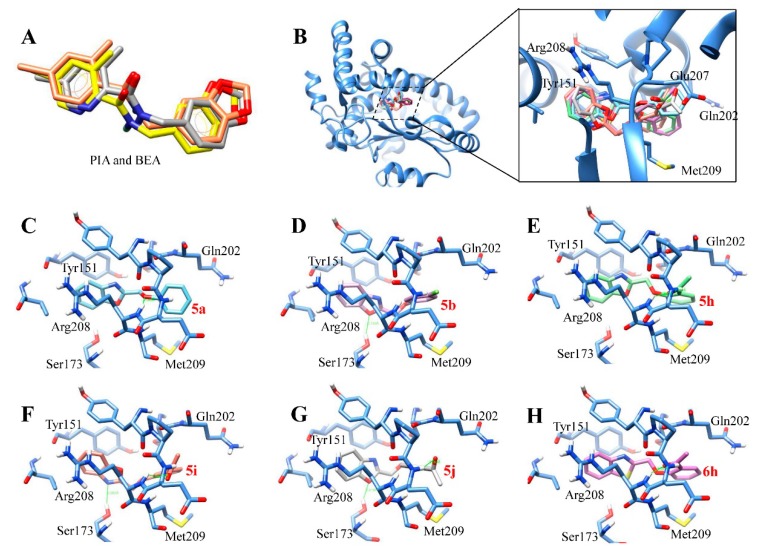
The binding models of the test compounds into the lipid binding pocket of Sec14p from *S. cerevisiae.* (**A**) Superposition of PIA (yellow) and BEA (orange) in docking resultant models with PIA in the X-ray crystallographic structure (PDB 6F0E; gray). (**B**) Overlay of the six test benzoxazole and benzothiazole derivatives. (C–H) The binding modes of the compounds (in stick model with carbon) into the active site of Sec14p: (**C**) **5a** (aquamarine); (**D**) **5b** (orchid); (**E) 5h** (light green); (**F**) **5i** (coral); (**G**) **5j** (light gray); and (**H**) **6h** (hot pink).

The picolinamide (PIA) and the benzamide (BEA), reported by professor Hoepfner, are two antifungal compounds with IC_50_ values of 28.5 and 6.6 μM against *S. cerevisiae*, respectively. To test this hypothesis, PIA and BEA, as the reference inhibitors, were also docked into the lipid binding pocket of Sec14p. In the resulting docking model, the binding pose of PIA was almost identical to the crystallographic one (PDB 6F0E) (Figure 4A). The binding energy of PIA and BEA were −6.96 and −7.54 kcal/mol, and the inhibit constants were predicted to be 7.97 and 2.99 μM, respectively, which were similar to the experimental in vitro data (Table 3). All these results showed the docking protocols were appropriate and feasible. As shown in Figure 4, the six-test compound shared very similar binding modes with comparable binding affinities (Table 3). The benzoxazole and benzothiazole ring pointed toward the core of the cavity and engaged in a π–π stacking interaction with Tyr151. The phenoxyl ether moieties were oriented toward solvent and made van der Waals interactions with residues Ser201, Gln202, Try205, Pro205, Arg208, and Met209. A hydrogen-bond (H-bond) interaction between the carboxyl of Ser173 and the heteroatom in the benzoxazole ring of the compound **5b** (or **5i**/**5j**) could be observed. The binding models of **5h**, **5i**, and **5j** outlines that the acetyl group on the *p*-position of acetophenones of benzoxazoles destabilized the π–π stacking interaction between the benzoxazole ring with Tyr151 and would be deleterious for ligand binding. Additionally, the number of binding energy (or inhibit constant) for benzoxazole and benzothiazole derivatives with Sec14p, and that for PIA and BEA, it was in the same order of magnitude, which may be one reason for these compounds with different molecular skeleton having similar fungicidal activity. 

## 3. Materials and Methods

### 3.1. Chemistry

All of the commercial solvents and reagents were of reagent grade. The melting points of test compounds were measured on an X-4 apparatus (Beijing Tech Instrument Co., Beijing, China) and uncorrected. ^1^H-NMR and ^13^C-NMR spectra were recorded on a Bruker Avance III 500 MHz spectrometer (Bruker Bios pin, Rheinstetten, Germany) with in CDCl_3_ with tetramethylsilane (TMS) as internal reference. High-resolution electrospray ionization mass spectrometry (HRESIMS) data were recorded on LCMS-IT-TOF (Shimadzu, Kyoto, Japan). Thin-layer chromatography (TLC) was performed on silica gel 60 F_254_ (Qingdao Marine Chemical Ltd., Qingdao, China). Column chromatography (CC) was performed over silica gel (200−300 mesh, Qingdao Marine Chemical Ltd., Qingdao, China). 

#### 3.1.1. General Experimental Procedures

Synthesis of ethyl chloroacetimidate hydrochloride. A solution of chloroacetonitrile (10.0 g, 133 mmol), ethanol (6.8 g, 148 mmol), and dry ether (76 mL) was stirred and cooled at 0 °C while a stream of dry HCl gas was bubbled through. After 0.5 h, the white crystalline salt had precipitated. Excess dry ether was added to the mixture, which was then filtered, washed with dry ether, and stored over P_2_O_5_ in vacuo, leading to ethyl chloroacetimidate hydrochloride as a white solid (16 g, yield 77.6%) [14]. m.p., 89–91 °C. 

Synthesis of 2-Chloromethyl-benzoxazole (**3**) [20]. To a solution of 2-aminophenol (3.76 g, 34.5 mmol) in methylene chloride at 0 °C was added ethyl chloroacetimidate hydrochloride (7.98 g, 50.5 mmol). After 85 min, the reaction mixture was allowed to warm to room temperature, while being stirred overnight. The mixture was filtered over diatomite and concentrated to oil under reduced pressure. The resulting residue was purified by column chromatography on silica gel (petroleum ether/acetone, 10:1 *v*:*v*) to obtain compound **3** as a white solid (5.00 g, 86.5%). m.p., 152–154 °C; MS (+ESI) *m*/*z* 168.05 [M + H]^+^.

Synthesis of 2-Chloromethyl-benzothiazole (**4**) [27]. To a solution of 2-aminobenzenethiol (1 g, 7.93 mmol) in acetic acid (15 mL), and 2-chloroacetyl chloride (1.35 g, 1.19 mmol) was added dropwise. The reaction mixture was irradiated in a microwave oven (Ethos start) for 10 min at a power of 500 W. After cooling, the mixture was poured onto crushed ice (100 mL) and basified with 5 mol/L NaOH. The solution was extracted with chloroform (3 × 50 mL). The combined organic layers were dried over MgSO_4_ and concentrated under vacuum. Purification of the residue by column chromatography on silica gel (petroleum ether/acetone, 10:1 *v*:*v*) gave compound **4** as a yellow solid (1.90 g, 86.8%) [27]. m.p., 89–90 °C; MS (+ESI) *m*/*z* 184.02 [M + H]^+^.

#### 3.1.2. General Procedure for the Synthesis of the Benzoxazole and Benzothiazole Derivatives (**5a**–**m**, **6a**–**m**)

To a solution of various substituted phenols (1 mmol) in dry acetone (30 mL) K_2_CO_3_ (1 mmol) and compound **3** or **4** (1 mmol) were added. After being stirred for 4 h at reflux temperature, the reaction mixture was cooled, filtered, and concentrated under vacuum. Then the residue was diluted with 30 mL ethyl acetate and sequentially washed with 30 mL 1 M HCl, aq. NaHCO_3_ solution and brine in order. The organic layer was dried over MgSO_4_ and concentrated in vacuo. Purification of the residue by chromatography on silica gel furnished target compounds. ^1^H-NMR, ^13^C-NMR and mass spectroscopy (MS) of compounds **5a**–**m** and **6a**–**m** are shown in Supplementary Materials.

*2-(phenoxymethyl)benzo[d]oxazole* (**5a**): Yellow solid; yield, 75.56%; m.p., 154–156 °C; ^1^H-NMR (500 MHz, CDCl_3_) *δ* 7.76 (m, 1H, ArH), 7.56 (d, *J* = 6.9 Hz, 1H, ArH), 7.37 (m, 2H, ArH × 2), 7.32 (t, *J* = 7.7 Hz, 2H, ArH × 2), 7.07 (d, *J* = 8.4 Hz, 2H, ArH × 2), 7.02 (t, *J* = 7.3 Hz, 1H, ArH), 5.33 (s, 2H, CH_2_); and ESI-MS *m*/*z*: 225.94 ([M + H]^+^), 247.92 ([M + Na]^+^).

*2-((4-fluorophenoxy)methyl)benzo[d]oxazole* (**5b**): Yellow solid; yield, 83.13%; m.p., 62–63 °C; ^1^H-NMR (500 MHz, CDCl3) δ 7.76 (dd, *J* = 5.9, 2.5 Hz, 1H, ArH), 7.55 (m, 1H, ArH), 7.36 (dd, *J* = 5.7, 2.3 Hz, 2H, ArH × 2), 7.13–6.92 (m, 4H, ArH × 4), 5.28 (s, 2H, –CH2–O–); and ESI-MS *m*/*z*: 244.07 ([M + H]^+^).

*2-((4-chlorophenoxy)methyl)benzo[d]oxazole* (**5c**): Yellow solid; yield, 82.31%; m.p., 85–86 °C; ^1^H-NMR (500 MHz, CDCl3) δ 7. 74 (m, 1H, ArH), 7.53 (dd, *J* = 7.3, 1.1 Hz, 1H, ArH), 7.35 (m, 2H, ArH × 2), 7.22 (d, *J* = 9.0 Hz, 2H, ArH × 2), 6.96 (d, *J* = 8.9 Hz, 2H, ArH × 2), 5.27 (s, 2H, –CH2–O–); and ESI-MS *m*/*z*: 259.84 ([M + H]^+^), 281.85 ([M + Na]^+^). 

*4-(benzo[d]oxazol-2-ylmethoxy)benzaldehyde* (**5d**): White solid; yield, 34%; m.p., 138–140 °C; ^1^HNMR (500 MHz, CDCl3) δ 9.89 (*d*, *J* = 12.2 Hz, 1H, –CHO), 7.86 (d*d*, *J* = 9.4, 2.5 Hz, 2H, ArH × 2), 7.77 (m, 1H, ArH), 7.56 (d, *J* = 7.3 Hz, 1H, ArH), 7.38 (m, 2H, ArH × 2), 7.18 (d, *J* = 8.7 Hz, 2H, ArH × 2), 5.41 (d, *J* = 7.2 Hz, 2H, CH2); 13C NMR (125 MHz, CDCl3) δ 190.9 (C=O), 162.8 (–N=C–O–), 160.7 (ArC), 151.2 (ArC), 140.7 (ArC), 132.3 (ArC), 131.2 (ArC), 126.2 (ArC), 125.1 (ArC), 120.7 (ArC), 115.4 (ArC), 111.2 (ArC), 63.0 (–CH2O–); HR-MS (ESI): *m*/*z* calcd. for C15H11NO3: 254.0812; found: 254.0785 [M + H]^+^.

*methyl 4-(benzo[d]oxazol-2-ylmethoxy)benzoate* (**5e**): White solid; yield, 67.14%; m.p., 97–99 °C; ^1^H-NMR (500 MHz, CDCl3) δ 8.01 (dd, *J* = 11.5, 4.5 Hz, 2H, ArH × 2), 7.77 (m, 1H, ArH), 7.56 (d, *J* = 7.2 Hz, 1H, ArH), 7.37 (m, 2H, ArH × 2), 7.08 (dd, *J* = 11.5, 4.5 Hz, 2H, ArH × 2), 5.37 (d, *J* = 9.1 Hz, 2H, CH2), 3.87 (d, *J* = 8.8 Hz, 3H, CH3); ESI-MS *m*/*z*: 284.37 ([M + H]+), 306.81 ([M + Na]^+^).

*ethyl 4-(benzo[d]oxazol-2-ylmethoxy)benzoate* (**5f**): Yellow solid; yield, 67.34%; m.p., 70–72 °C; ^1^H-NMR (500 MHz, CDCl3) δ 8.02 (d, *J* = 8.7 Hz, 2H, ArH × 2), 7.77 (m, 1H, ArH), 7.55 (m, 1H, ArH), 7.37 (dd, *J* = 7.4, 3.6 Hz, 2H, ArH × 2), 7.08 (d, *J* = 8.7 Hz, 2H, ArH × 2), 5.38 (s, 2H, –CH2–O–), 4.34 (d, *J* = 7.1Hz, 2H, –CH2–CH3), 1.37 (t, *J* = 7.1 Hz, 3H, CH3); ^13^C-NMR (125MHz, CDCl3) δ 166.4 (C=O), 161.6 (–N=C–O–), 161.0 (ArC), 151.2 (ArC), 140.7 (ArC), 131.9 (ArC), 126.1 (ArC), 125.1 (ArC), 124.5 (ArC), 120.7 (ArC), 114.6 (ArC), 111.2 (ArC), 63.0 (–CH2–O–), 61.0(–CH2–CH3), 14.6 (CH3); HR-MS (ESI): *m*/*z* calcd. for C17H15NO4: 298.1074; and found: 298.1043 [M + H]^+^.

*propyl 4-(benzo[d]oxazol-2-ylmethoxy)benzoate* (**5g**): White solid; yield, 70.42%; m.p., 44–45 °C; ^1^H-NMR (500 MHz, CDCl3) δ 8.00 (d, *J* = 8.6 Hz, 2H, ArH × 2), 7.73 (d, *J* = 6.6 Hz, 1H, ArH), 7.52 (d, *J* = 7.3 Hz, 1H, ArH), 7.33 (m, 1H, ArH), 7.06 (d, *J* = 8.6 Hz, 2H, ArH × 2), 5.35 (s, 2H, –CH2–O–), 4.22 (t, *J* = 6.6 Hz, 2H, –CH2–CH2–CH3), 1.74 (dd, *J* = 14.1, 7.1 Hz, 2H, –CH2–CH2–CH3), 0.99 (t, *J* = 7.4 Hz, 3H, CH3); ^13^C-NMR (125 MHz, CDCl3) δ 166.3 (C=O), 161.5 (–N=C–O–), 160.9 (ArC), 151.1 (ArC), 140.8 (ArC), 131.8 (ArC), 131.7 (ArC), 125.9 (ArC), 124.9 (ArC), 124.8 (ArC), 124.4 (ArC), 120.6 (ArC), 114.5 (ArC), 111.1 (ArC), 66.5 (–CH2–O–), 62.9 (–CH2–CH2–CH3), 22.3 (–CH2–CH2–CH3), 10.6 (CH3); HR-MS (ESI): *m*/*z* calcd. for C18H17NO4: 312.1230; and found: 312.1204 [M + H]^+^.

*1-(2-(benzo[d]oxazol-2-ylmethoxy)phenyl)ethanone* (**5h**): Red solid; yield, 65.17%; m.p., 98–100 °C; ^1^H-NMR (500 MHz, CDCl3) δ 7.76 (m, 2H, ArH × 2), 7.57 (m, 1H, ArH), 7.47 (t, *J* = 7.8 Hz, 1H, ArH), 7.38 (m, 2H, ArH × 2), 7.14 (d, *J* = 8.3 Hz, 1H, ArH), 7.07 (t, *J* = 7.5 Hz, 1H, ArH), 5.44 (s, 2H, CH2), 2.68 (s, 3H, –COCH3); 13C NMR (125 MHz, CDCl3) δ 199.7 (C=O), 161.0 (–N=C–O–), 157.2 (ArC), 151.2 (ArC), 140.7 (ArC), 133.9 (ArC), 130.9 (ArC), 129.3 (ArC), 126.2 (ArC), 125.1 (ArC), 122.2 (ArC), 120.7 (ArC), 113.1 (ArC), 111.2 (ArC), 63.4 (CH2), 32.2 (CH3); HR-MS (ESI): *m*/*z* calcd. for C16H13NO3: 268.0968; and found: 268.0940 [M + H]^+^.

*1-(3-(benzo[d]oxazol-2-ylmethoxy)phenyl)ethanone* (**5i**): Yellow solid; yield, 82.4%; m.p., 71–72 °C; ^1^H-NMR (500 MHz, CDCl3) δ 7.74 (m, 1H, ArH), 7.64 (s, 1H, ArH), 7.56 (d, *J* = 7.6 Hz, 1H, ArH), 7.52 (dd, *J* = 5.4, 2.9 Hz, 1H, ArH), 7.34 (m, 3H, ArH × 3), 7.24 (s, 1H, ArH), 5.34 (s, 2H, CH2), 2.55 (s, 3H, –COCH3); ^13^C-NMR (125 MHz, CDCl3) δ 197.3 (C=O), 160.9 (–N=C–O–), 158.0 (ArC), 150.8 (ArC), 140.7 (ArC), 138.6 (ArC), 129.8 (ArC), 125.6 (ArC), 124.6 (ArC), 122.0 (ArC), 120.4 (ArC), 119.9 (ArC), 113.8 (ArC), 110.8 (ArC), 62.8 (CH2), 26.6 (CH3); HR-MS (ESI): *m*/*z* calcd. for C16H13NO3: 268.0968; and found: 268.0935 [M + H]^+^.

*1-(4-(benzo[d]oxazol-2-ylmethoxy)phenyl)ethanone* (**5j**): Yellow solid; yield, 49.44%; m.p., 110–112 °C; ^1^H-NMR (500 MHz, CDCl3) δ 7.90 (d, *J* = 8.7 Hz, 2H, ArH × 2), 7.72 (m, 1H, ArH), 7.50 (d, *J* = 6.5 Hz, 1H, ArH), 7.33(m, 2H, ArH × 2), 7.06 (d, *J* = 8.7 Hz, 2H, ArH × 2), 5.35 (s, 2H, CH2), 2.50 (s, 3H, –COCH3); ^13^C-NMR (125 MHz, CDCl3) δ 196.7 (C=O), 161.6 (–N=C–O–), 160.8 (ArC), 151.0 (ArC), 140.7 (ArC), 131.5 (ArC), 130.8 (ArC), 125.9 (ArC), 124.9 (ArC), 120.6 (ArC), 114.6 (ArC), 111.1 (ArC), 62.8 (CH2), 26.4 (CH3); HR-MS (ESI): *m*/*z* calcd. for C16H13NO3: 268.0968; and found: 268.0942 [M + H]^+^

*1-(4-(benzo[d]oxazol-2-ylmethoxy)-2-hydroxyphenyl)ethanone* (**5k**): White solid; yield, 31.8%; m.p., 98–99 °C; ^1^H-NMR (500 MHz, CDCl3) δ 7.75 (d, *J* = 6.3 Hz, 1H, ArH), 7.64 (d, *J* = 8.7 Hz, 1H, ArH), 7.54 (m, 1H, ArH), 7.36(m, 2H, ArH × 2), 6.58 (d, *J* = 10.9 Hz, 2H, ArH × 2), 5.33(s, 2H, CH2), 2.53 (s, 3H, –COCH3); ^13^C-NMR (125 MHz, CDCl3) δ 203.0 (C=O), 165.2 (–N=C–O–), 164.1 (ArC), 160.6 (ArC), 151.1 (ArC), 140.7 (ArC), 132.8 (ArC), 126.1 (ArC), 125.0 (ArC), 120.7 (ArC), 115.0 (ArC), 111.2 (ArC), 107.8 (ArC), 102.3 (ArC), 62.9 (CH2), 26.4 (CH3); HR-MS (ESI): *m*/*z* calcd. for C16H13NO4: 284.0917; and found: 284.0888 [M + H]^+^.

*4-(benzo[d]oxazol-2-ylmethoxy)-3-methoxybenzaldehyde* (**5l**): Yellow solid; yield, 66.43%; m.p., 100–102 °C; ^1^H-NMR (500 MHz, CDCl3) δ 9.82 (s, 1H, –CHO), 7.72 (d, *J* = 6.5 Hz, 1H, ArH), 7.51 (d, *J* = 7.1 Hz, 1H, ArH), 7.39 (d, *J* = 12.1 Hz, 2H, ArH × 2), 7.33 (dd, *J* = 5.9, 2.7 Hz, 2H, ArH × 2), 7.17 (d, *J* = 8.1 Hz, 1H, ArH), 5.44 (s, 2H, CH2), 3.90 (s, 3H, CH3); 13C NMR (125 MHz, CDCl3) δ 190.9 (C=O), 160.6 (–N=C–O–), 152.6 (ArC), 151.1 (ArC), 150.3 (ArC), 140.7 (ArC), 131.5 (ArC), 126.4 (ArC), 126.0 (ArC), 124.9 (ArC), 120.6 (ArC), 113.2 (ArC), 111.2 (ArC), 109.9 (ArC), 63.8 (CH2), 56.2 (CH3); and ESI-MS *m*/*z*: 284.73 ([M + H]^+^), 307.03 ([M + Na]^+^)

*2-((4-allyl-2-methoxyphenoxy)methyl)benzo[d]thiazole* (**5m**): Brown liquid; yield, 68%; ^1^H-NMR (500 MHz, CDCl3) δ 7.65 (dd, *J* = 94.3, 7.1 Hz, 1H, ArH), 7.35 (m, 1H, ArH), 6.92 (dd, *J* = 73.5, 8.3 Hz, 1H, ArH), 6.71 (d, *J* = 28.9 Hz, 2H, ArH × 2), 5.94 (dd, *J* = 16.2, 10.1, 6.0 Hz, 1H, –CH=CH2), 5.35 (s, 2H, –CH2–O–), 5.07 (d, *J* = 17.0 Hz, 2H, –CH=CH2), 3.87 (d, *J* = 2.9 Hz, 3H, CH3), 3.33(d, *J* = 5.4 Hz, 2H, –CH2CH=CH2); 13C NMR (125MHz, CDCl3) δ 162.0 (–N=C–O–), 151.3 (ArC), 150.1 (ArC), 145.8 (ArC), 141.0 (ArC), 138.1 (ArC), 137.6 (ArC), 135.2 (–CH2CH=CH2), 125.7 (ArC), 124.8 (ArC), 120.6 (ArC), 116.1 (ArC), 115.8 (ArC), 112.7 (–CH2CH=CH2), 111.2 (ArC), 64.7 (–CH2–O–), 56.1 (CH3), 40.1 (–CH2CH=CH2); HR-MS (ESI): *m*/*z* calcd. for C18H17NO3: 296.1281; and found: 296.1256 [M + H]^+^.

*2-(phenoxymethyl)benzo[d]thiazole* (**6a**): White solid; yield, 56.02%; m.p., 74–76 °C; ^1^H-NMR (500 MHz, CDCl3) δ 8.04 (d, *J* = 8.1 Hz, 1H, ArH), 7.90 (d, *J* = 8.0 Hz, 1H, ArH), 7.50 (t, *J* = 7.7 Hz, 1H, ArH), 7.40 (t, *J* = 7.6 Hz, 1H, ArH), 7.32 (t, *J* = 7.7 Hz, 2H, ArH × 2), 7.05 (d, *J* = 8.5 Hz, 2H, ArH × 2), 7.02 (m, 1H, ArH), 5.50 (s, 2H, CH2); and ESI-MS *m*/*z*: 241.89 ([M + H]^+^), 263.87 ([M + Na]^+^).

*2-((4-fluorophenoxy)methyl)benzo[d]thiazole* (**6b**): Yellow solid; yield, 66.41%; m.p., 86–88 °C; ^1^H-NMR (500 MHz, CDCl3) δ 8.05 (d, *J* = 8.2 Hz, 1H, ArH), 7.90 (d, *J* = 8.0 Hz, 1H, ArH), 7.50 (dd, *J* = 11.3, 4.1 Hz, 1H, ArH), 7.41 (dd, *J* = 11.2, 4.0 Hz, 1H, ArH), 6.98 (d, *J* = 6.4 Hz, 4H, ArH × 4), 5.46 (s, 2H, -CH2-O-); and ESI-MS *m*/*z*: 260.11 ([M + H]+), 282.14 ([M + Na]^+^).

*2-((4-chlorophenoxy)methyl)benzo[d]thiazole* (**6c**): Yellow solid; yield, 79.71%; m.p., 83–86 °C; ^1^H-NMR (500 MHz, CDCl3) δ 8.04 (d, *J* = 8.2 Hz, 1H, ArH), 7.88 (d, *J* = 7.9 Hz, 1H, ArH), 7.50 (dd, *J* = 11.3, 4.1 Hz, 1H, ArH), 7.41 (t, *J* = 7.6 Hz, 1H, ArH), 7.23 (d, *J* = 9.0 Hz, 2H, ArH × 2), 6.93 (d, *J* = 8.9 Hz, 2H, ArH × 2), 5.43 (s, 2H, –CH2–O–); and ESI-MS *m*/*z*: 275.81 ([M]^+^).

*4-(benzo[d]thiazol-2-ylmethoxy)benzaldehyde* (**6d**): Yellow solid; yield, 50.19%; m.p., 106–107 °C; ^1^H-NMR (500 MHz, CDCl3) δ 9.90 (s, 1H, –CHO), 8.07 (d, *J* = 8.1 Hz, 1H, ArH), 7.92 (d, *J* = 8.0 Hz, 1H, ArH), 7.87 (d, *J* = 8.6 Hz, 2H, ArH × 2), 7.53 (t, *J* = 7.3 Hz, 1H, ArH), 7.45 (t, *J* = 7.6 Hz, 1H, ArH), 7.16 (d, *J* = 8.5 Hz, 2H, ArH × 2), 5.60 (s, 2H, CH2); 13C NMR (125MHz, CDCl3) δ 191.1 (C=O), 167.7 (–N=C–S–), 162.8 (ArC), 152.4 (ArC), 135.0 (ArC), 132.7 (ArC), 132.4 (ArC), 131.2 (ArC), 130.0 (ArC), 126.9 (ArC), 126.0 (ArC), 123.3 (ArC), 122.2 (ArC), 116.3 (ArC), 115.5 (ArC), and 67.9 (CH2). ESI-MS *m*/*z*: 270.01 ([M + H]^+^) and 292.00 ([M + Na]^+^).

*methyl 4-(benzo[d]thiazol-2-ylmethoxy)benzoate* (**6e**): Yellow solid; yield, 25.42%; m.p., 116–118 °C; ^1^H-NMR (500 MHz, CDCl3) δ 8.04 (d, *J* = 8.2 Hz, 1H, ArH), 8.01 (s, 1H, ArH), 8.00 (s, 1H, ArH), 7.88 (d, *J* = 7.9 Hz, 1H, ArH), 7.50 (t, *J* = 7.5 Hz, 1H, ArH), 7.40 (t, *J* = 7.4 Hz, 1H, ArH), 7.06 (d, *J* = 8.3 Hz, 2H, ArH × 2), 5.53 (s, 2H, CH2), 3.87 (s, 3H, CH3); and ESI-MS *m*/*z*: 300.68 ([M + H]^+^), 322.04 ([M + Na]^+^).

*ethyl 4-(benzo[d]thiazol-2-ylmethoxy)benzoate* (**6f**): White solid; yield, 36.42%; m.p., 118–120 °C; ^1^H-NMR (500 MHz, CDCl3) δ 8.04 (d, *J* = 8.2 Hz, 1H, ArH), 8.02 (s, 1H, ArH), 8.01 (s, 1H, ArH), 7.89 (d, *J* = 8.0 Hz, 1H, ArH), 7.50 (t, *J* = 7.7 Hz, 1H, ArH), 7.41 (t, *J* = 7.6 Hz, 1H, ArH), 7.05 (d, *J* = 8.8 Hz, 2H, ArH × 2), 5.54 (s, 2H, –CH2–O–), 4.34 (q, *J* = 7.1 Hz, 2H, -CH2-CH3), 1.36 (t, *J* = 7.1 Hz, 3H, CH3); 13C NMR (125 MHz, CDCl3) δ 167.8(C=O), 166.3 (–N=C–S–), 161.4 (ArC), 152.7 (ArC), 135.1 (ArC), 131.9 (ArC), 126.6 (ArC), 125.7 (ArC), 124.4 (ArC), 123.3 (ArC), 122.1 (ArC), 114.7 (ArC), 67.9 (–CH2–O–), 61.0 (–CH2–CH3), 14.6 (CH3); and ESI-MS *m*/*z*: 314.17 ([M + H]+), 336.75 ([M + Na]^+^).

*propyl 4-(benzo[d]thiazol-2-ylmethoxy)benzoate* (**6g**): White solid; yield, 48.93%; m.p., 106–108 °C; ^1^H-NMR (500 MHz, CDCl3) δ 8.04 (d, *J* = 12.0 Hz, 2H, ArH × 2), 8.01 (s, 1H, ArH), 7.89 (d, *J* = 8.0 Hz, 1H, ArH), 7.52 (d, *J* = 7.4 Hz, 1H, ArH), 7.41 (t, *J* = 7.6 Hz, 1H, ArH), 7.06 (d, *J* = 8.6 Hz, 2H, ArH × 2), 5.54 (s, 2H, –CH2–O–), 4.25 (t, *J* = 6.6 Hz, 2H, –CH2–CH2–CH3), 1.77 (dd, *J* = 14.2, 7.1 Hz, 2H, –CH2–CH2–CH3), 1.01 (t, *J* = 7.4 Hz, 3H, CH3); 13C NMR (125 MHz, CDCl3) δ 167.8 (C=O), 166.4 (–N=C–S–), 161.5 (ArC), 152.9 (ArC), 135.2 (ArC), 131.9 (ArC), 126.6 (ArC), 125.7 (ArC), 124.5 (ArC), 123.4 (ArC), 122.1 (ArC), 114.8 (ArC), 68.0 (–CH2–O–), 66.6 (–CH2–CH2–CH3), 22.4 (–CH2–CH2–CH3), 10.7 (CH3); HR-MS (ESI): *m*/*z* calcd. for C18H17NO3S: 328.1002; and found: 328.0972 [M + H]^+^.

*1-(2-(benzo[d]thiazol-2-ylmethoxy)phenyl)ethanone* (**6h**): Yellow solid; yield, 80.21%; m.p., 89–90 °C; ^1^H-NMR (500 MHz, CDCl3) δ 7.91 (m, 1H, ArH), 7.77 (dd, *J* = 7.6, 1.4 Hz, 1H, ArH), 7.49 (m, 3H, ArH × 3), 7.34(m, 1H, ArH), 7.06 (d, *J* = 8.7 Hz, 2H, ArH × 2), 5.57 (s, 2H, CH2), 2.74 (s, 3H, –COCH3); and ESI-MS *m*/*z*: 284.06 ([M + H]^+^), 306.10 ([M + Na]^+^).

*1-(3-(benzo[d]thiazol-2-ylmethoxy)phenyl)ethanone* (**6i**): Yellow solid; yield, 67.84%; m.p., 107–108 °C; ^1^H-NMR (500 MHz, CDCl3) δ 8.04 (d, *J* = 8.2 Hz, 1H, ArH), 7.90 (d, *J* = 8.0 Hz, 1H, ArH), 7.65 (s, 1H, ArH), 7.60 (d, *J* = 7.6 Hz, 1H, ArH), 7.51 (t, *J* = 7.5 Hz, 1H, ArH), 7.41 (t, *J* = 7.9 Hz, 2H, ArH × 2), 7.23 (t, *J* = 6.8Hz,1H, ArH), 5.54 (s, 2H, CH2), 2.60 (s, 3H, –COCH3); HR-MS (ESI): *m*/*z* calcd. for C16H13NO2S: 284.0740; and found: 284.0708 [M + H]^+^.

*1-(4-(benzo[d]thiazol-2-ylmethoxy)phenyl)ethanone* (**6j**): White solid; yield, 61.13%; m.p., 148–150 °C; ^1^H-NMR (500 MHz, CDCl3) δ 8.05 (d, *J* = 8.0 Hz, 1H, ArH), 7.96 (d, *J* = 8.7 Hz, 2H, ArH × 2), 7.90 (d, *J* = 8.2 Hz, 1H, ArH), 7.53 (d, *J* = 7.3 Hz, 1H, ArH), 7.42 (t, *J* = 7.6 Hz, 1H, ArH), 7.09 (d, *J* = 8.7 Hz, 2H, ArH × 2), 5.56 (s, 2H, CH2), 2.56 (s, 3H, –COCH3); and ESI-MS *m*/*z*: 284.15 ([M + H]^+^), 306.08 ([M + Na]^+^). 

*1-(4-(benzo[d]thiazol-2-ylmethoxy)-2-hydroxyphenyl)ethanone* (**6k**): White solid; yield, 37.46%; m.p., 128–130 °C; 1H NMR (500 MHz, CDCl3) δ 8.05 (d, *J* = 8.2 Hz, 1H, ArH), 7.91 (d, *J* = 8.0 Hz, 1H, ArH), 7.68 (s, 1H, ArH), 7.52 (t, *J* = 7.7 Hz, 1H, ArH), 7.43 (t, *J* = 7.6 Hz, 1H, ArH), 6.58 (m, 2H, ArH × 2), 6.40 (d, *J* = 12.5 Hz, 1H, ArH), 5.53 (s, 2H, CH2), 2.55 (d, *J* = 9.5 Hz, 3H, –COCH3); 13C NMR (125 MHz, CDCl3) δ 203.1(C=O), 167.6 (–N=C–S–), 165.3 (ArC), 164.1 (ArC), 152.6 (ArC), 133.3 (ArC), 132.9 (ArC), 126.8 (ArC), 125.9 (ArC), 123.3 (ArC), 122.1 (ArC), 115.1 (ArC), 107.8 (ArC), 102.8 (ArC), 67.8 (CH2), 26.5 (CH3); HR-MS (ESI): *m*/*z* calcd. for C16H13NO3S: 300.0689; and found: 300.0662 [M + H]^+^.

*4-(benzo[d]thiazol-2-ylmethoxy)-3-methoxybenzaldehyde* (**6l**): Yellow solid; yield, 46.15%; m.p., 110–112 °C; ^1^H-NMR (500 MHz, CDCl3) δ 9.84 (s, 1H, –CHO), 8.05 (d, *J* = 8.0 Hz, 1H, ArH), 7.89 (d, *J* = 7.8 Hz, 1H, ArH), 7.51 (t, *J* = 7.6 Hz, 1H, ArH), 7.45 (s, 1H, ArH), 7.41 (dd, *J* = 14.6, 7.7 Hz, 2H, ArH × 2), 7.12 (d, *J* = 8.0 Hz, 1H, ArH), 5.65 (s, 1H), 3.97 (s, 3H); and ESI-MS *m*/*z*: 300.37 ([M + H]^+^), 322.78 ([M + Na]^+^).

*2-((4-allyl-2-methoxyphenoxy)methyl)benzo[d]oxazole* (**6m**): White solid; yield, 41.8%; m.p., 54–56 °C; ^1^H-NMR (500 MHz, CDCl3) δ 8.04 (d, *J* = 8.1 Hz, 1H, ArH), 7.88 (d, *J* = 7.9 Hz, 1H, ArH), 7.49 (m, 1H, ArH), 7.39 (dd, *J* = 11.2, 4.0 Hz,1H, ArH), 6.95 (d, *J* = 8.1 Hz, 1H, ArH), 6.77 (s, 1H, ArH), 6.68 (d, *J* = 8.0 Hz, 1H, ArH × 2), 5.94 (dd, *J* = 16.9, 10.1 Hz, 1H, –CH=CH2), 5.54 (s, 2H, –CH2–O–), 5.09 (d, *J* = 1.3 Hz, 1H, –CH=CH2), 5.06 (d, *J* = 0.9 Hz, 1H, CH=CH2), 3.91 (s, 3H, CH3), 3.33 (d, *J* = 6.6 Hz, 2H, –CH2CH=CH2); ^13^C-NMR (125 MHz, CDCl3) δ 169.9 (–N=C–S–), 152.5 (ArC), 150.1 (ArC), 145.9 (ArC), 137.6 (ArC), 135.1 (–CH2CH=CH2), 126.5 (ArC), 125.5 (ArC), 123.1 (ArC), 122.1 (ArC), 120.8 (ArC), 116.1 (ArC), 115.6 (–CH2CH=CH2), 113.0 (ArC), 69.6 (–CH2–O–), 56.2 (CH3), 40.0 (–CH2CH=CH2); HR-MS (ESI): *m*/*z* calcd. for C18H17NO2S: 312.1053; and found: 312.1024 [M + H]^+^.

### 3.2. Antifungal Bioassay

According to our previous research [32,33,34], the mycelium growth rate method was employed to assess the fungicidal activities of the synthetic compounds against eight plant pathogenic fungi (*Fusarium oxysporum f.* sp. *niveum*, *Fusarium. graminearum*, *Fusarium. solani*, *Alternaria solani*, *Colletotrichum gloeosporioides*, *Valsa mali*, *Magnaporthe oryzae*, and *Botrytis cinerea*), which were provided by the Institute of Plant Disease, Northwest A and F University, China. The strains were retrieved from the storage tube and cultured for two weeks at 28 ± 0.5 °C on a potato dextrose agar (PDA). 

The specific methods are based on the literature [1]: PDA medium was prepared in the conical flasks and sterilized. The tested compounds were dissolved in acetone, making five concentrations with the gradients of 100, 50, 25, 12.5, and 6.25 μg/mL, before mixing with molten agar at 55 °C. The mixture was then poured into sterilized Petri dishes. The tested fungi were incubated in PDA at 28 ± 0.5 °C for one week for the antifungal assays. A hole puncher with a diameter of four mm was used to cut culture medium and vaccinated to the center of the PDA Petri dishes with a sterilized inoculation needle. The inoculated Petri dishes were incubated at 28 ± 0.5 °C for three-to-four days until the mycelia grew completely. Commercially available fungicide hymexazol was used as the positive control, while the solution of equal concentration of acetone was used as a negative control. Three repetitions were conducted for each sample and the diameters of the mycelia were measured four times by cross bracketing method. The inhibition rate was calculated according to the formula:

Inhibition rate (%) = (C − T)/(C − 4 mm) × 100%

where C is the average diameter of mycelia in the blank test, and T is the average diameter of mycelia on treated PDA with those compounds.

The inhibition ratio of those compounds at the concentration of 100 μg/mL was shown in Table 1. The compounds with more than 60% inhibition rates were selected for further comparison and IC_50_ (median inhibitory concentration) values of rates were determined. The experimental data of the fungicidal activities were analyzed using software of excel from Windows to give the results of IC_50_ values, as shown in Table 2.

### 3.3. Molecular Docking Studies

The crystal structure the lipid transfer protein sec14p from *Saccharomyces cerevisiae* (PDB 6F0E) was provided from the Brookhaven protein data bank (PDB; http://www.rcsb.org/pdb). Docking studies were performed by using AutoDock 4.2 software (The Scripps Research Institute, California, CA, USA), as in our previous studies [35]. The preparations relevant to Autodock docking were done using the Autodock Tools. The grid box (40 × 40 × 40) was set according to the active site. The docking parameters consisted of setting the population size to 150, the number of generations to 270,000, and the number of evaluations to 25,000,000 while the number of docking runs was set to 40 with other default values during each docking run. Docking calculations were carried with the Lamarkian Genetic Algorithm (LGA). The analysis of results was carried out by USCF Chimera software (the Computer Graphics Laboratory, California, CA, USA), which was downloaded from http://www.cgl.ucsf.edu/chimera.

## 4. Conclusions

In the present study, a series of benzoxazole and benzothiazole derivatives were prepared and their fungicidal activities against eight plant pathogens were tested. Most of the test compounds, i.e., **5a**, **5b**, **5h,** and **5i**, were found to be more active against two fungi, *F. solani* and *B. cinerea*, than the other six organisms. **5a**, **5b**, **5h**, and **5i** were found to be most efficient antifungal compounds and may be new leads for the development of new fungicides. Furthermore, from the present results, it appears that benzoxazoles have an advantage over benzothiazoles as a template in antifungal potency. 

## Figures and Tables

**Table 1:** Preliminary Antifungal Activities of Compounds at 100 μg/mL ^a^.

Cpd.	Average Values of Inhibition Rate (%) to Eight Pathogens							
	*F. n.*	*F. g.*	*F. s.*	*A. s.*	*C. g.*	*V. m.*	*M. o.*	*B. c.*
**5a**	85	87	100	92	100	100	95	100
**5b**	51	30	85	69	70	43	72	80
**5c**	44	30	41	57	37	39	44	68
**5d**	38	45	43	34	57	44	38	78
**5e**	44	38	41	37	33	46	25	33
**5f**	28	34	57	43	49	49	44	62
**5g**	26	30	47	57	45	36	53	61
**5h**	62	30	89	69	56	80	55	72
**5i**	59	30	87	63	61	90	81	79
**5j**	39	49	78	43	57	53	50	33
**5k**	18	24	23	9	25	20	19	44
**5l**	33	40	23	29	45	29	34	39
**5m**	35	27	58	56	47	72	50	58
**6a**	35	56	49	36	47	36	39	93
**6b**	28	30	34	26	29	36	25	36
**6c**	21	32	28	34	57	19	72	67
**6d**	16	32	51	16	35	43	34	45
**6e**	33	18	28	5	22	38	16	22
**6f**	8	30	23	9	18	12	6	33
**6g**	3	20	32	3	14	53	62	11
**6h**	39	71	69	28	81	46	44	52
**6i**	20	24	19	32	32	16	25	52
**6j**	18	28	23	9	18	9	20	28
**6k**	1	14	34	16	26	22	24	59
**6l**	31	28	34	46	25	12	25	81
**6m**	16	56	50	20	32	36	48	59
Hy	69	71	88	63	20	16	100	100

^a^ Note: *F. n.*, *Fusarium oxysporum f.* sp. *niveum*; *F. g.*, *Fusarium graminearum*; *F. s.*, *Fusarium solani*; *A. s.*, *Alternaria solani*; *C. g.*, *Colletotrichum gloeosporioides*; *V. m.*, *Valsa mali*; *M. o.*, *Magnaporthe oryzae*; *B. c.*, *Botrytis cinerea*; and Hy, hymexazol.

**Table 2:** IC_50_ values of Some Compounds on Eight Phytopathogenic Fungi ^a^.

Cpd.	IC_50_ ± SD/(μg/mL)							
	*F. n.*	*F. g.*	*F. s.*	*A. s.*	*C. g.*	*V. m.*	*M. o.*	*B. c.*
**5a**	45.00 ± 2.71	20.59 ± 2.34	12.27 ± 0.88	23.23 ± 1.58	25.94 ± 2.53	23.52 ± 1.63	65.25 ± 2.98	19.92 ± 3.34
**5b**	>100	>100	15.98 ± 1.15	32.10 ± 7.11	32.78 ± 2.11	>100	30.61 ± 0.73	23.78 ± 1.55
**5c**	>100	>100	>100	>100	>100	>100	>100	50.04 ± 6.03
**5f**	>100	>100	>100	>100	>100	>100	>100	73.04 ± 3.54
**5g**	>100	>100	>100	>100	>100	>100	>100	77.41 ± 3.16
**5h**	94.48 ± 2.28	>100	4.34 ± 0.13	33.32 ± 2.82	>100	31.34 ± 2.64	>100	35.52 ± 2.95
**5i**	>100	>100	17.61 ± 0.93	73.09 ± 4.10	83.35 ± 1.04	37.98 ± 2.75	73.62 ± 5.12	42.01 ± 2.76
**5j**	>100	>100	16.53 ± 1.89	>100	>100	>100	>100	>100
**5m**	>100	>100	>100	>100	>100	59.65 ± 5.0	>100	>100
**6a**	>100	>100	>100	>100	>100	>100	>100	62.62 ± 2.17
**6c**	>100	>100	>100	>100	>100	>100	>100	61.20 ± 4.91
**6h**	>100	23.39 ± 1.29	15.55 ± 1.35	>100	29.61 ± 1.39	>100	>100	>100
**6l**	>100	>100	>100	>100	>100	>100	>100	50.42 ± 5.75
Hy	8.47 ± 1.72	59.93 ± 2.19	38.92 ± 3.17	54.16 ± 1.46	>100	>150	35.40 ± 2.38	4.72 ± 0.52

^a^ Note: The results were expressed as the mean ± SD of triplicate experiments.

**Table 3:** The docking results of the tested compounds.

Cpd.	Binding Energy (kcal/mol)	Inhibit Constant (μM)
**5a**	−6.89	8.92
**5b**	−6.86	9.39
**5h**	−7.38	3.9
**5i**	−7.66	2.44
**5j**	−7.85	1.78
**6h**	−7.76	2.06
PIA ^a^	−6.96	7.97 (9.4) ^c^
BEA ^b^	−7.54	2.99 (1.7) ^c^

^a^ The inhibitor co-crystals with Sec14p from *S. cerevisiae* in the crystal structure of the protein complex (PDB code: 6F0E). ^b^ The benzamide compound 2 in the reference. ^c^ The value in parenthesis are IC_50_ for the active compound against Sec14p from *S. cerevisiae*.

## Supplementary Materials

The following are available on line. ^1^H-NMR, ^13^C-NMR and mass spectroscopy (MS) of compounds **5a**–**m** and **6a**–**m**.
