# Supplementary material for: Synthesis, Antifungal Activities and Molecular Docking Studies of Benzoxazole and Benzothiazole Derivatives
Source: Molecules. 2018 Sep 25;23(10):2457. doi: 10.3390/molecules23102457 (PMC6222379; doi:10.3390/molecules23102457)
Supplement: Supplementary file 1 [file molecules-23-02457-s001.pdf]

Synthesis, Antifungal Activities and Molecular Docking Studies of Benzoxazole and Benzothiazole Derivatives

Bo Luo [1], Ding Li [2,*], An-Ling Zhang [2] and Jin-Ming Gao [2,*]

[1] College of Life Sciences, Xinyang Normal University, Xinyang, Henan, 464000, China

[2] Shaanxi Key Labotory of Natural Products & Chemical Biology, Shaanxi Engineering Center of Bioresource Chemistry & Sustainable Utilization, College of Chemistry & Pharmacy, Northwest A&F University, Yangling 712100, P. R. China

* Correspondence: jbolid@nwsuaf.edu.cn (D. Li); jinminggao@nwsuaf.edu.cn (J.-M. Gao.);

Tel: +86-29-87092335; Fax: +86-29-87092335;

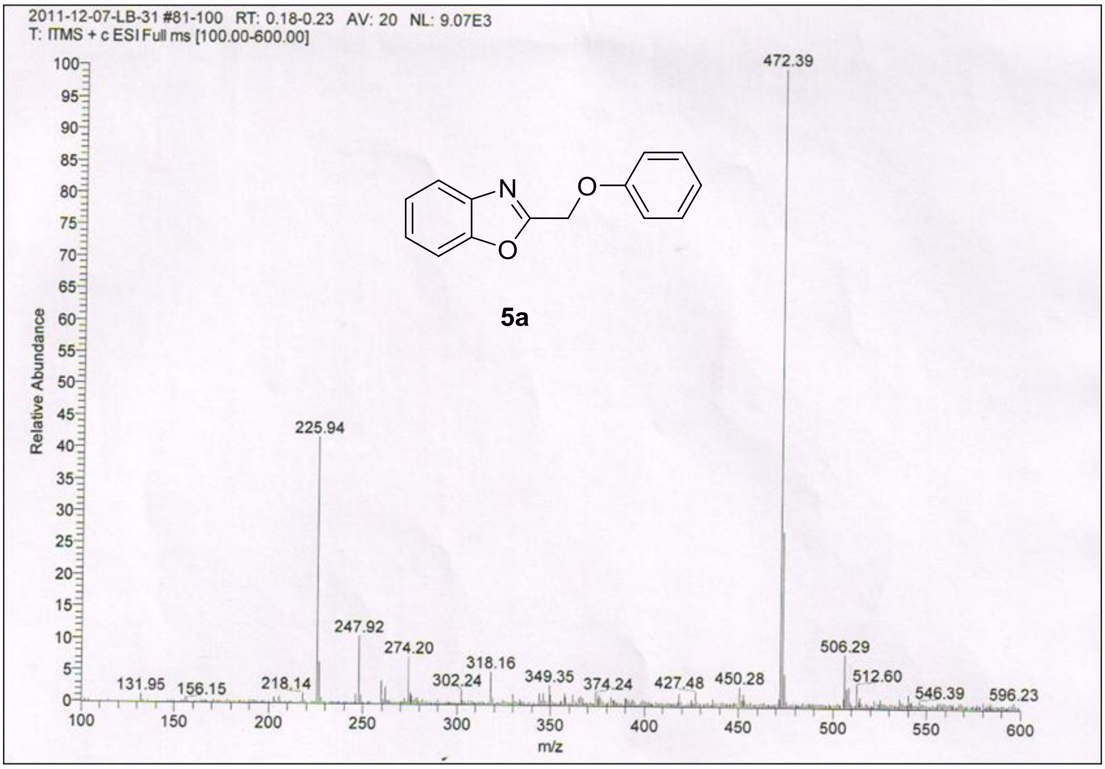
2011-12-07-LB-31 #81-100 RT: 0.18-0.23 AV: 20 NL: 9.07E3
T: ITMS + c ESI Full ms [100.00-600.00]
472.39
225.94
247.92
274.20
318.16
302.24
349.35
374.24
427.48
450.28
506.29
512.60
546.39
596.23
131.95
156.15
218.14
Relative Abundance
m/z
N
O
5a

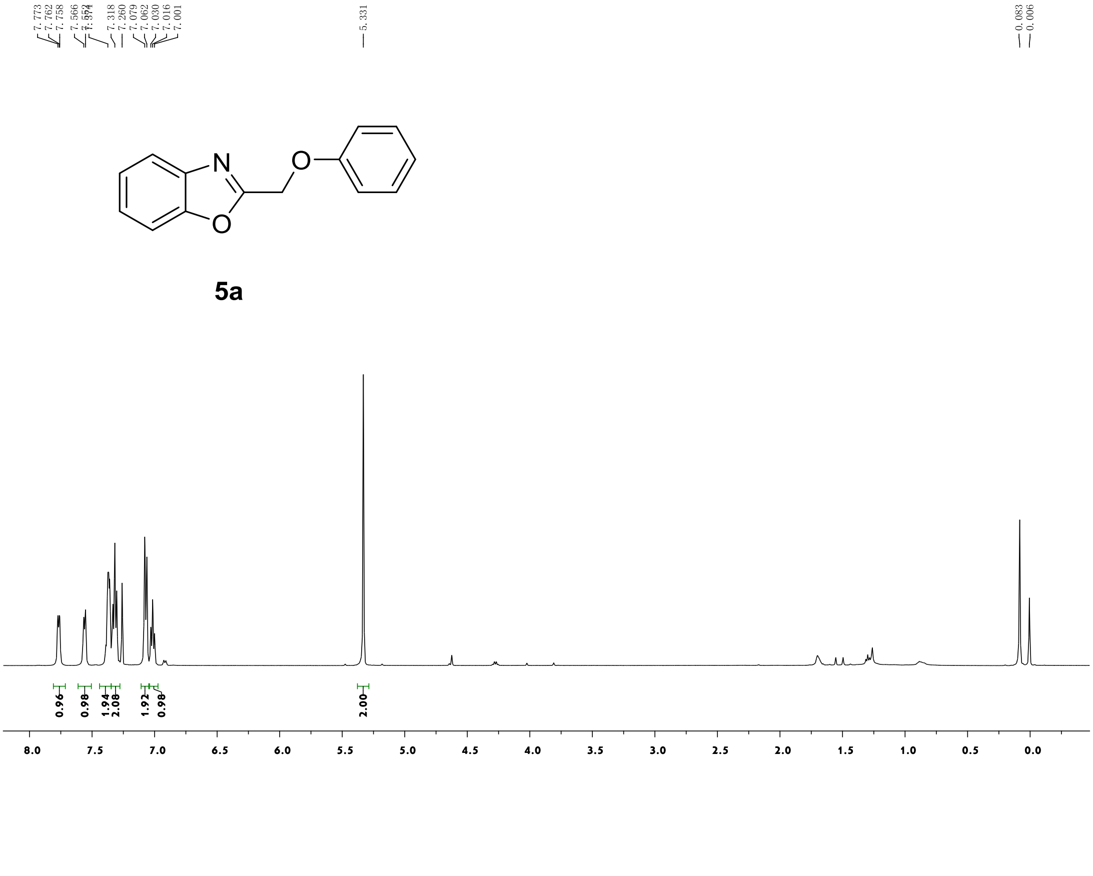
7.773
7.762
7.758
7.566
7.318
7.280
7.079
7.062
7.030
7.016
7.001
5.331
0.083
0.006
N
O
5a
0.96
0.98
1.94
2.08
1.92
0.98
2.00

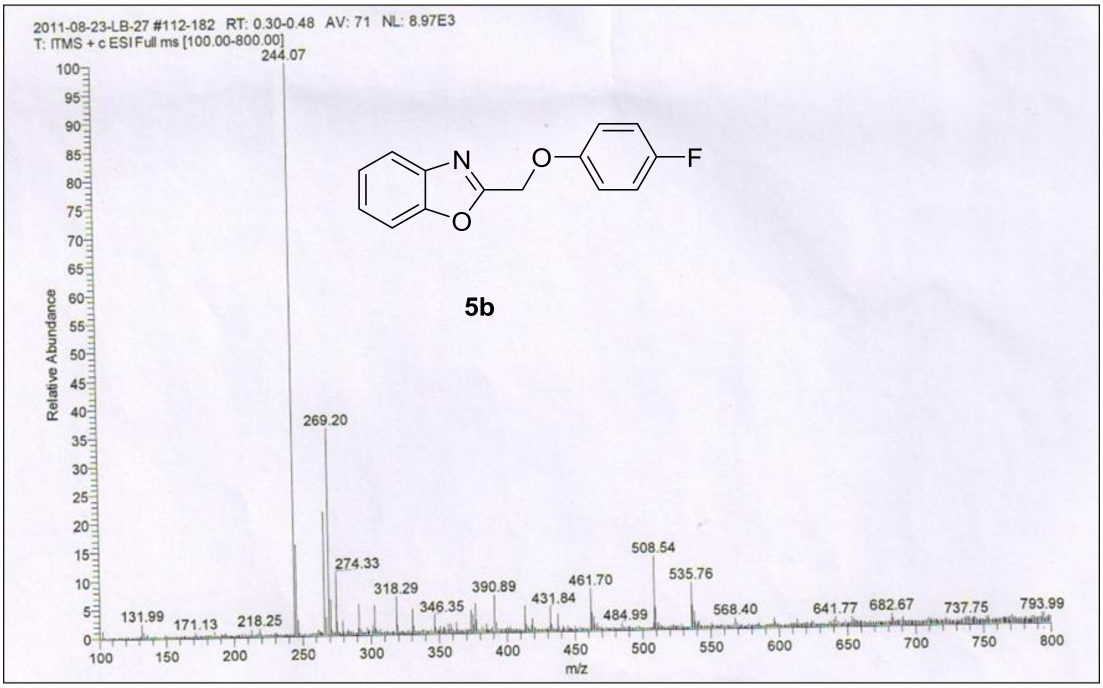

2011-08-23-LB-27 #112-182 RT: 0.30-0.48 AV: 71 NL: 8.97E3
T: ITMS + c ESI Full ms [100.00-800.00]
244.07
269.20
274.33
318.29
346.35
390.89
431.84
461.70
484.99
508.54
535.76
568.40
641.77
682.67
737.75
793.99
131.99
171.13
218.25
Relative Abundance
m/z
N
O
O
F
5b

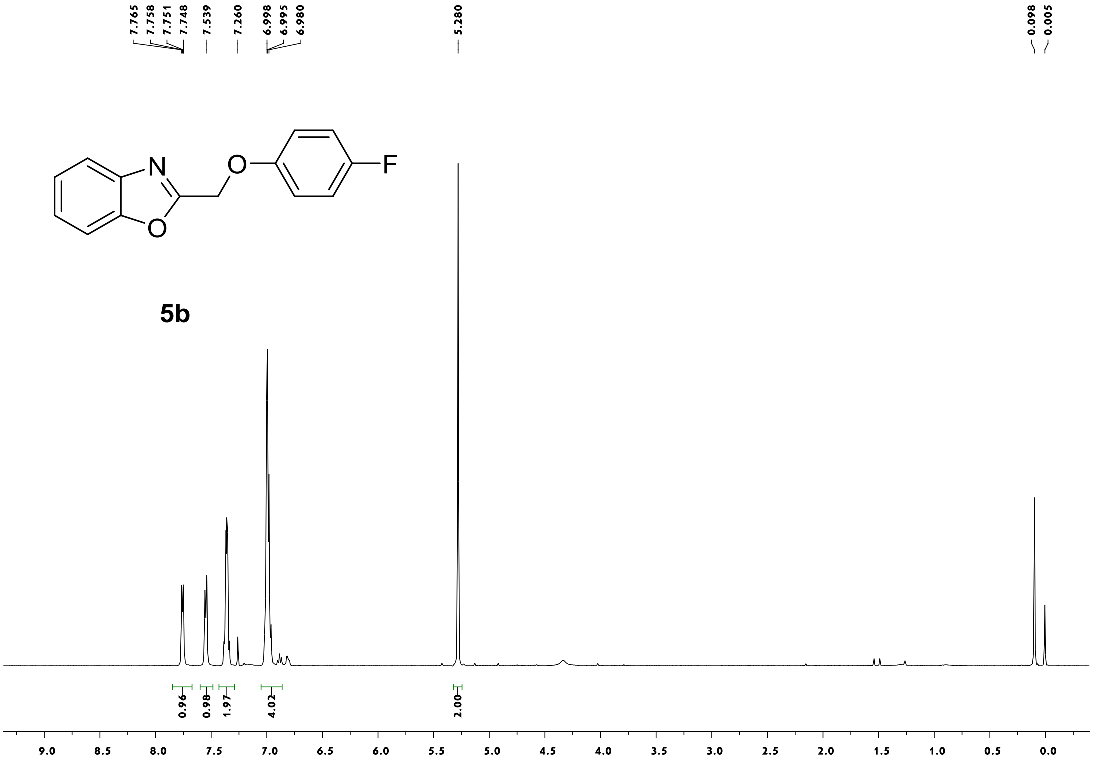

7.765
7.758
7.751
7.748
7.539
7.260
6.998
6.995
6.980
5.280
0.098
0.005
N
O
O
F
5b
0.96
0.98
1.97
4.02
2.00

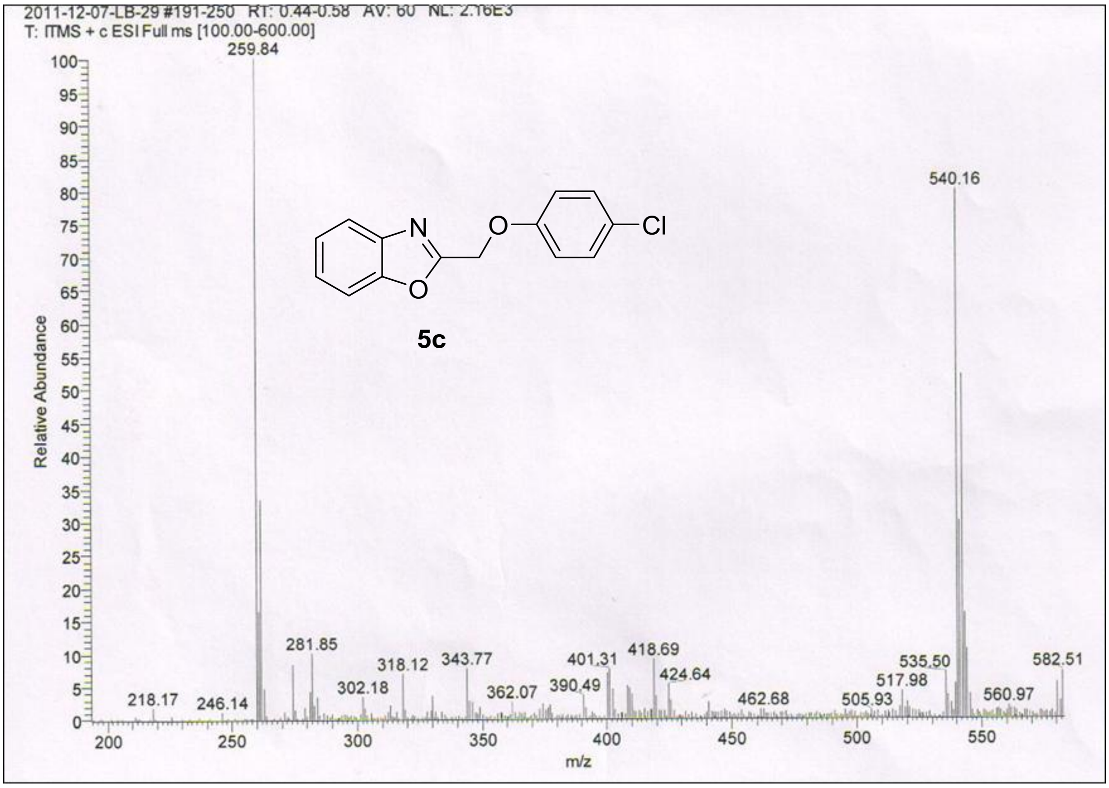
2011-12-07-LB-29 #191-250 RT: 0.44-0.58 AV: 60 NL: 2.16E3
T: ITMS + c ESI Full ms [100.00-600.00]
259.84
540.16
281.85
318.12
343.77
302.18
401.31
418.69
424.64
362.07
390.49
218.17
246.14
462.68
505.93
517.98
535.50
560.97
582.51
Relative Abundance
m/z
N
O
Cl
5c

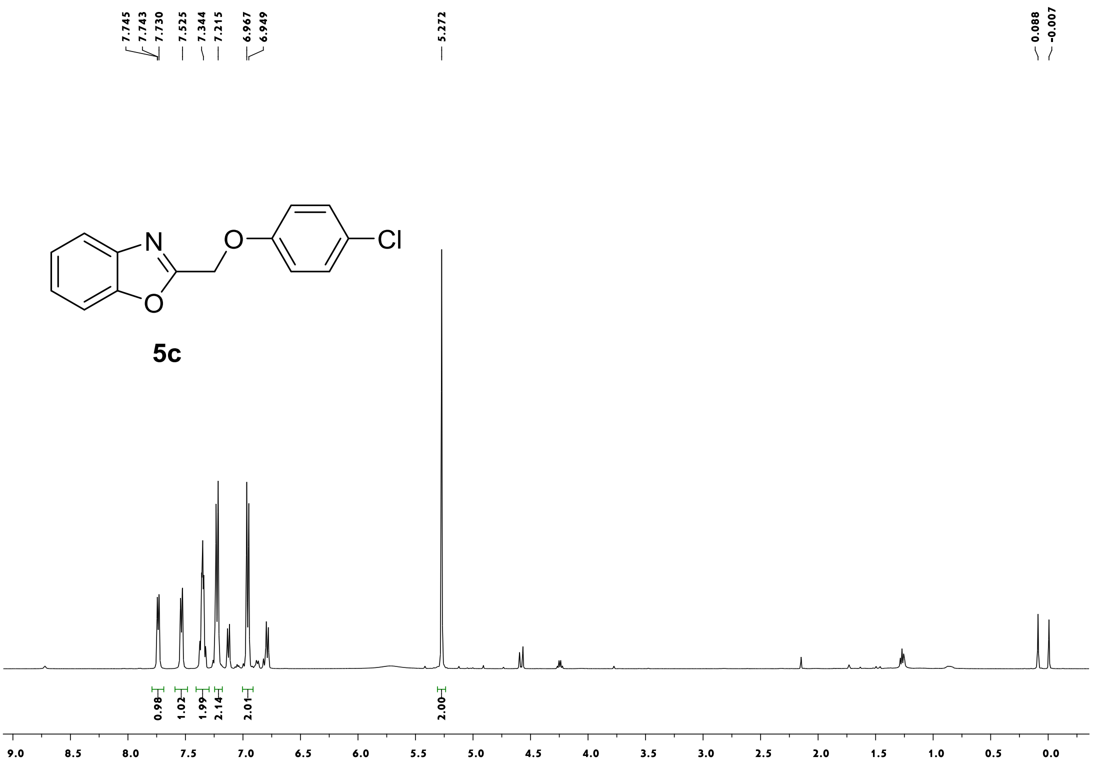
7.745
7.743
7.730
7.525
7.344
7.215
6.967
6.949
5.272
0.088
-0.007
N
O
Cl
5c
0.98
1.02
1.99
2.14
2.01
2.00
9.0
8.5
8.0
7.5
7.0
6.5
6.0
5.5
5.0
4.5
4.0
3.5
3.0
2.5
2.0
1.5
1.0
0.5
0.0

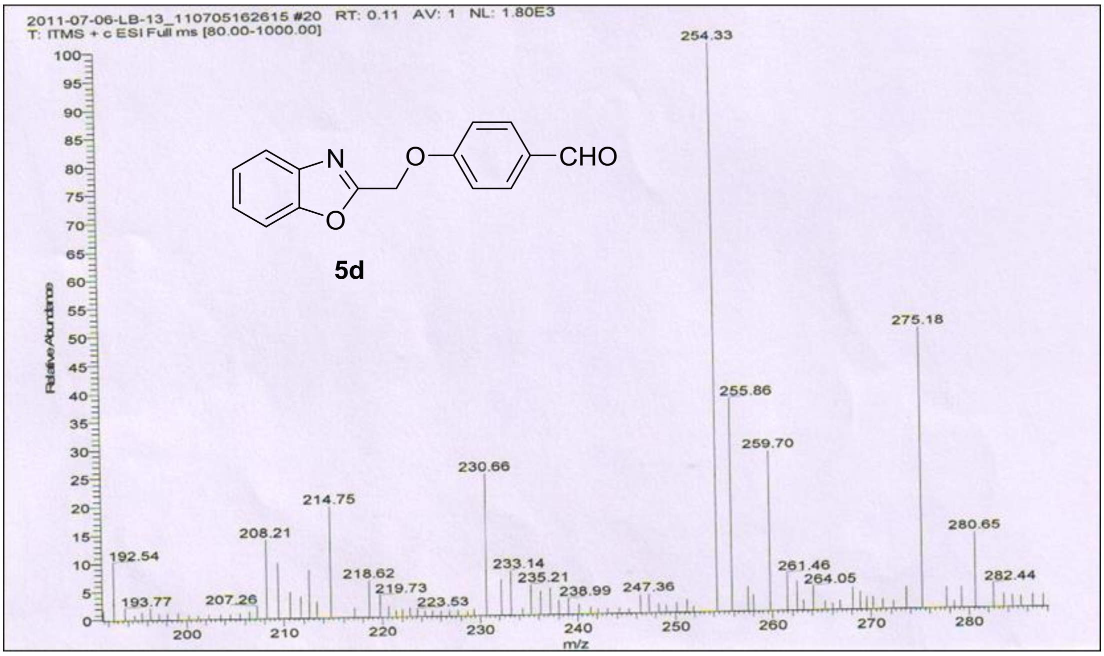
2011-07-06-LB-13_110705162615 #20 RT: 0.11 AV: 1 NL: 1.80E3
T: ITMS + c ESI Full ms [80.00-1000.00]
254.33
275.18
255.86
259.70
230.66
214.75
208.21
280.65
192.54
233.14
261.46
218.62
235.21
264.05
282.44
219.73
238.99
247.36
193.77
207.26
223.53
CHO
5d
Relative Abundance
m/z

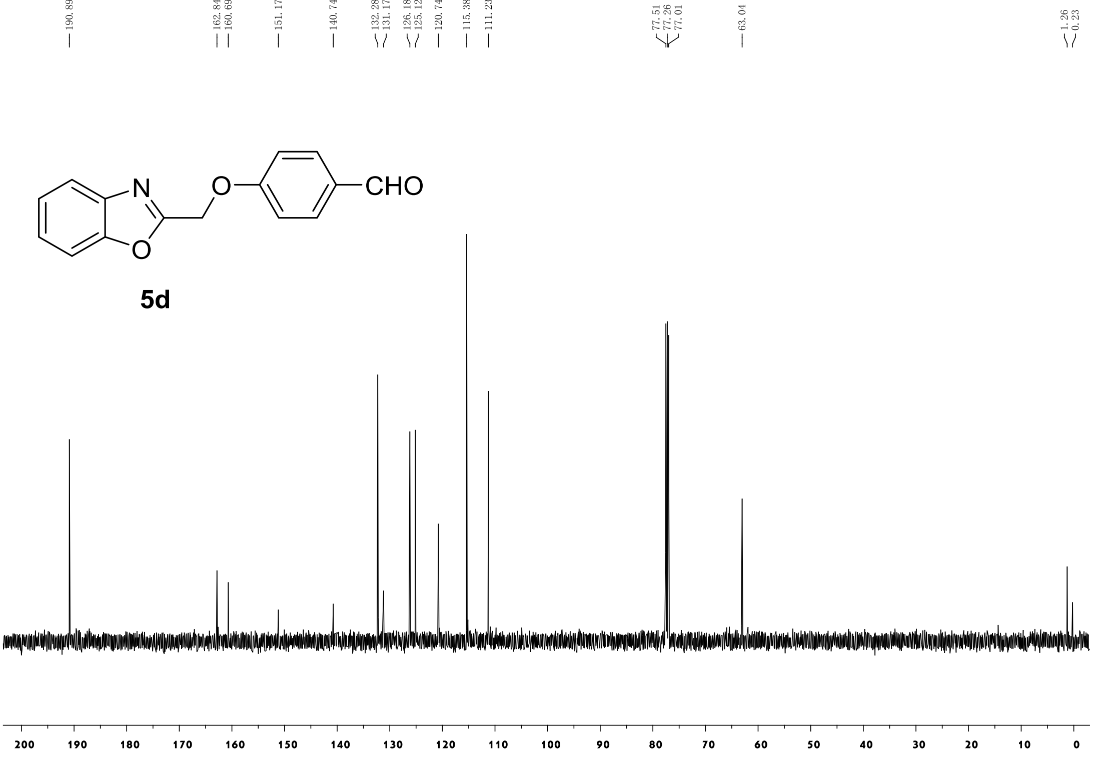
190.89
162.84
160.69
151.17
140.74
132.28
131.17
126.18
125.12
120.74
115.38
111.23
77.51
77.26
77.01
63.04
1.26
0.23
CHO
5d

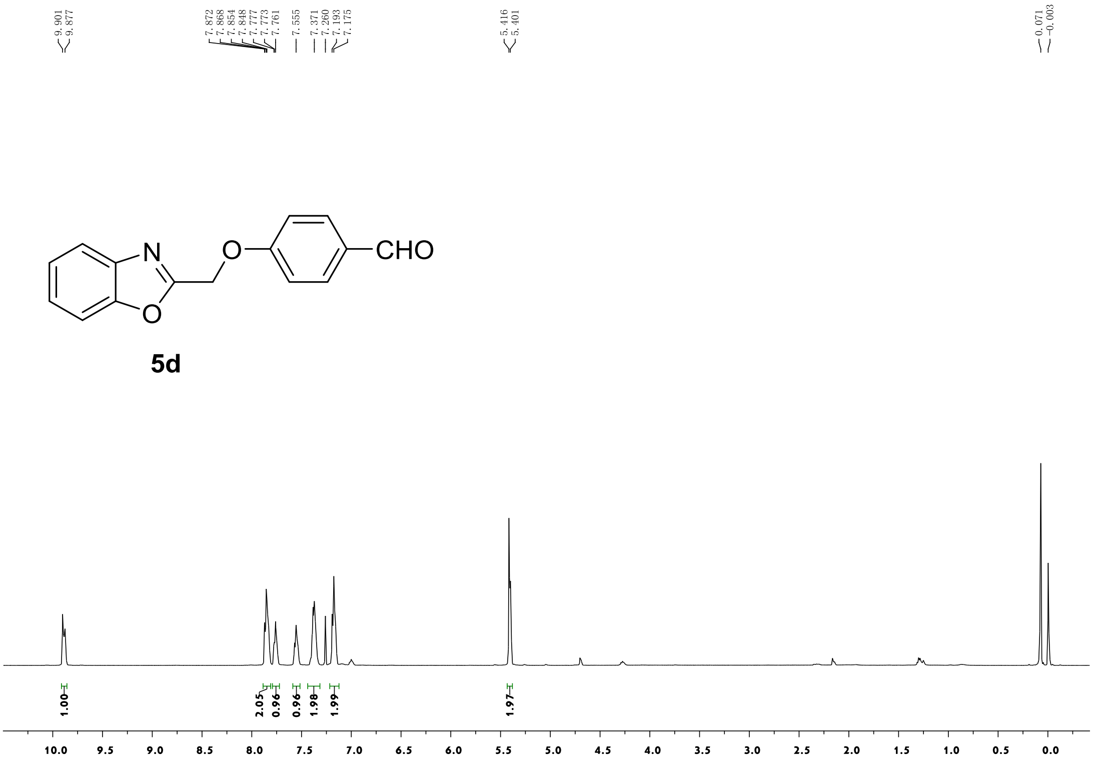
9.901
9.877
7.872
7.868
7.854
7.848
7.777
7.773
7.761
7.555
7.371
7.260
7.193
7.175
5.416
5.401
0.071
-0.003
N
O
CHO
5d
1.00
2.05
0.96
0.96
1.98
1.99
1.97
10.0 9.5 9.0 8.5 8.0 7.5 7.0 6.5 6.0 5.5 5.0 4.5 4.0 3.5 3.0 2.5 2.0 1.5 1.0 0.5 0.0

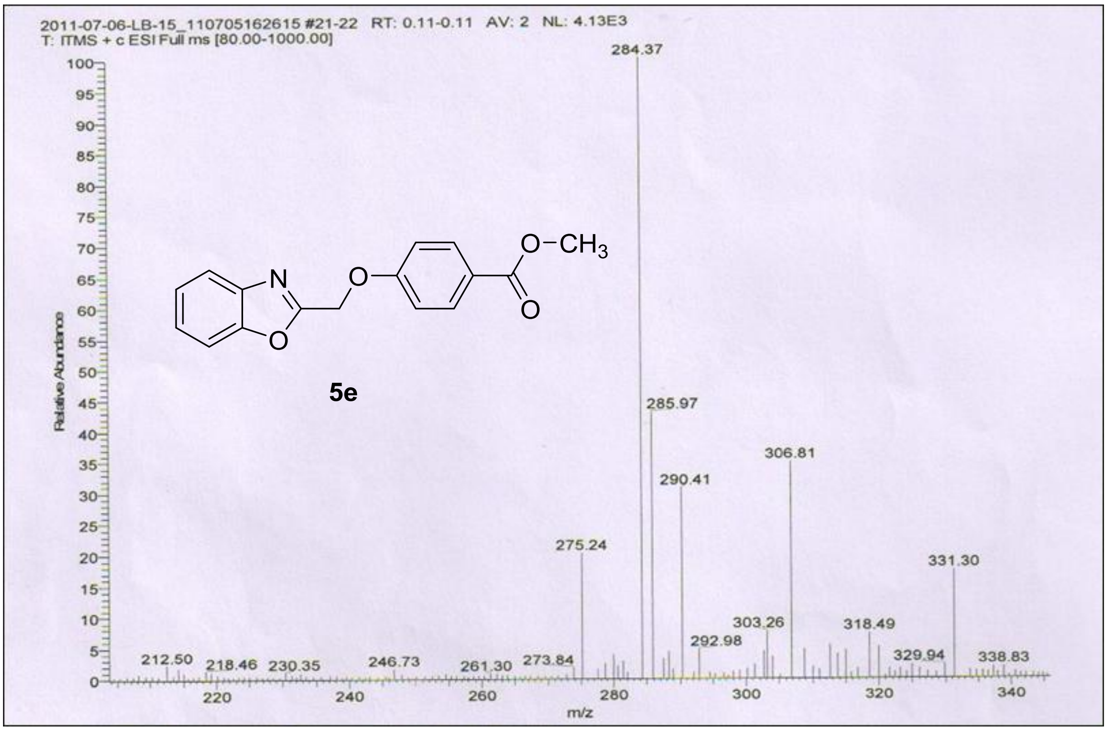
2011-07-06-LB-15_110705162615 #21-22 RT: 0.11-0.11 AV: 2 NL: 4.13E3
T: ITMS + c ESI Full ms [80.00-1000.00]
284.37
285.97
306.81
290.41
275.24
331.30
303.26
318.49
292.98
212.50 218.46 230.35 246.73 261.30 273.84 329.94 338.83
N
O
O−CH3
5e
Relative Abundance
m/z

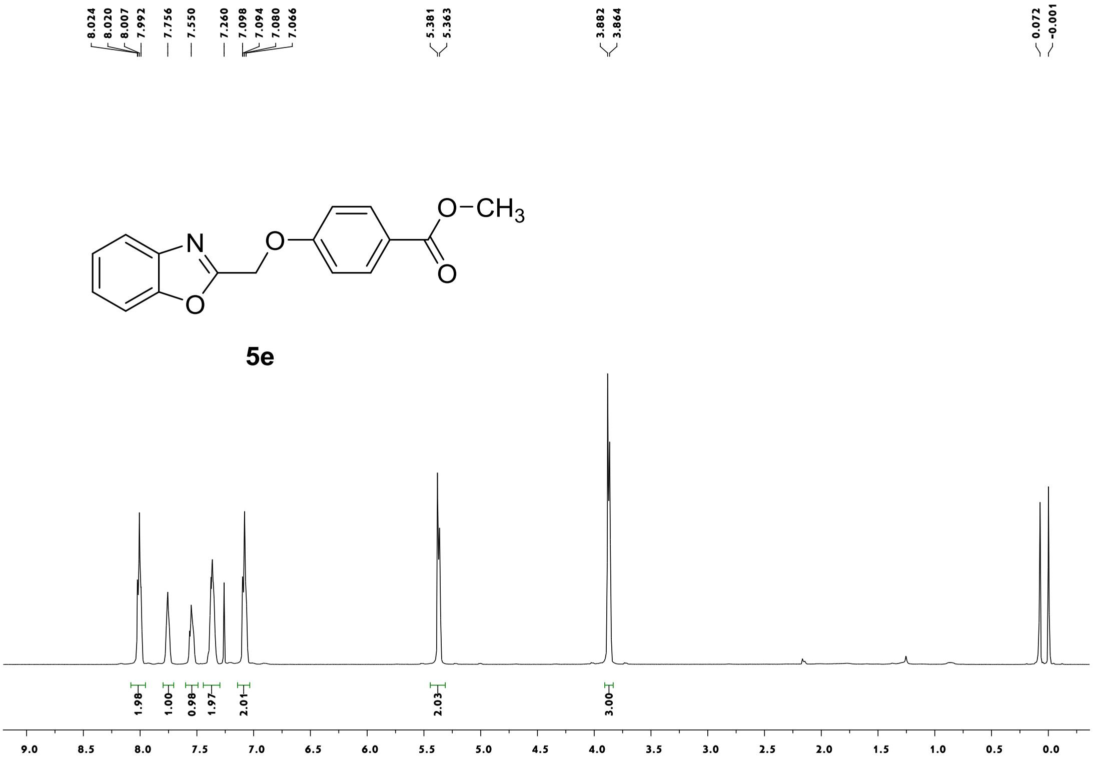
8.024
8.020
8.007
7.992
7.756
7.550
7.260
7.098
7.094
7.080
7.066
5.381
5.363
3.882
3.864
0.072
-0.001
O−CH3
N
O
O
O
5e
1.98
1.00
0.98
1.97
2.01
2.03
3.00
9.0 8.5 8.0 7.5 7.0 6.5 6.0 5.5 5.0 4.5 4.0 3.5 3.0 2.5 2.0 1.5 1.0 0.5 0.0

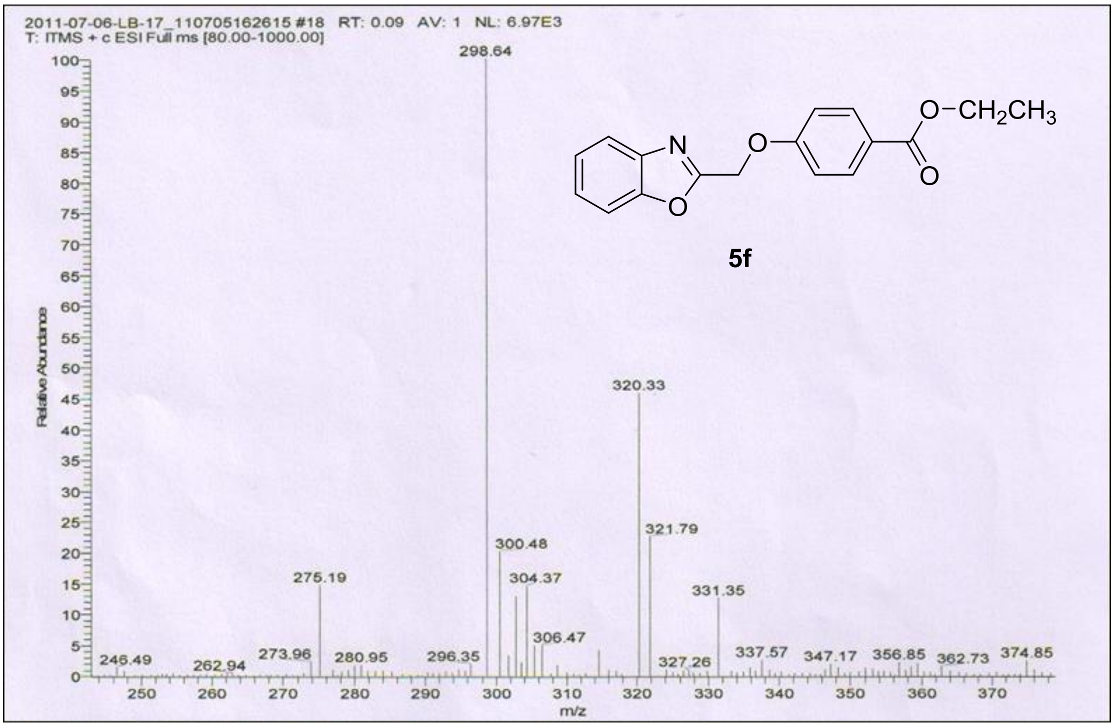
2011-07-06-LB-17_110705162615 #18 RT: 0.09 AV: 1 NL: 6.97E3
T: ITMS + c ESI Full ms [80.00-1000.00]
Relative Abundance
m/z
298.64
320.33
321.79
300.48
304.37
275.19
331.35
306.47
246.49
262.94
273.96
280.95
296.35
327.26
337.57
347.17
356.85
362.73
374.85
O−CH2CH3
N
O
O
O
5f

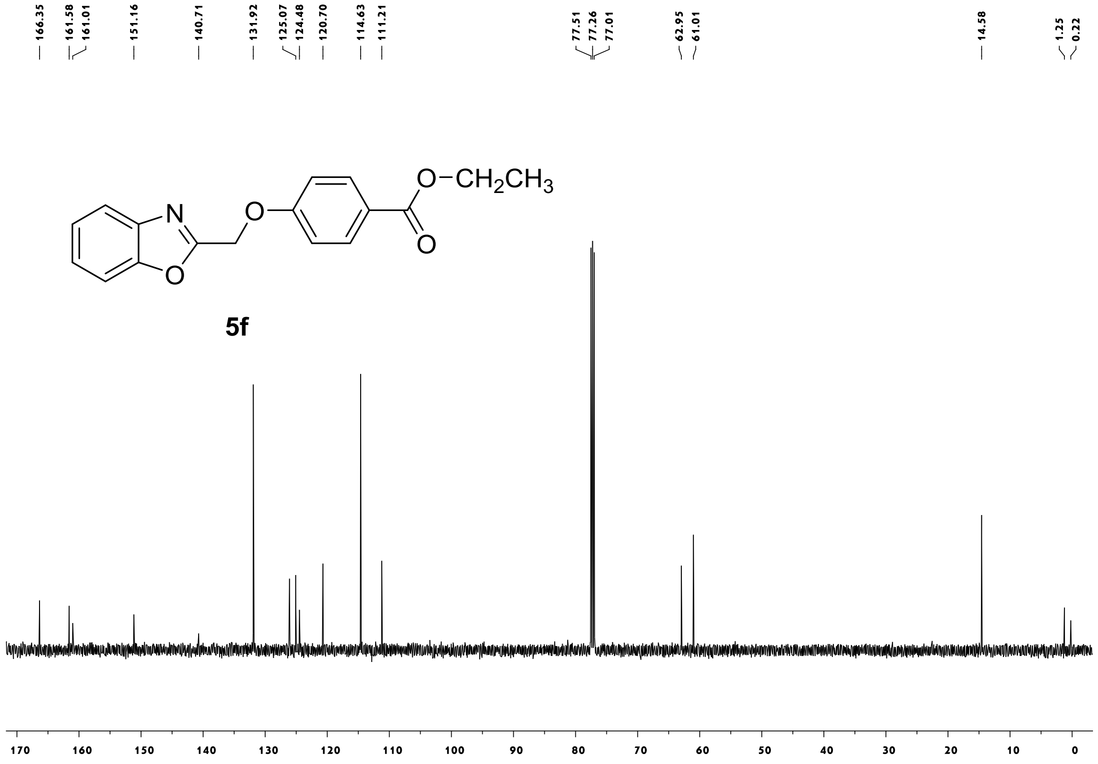
166.35
161.58
161.01
151.16
140.71
131.92
125.07
124.48
120.70
114.63
111.21
77.51
77.26
77.01
62.95
61.01
14.58
1.25
0.22
O−CH2CH3
N
O
O
O
5f
170 160 150 140 130 120 110 100 90 80 70 60 50 40 30 20 10 0

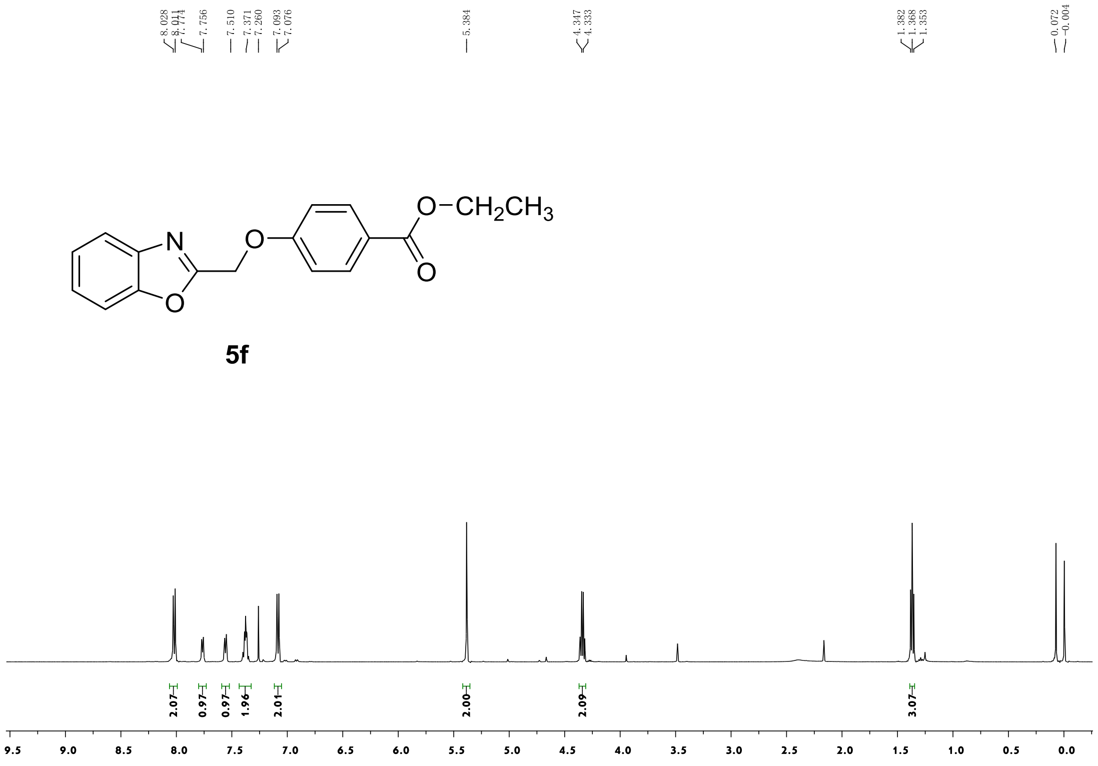
8.028
8.011
7.774
7.756
7.510
7.371
7.260
7.093
7.076
5.384
4.347
4.333
1.382
1.368
1.353
0.072
−0.004
O−CH2CH3
N
O
O
O
5f
2.07
0.97
0.97
1.96
2.01
2.00
2.09
3.07
9.5 9.0 8.5 8.0 7.5 7.0 6.5 6.0 5.5 5.0 4.5 4.0 3.5 3.0 2.5 2.0 1.5 1.0 0.5 0.0

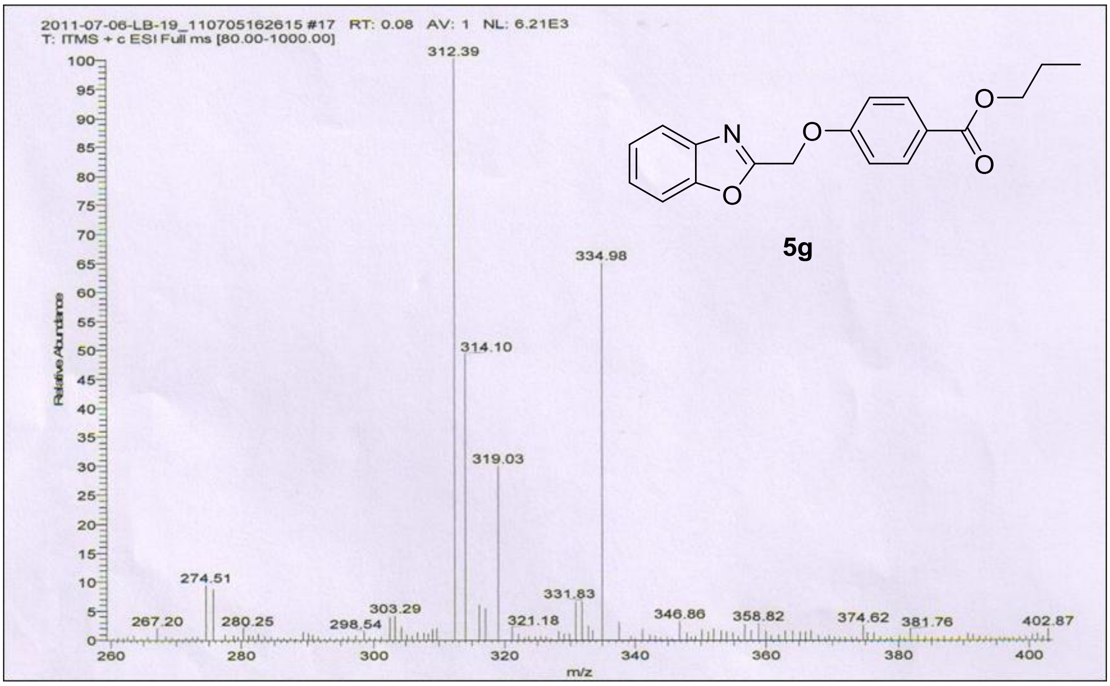
2011-07-06-LB-19_110705162615 #17 RT: 0.08 AV: 1 NL: 6.21E3
T: ITMS + c ESI Full ms [80.00-1000.00]
Relative Abundance
m/z
312.39
334.98
314.10
319.03
274.51
331.83
303.29
267.20
280.25
298.54
321.18
346.86
358.82
374.62
381.76
402.87
5g

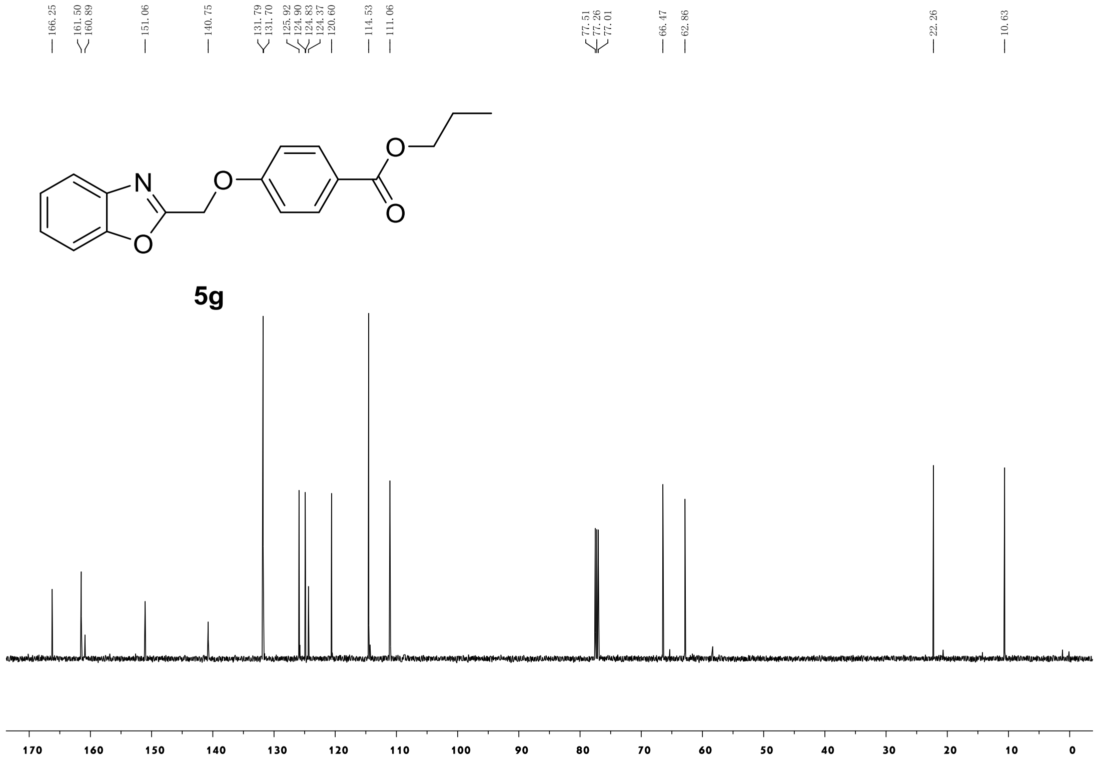
166.25
161.50
160.89
151.06
140.75
131.79
131.70
125.92
124.90
124.83
124.37
120.60
114.53
111.06
77.51
77.26
77.01
66.47
62.86
22.26
10.63
5g

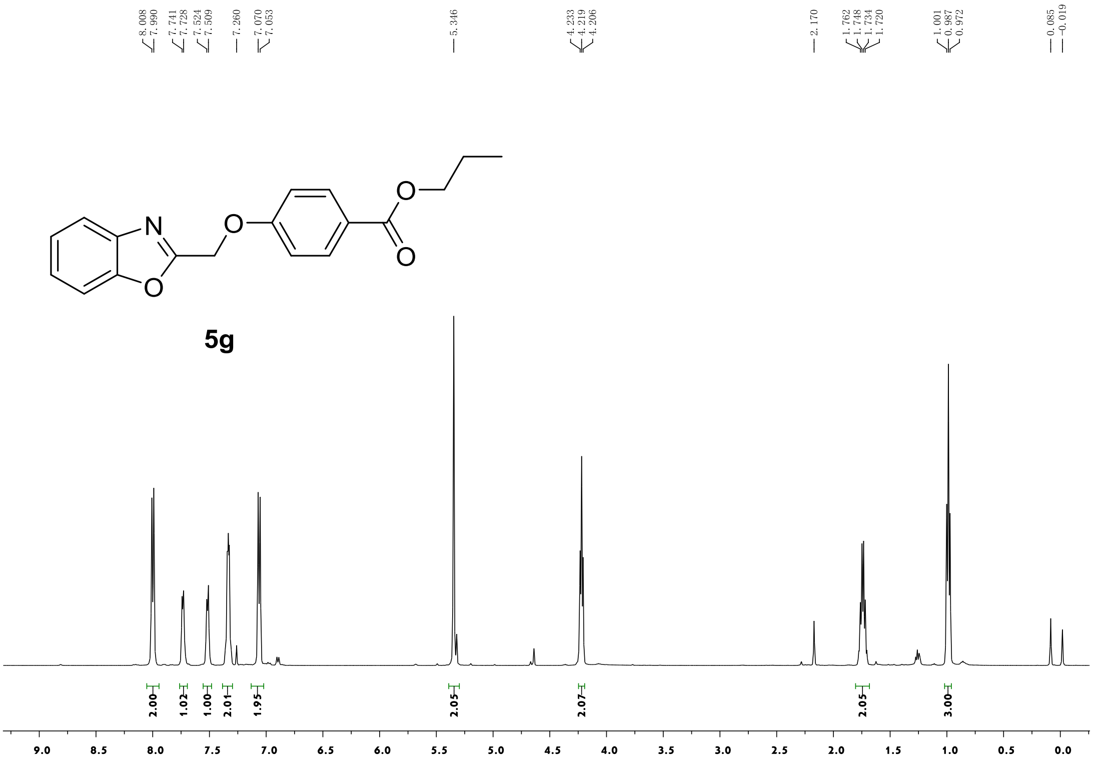
8.008
7.990
7.741
7.728
7.524
7.509
7.260
7.070
7.053
5.346
4.233
4.219
4.206
2.170
1.762
1.748
1.734
1.720
1.001
0.987
0.972
0.085
-0.019
N
O
O
O
O
2.00
1.02
1.00
2.01
1.95
2.05
2.07
2.05
3.00
9.0 8.5 8.0 7.5 7.0 6.5 6.0 5.5 5.0 4.5 4.0 3.5 3.0 2.5 2.0 1.5 1.0 0.5 0.0

5g

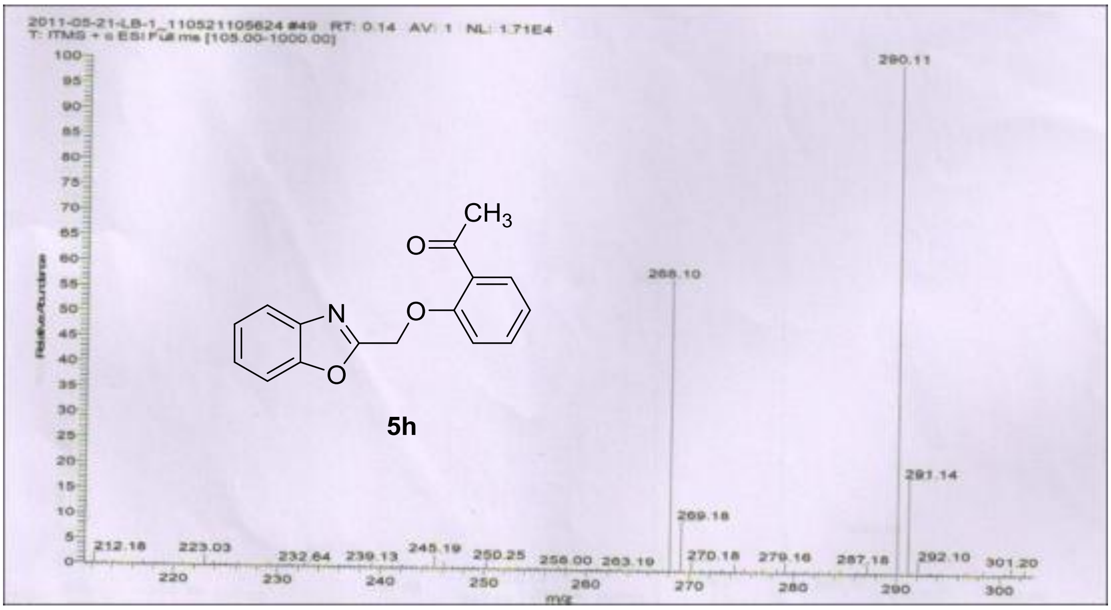
2011-05-21-LB-1_110521105624 #49 RT: 0.14 AV: 1 NL: 1.71E4
T: ITMS + c ESI Full ms [105.00-1000.00]
Relative Abundance
m/z
290.11
268.10
291.14
269.18
212.18
223.03
232.64
239.13
245.19
250.25
258.00
263.19
270.18
279.16
287.18
292.10
301.20
CH3
O
N
O
O

5h

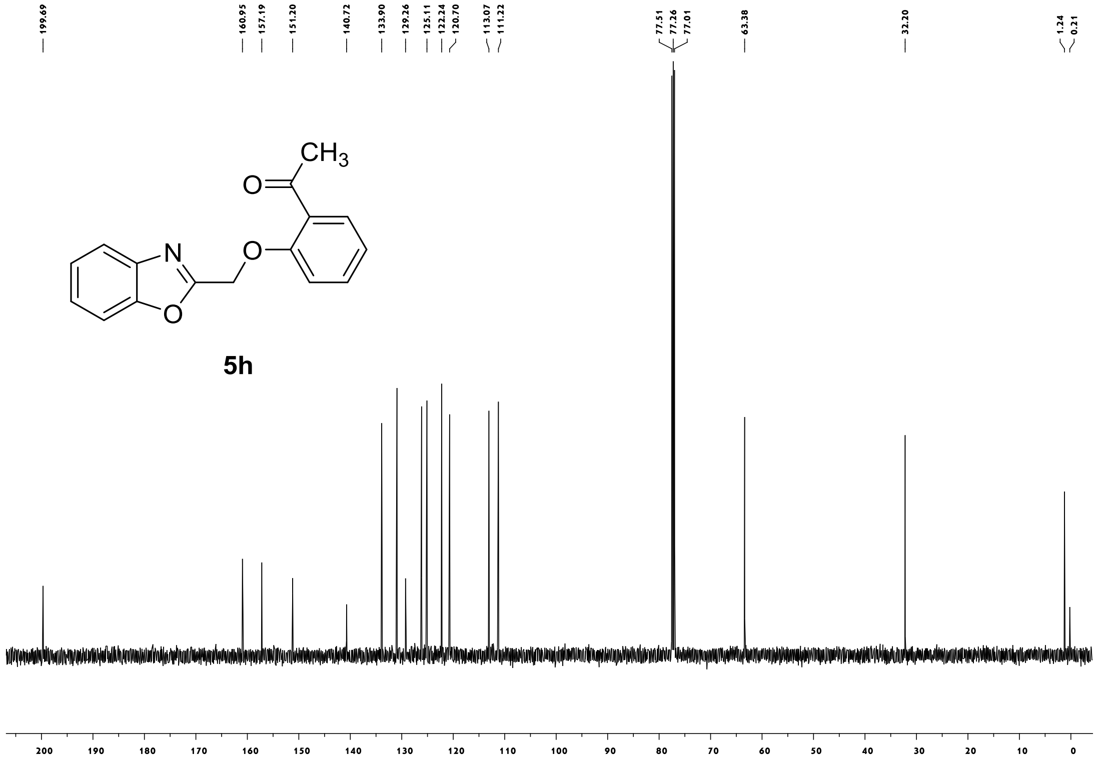
199.69
160.95
157.19
151.20
140.72
133.90
129.26
125.11
122.24
120.70
113.07
111.22
77.51
77.26
77.01
63.38
32.20
1.24
0.21
CH3
O
N
O
O
5h
200 190 180 170 160 150 140 130 120 110 100 90 80 70 60 50 40 30 20 10 0

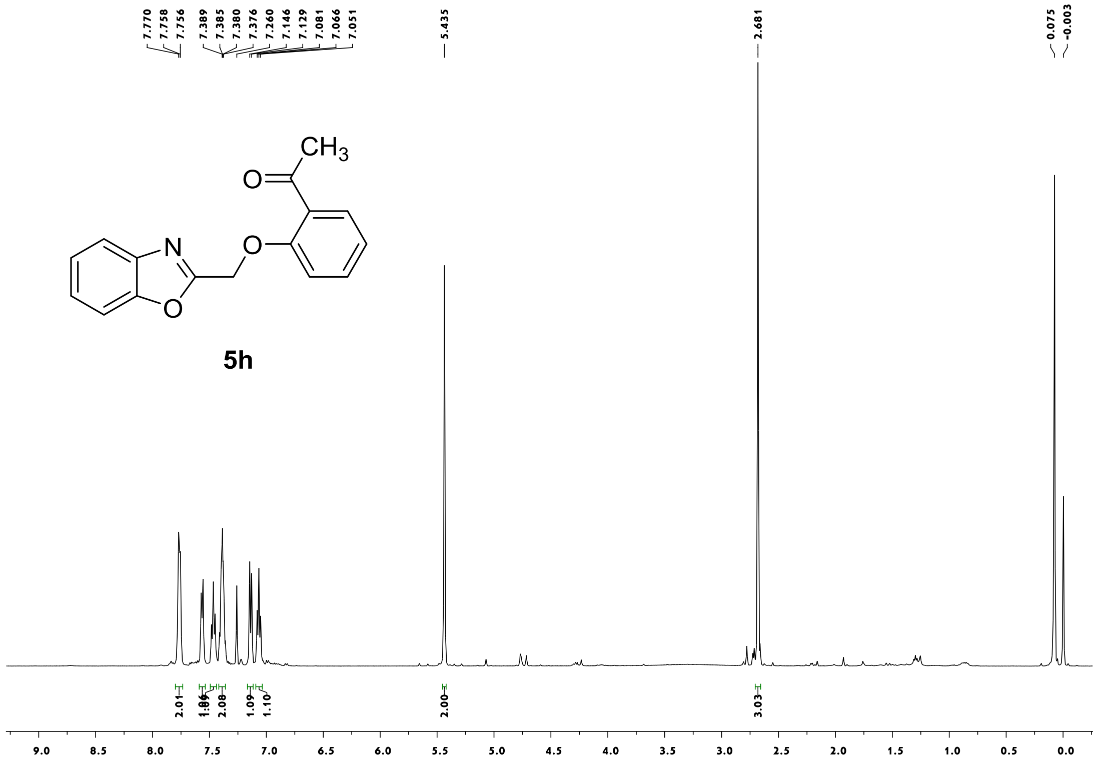
7.770
7.758
7.756
7.389
7.385
7.380
7.376
7.260
7.146
7.129
7.081
7.066
7.051
5.435
2.681
0.075
-0.003
CH3
O
N
O
O
5h
2.01
2.08
1.09
1.10
2.00
3.03
9.0 8.5 8.0 7.5 7.0 6.5 6.0 5.5 5.0 4.5 4.0 3.5 3.0 2.5 2.0 1.5 1.0 0.5 0.0

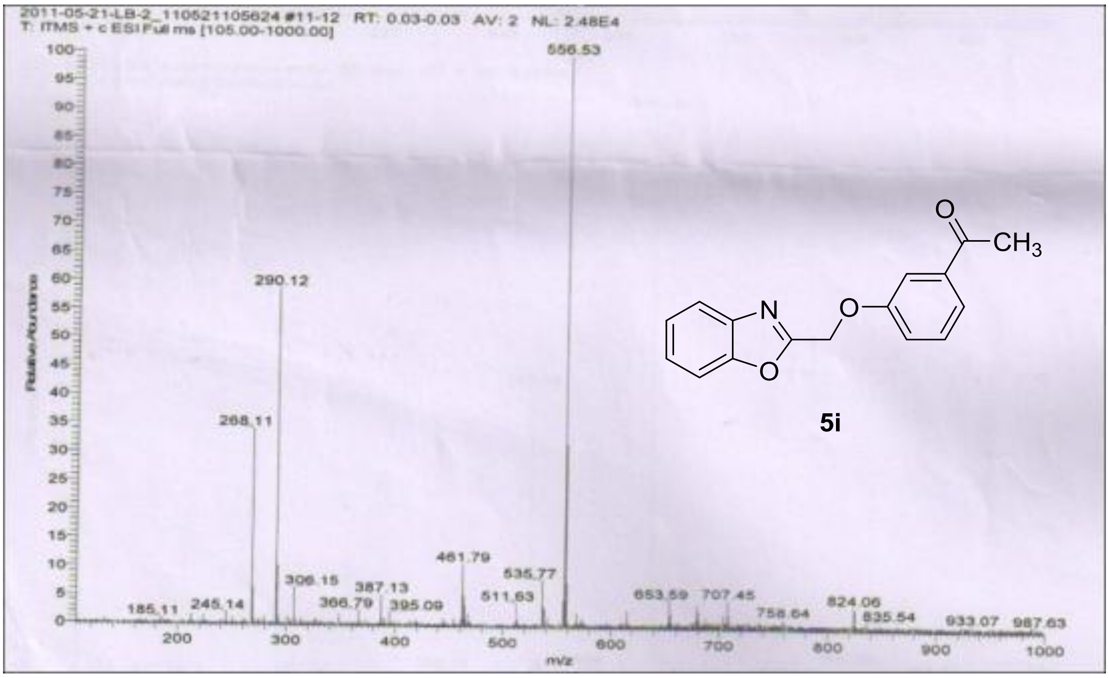

2011-05-21-LB-2_110521105624 #11-12 RT: 0.03-0.03 AV: 2 NL: 2.48E4
T: ITMS + c ESI Full ms [105.00-1000.00]
556.53
290.12
268.11
461.79
535.77
306.15
387.13
366.79
395.09
511.63
185.11
245.14
653.59
707.45
758.64
824.06
835.54
933.07
987.63
Relative Abundance
m/z
CH3
N
O
O
O
5i

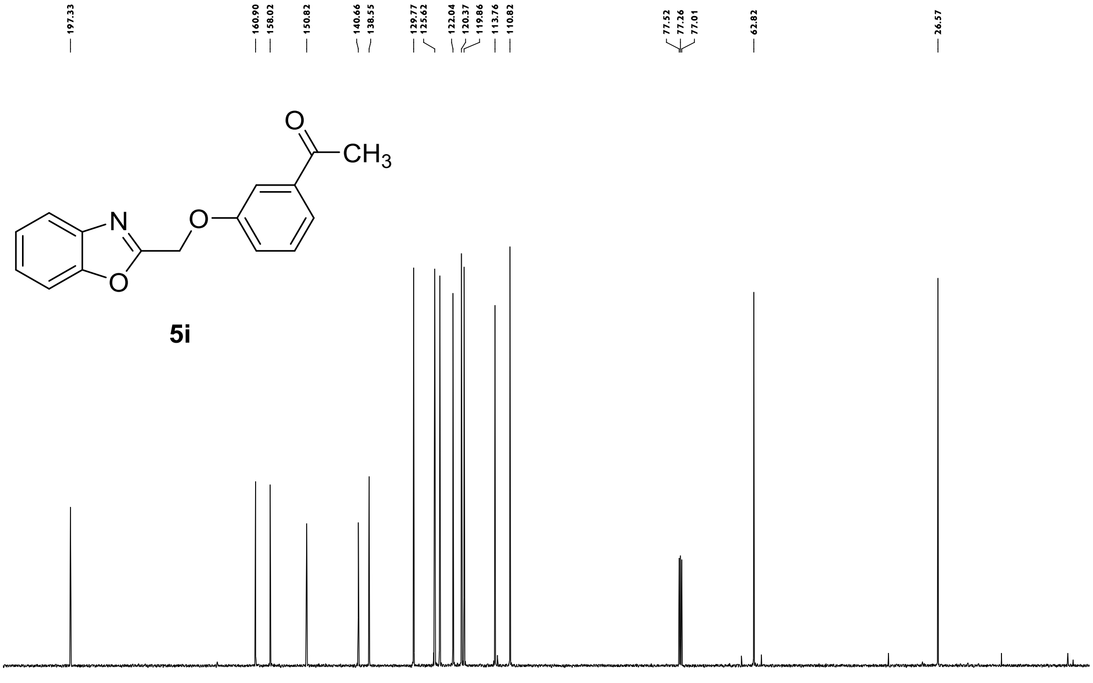

197.33
160.90
158.02
150.82
140.66
138.55
129.77
125.62
122.04
120.37
119.86
113.76
110.82
77.52
77.26
77.01
62.82
26.57
CH3
N
O
O
O
5i

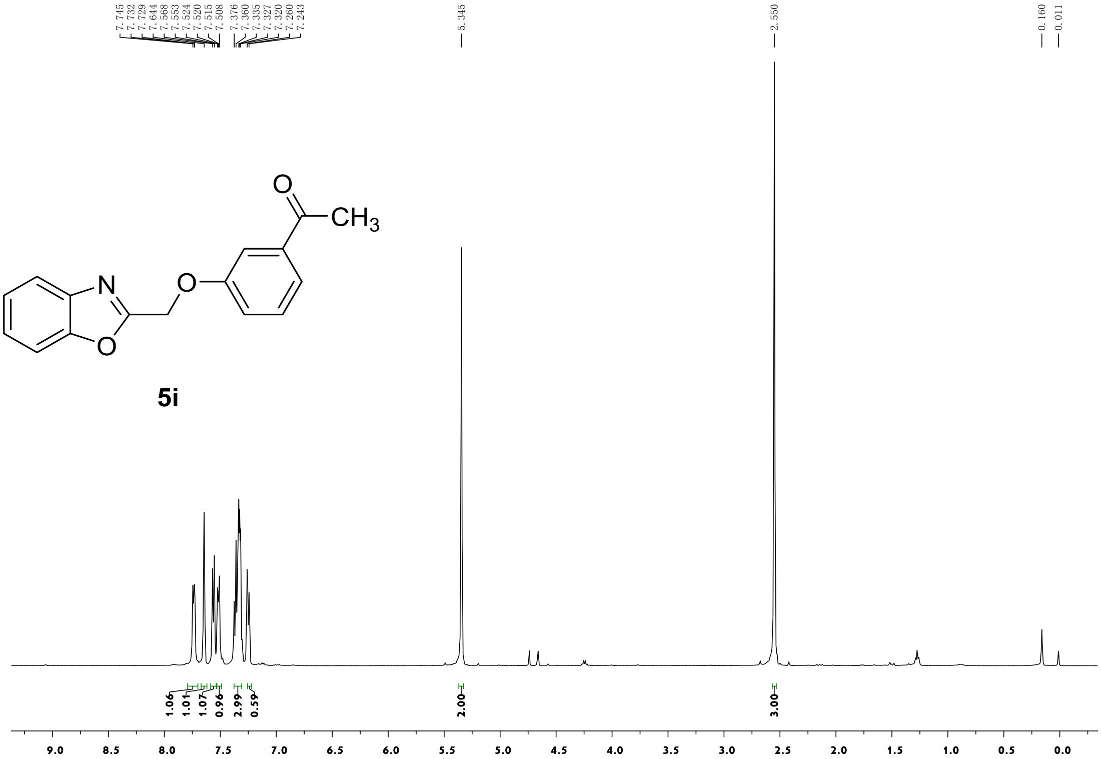

7.745
7.732
7.729
7.641
7.568
7.553
7.524
7.520
7.515
7.508
7.376
7.360
7.335
7.327
7.320
7.260
7.243
5.345
2.550
0.160
0.011
CH3
N
O
O
O
5i
1.06
1.01
1.07
0.96
2.99
0.59
2.00
3.00
9.0
8.5
8.0
7.5
7.0
6.5
6.0
5.5
5.0
4.5
4.0
3.5
3.0
2.5
2.0
1.5
1.0
0.5
0.0

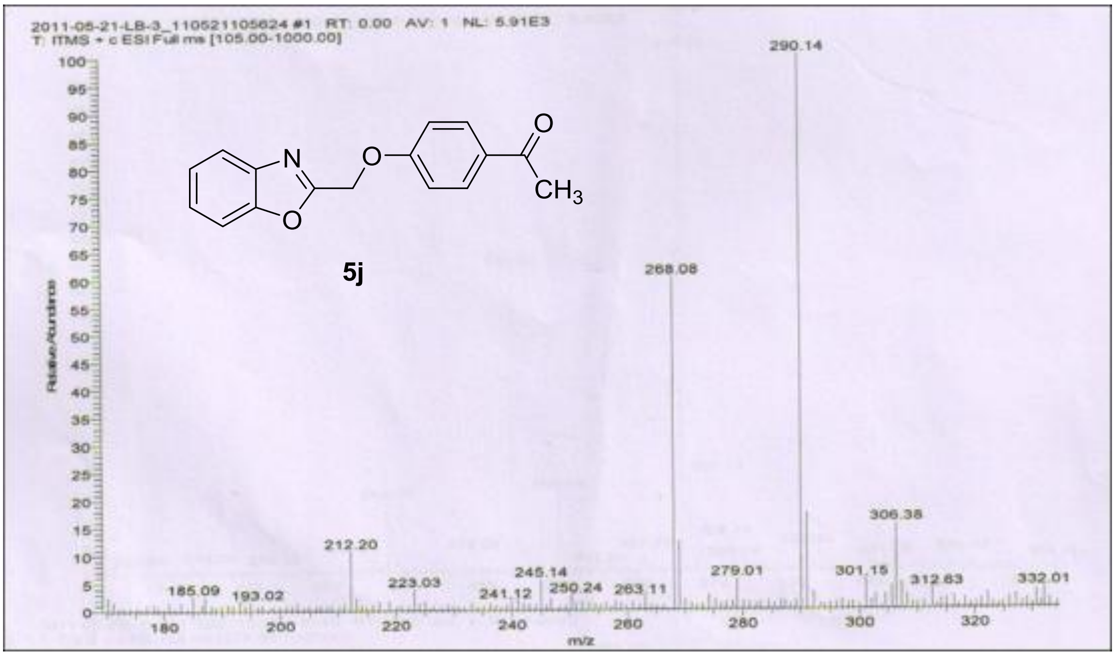

2011-05-21-LB-3_110521105624 #1 RT: 0.00 AV: 1 NL: 5.91E3
T: ITMS + c ESI Full ms [105.00-1000.00]
Relative Abundance
m/z
290.14
268.08
306.38
212.20
245.14
279.01
301.15
185.09
193.02
223.03
241.12
250.24
263.11
312.63
332.01
N
O
O
O
CH3
5j

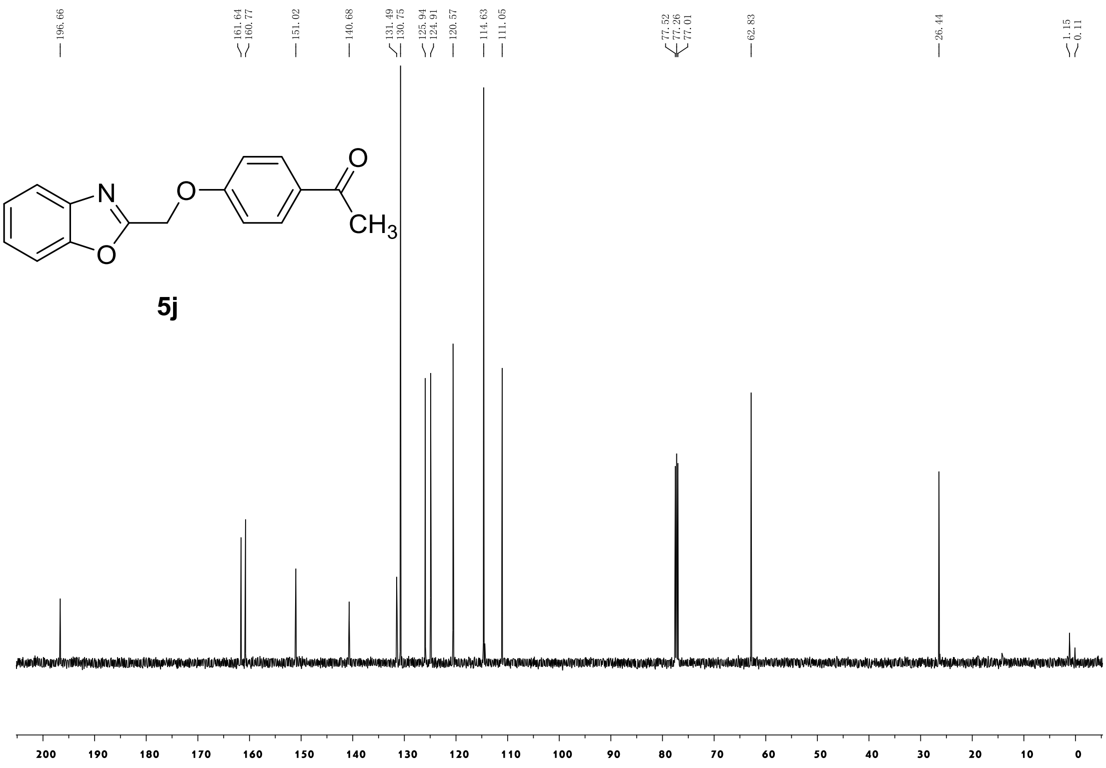
196.66
161.64
160.77
151.02
140.68
131.49
130.75
125.94
124.91
120.57
114.63
111.05
77.52
77.26
77.01
62.83
26.44
1.15
0.11
N
O
O
O
CH3
5j
200 190 180 170 160 150 140 130 120 110 100 90 80 70 60 50 40 30 20 10 0

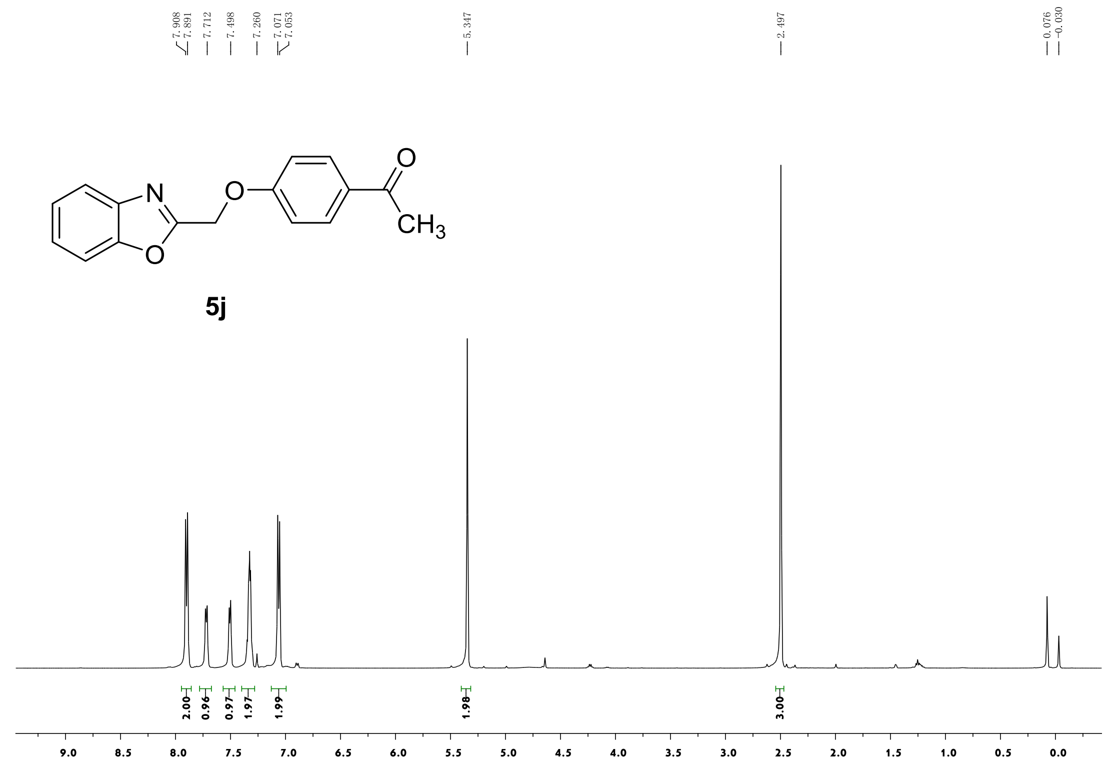
7.908
7.891
7.712
7.498
7.260
7.071
7.053
5.347
2.497
0.076
-0.030
N
O
O
O
CH3
5j
2.00
0.96
0.97
1.97
1.99
1.98
3.00
9.0 8.5 8.0 7.5 7.0 6.5 6.0 5.5 5.0 4.5 4.0 3.5 3.0 2.5 2.0 1.5 1.0 0.5 0.0

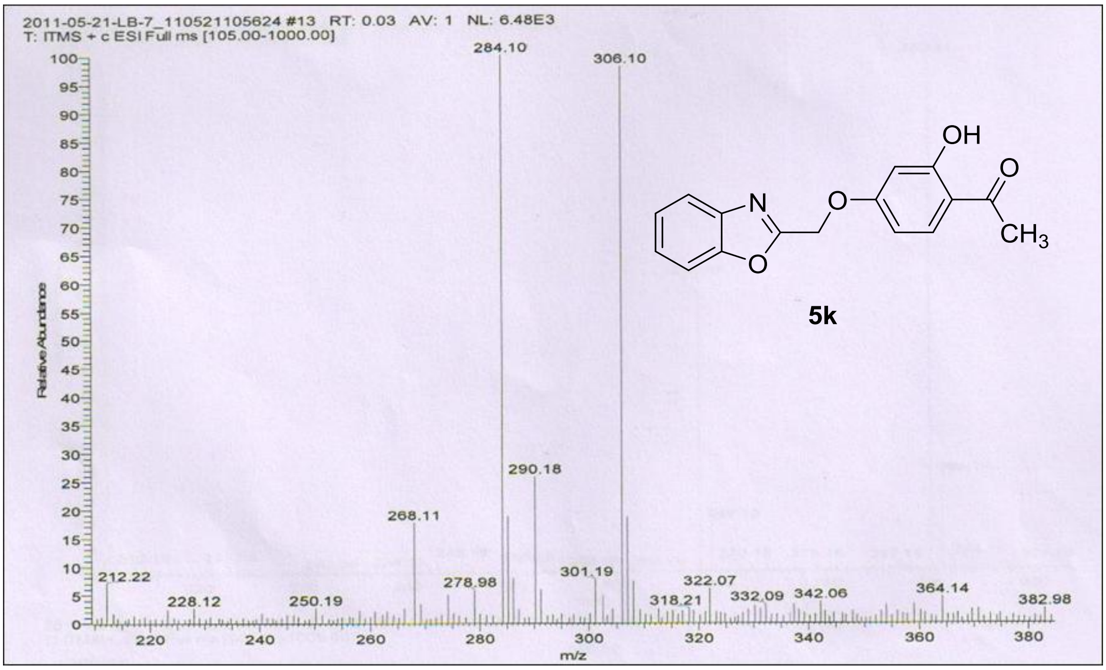
2011-05-21-LB-7_110521105624 #13 RT: 0.03 AV: 1 NL: 6.48E3
T: ITMS + c ESI Full ms [105.00-1000.00]
284.10
306.10
290.18
268.11
212.22
228.12
250.19
278.98
301.19
318.21
322.07
332.09
342.06
364.14
382.98
Relative Abundance
m/z
OH
O
N
O
CH3
5k

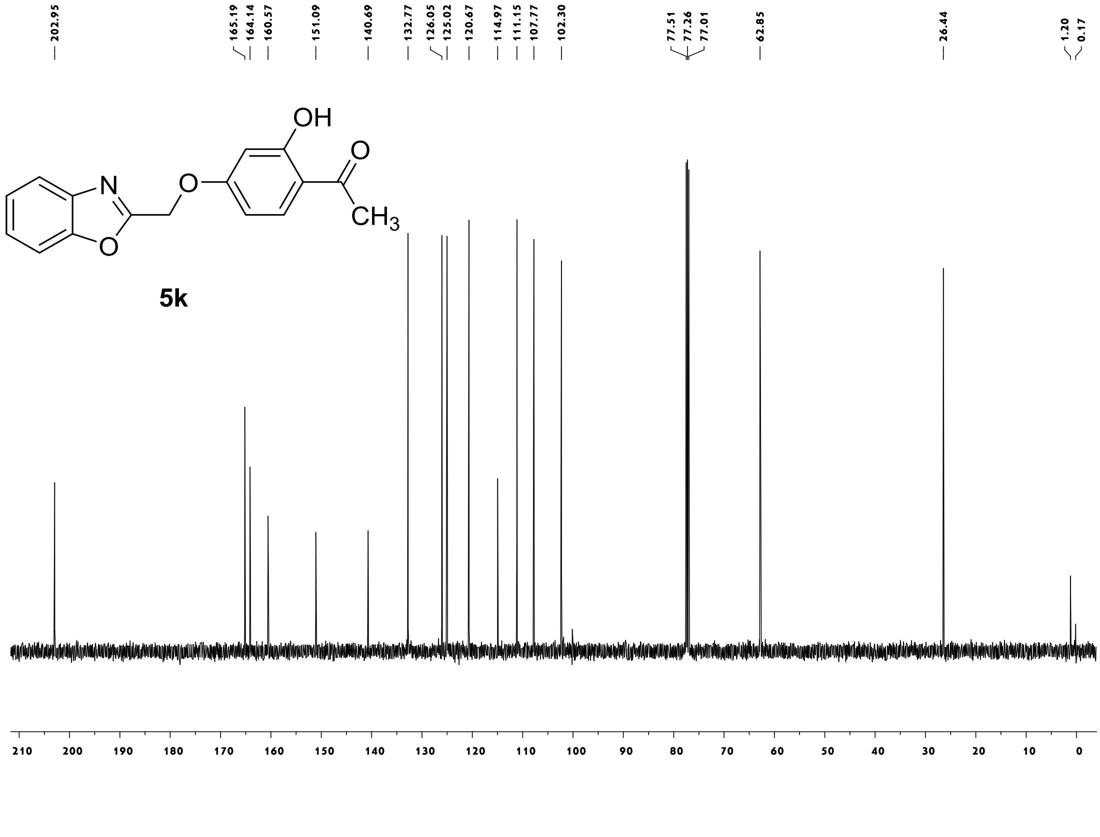
202.95
165.19
164.14
160.57
151.09
140.69
132.77
126.05
125.02
120.67
114.97
111.15
107.77
102.30
77.51
77.26
77.01
62.85
26.44
1.20
0.17
OH
O
N
O
CH3
5k

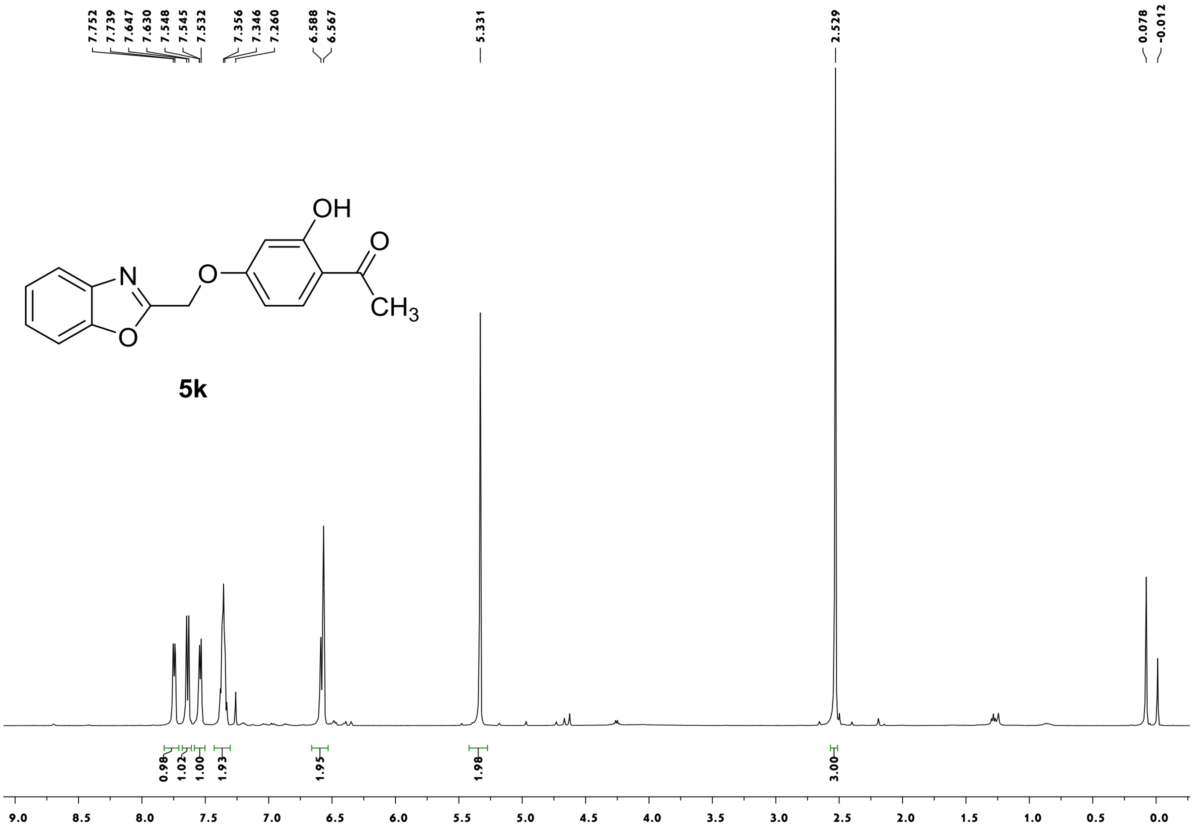
7.752
7.739
7.647
7.630
7.548
7.545
7.532
7.356
7.346
7.260
6.588
6.567
5.331
2.529
0.078
-0.012
OH
O
N
O
O
CH3
5k
0.98
1.02
1.00
1.93
1.95
1.98
3.00
9.0
8.5
8.0
7.5
7.0
6.5
6.0
5.5
5.0
4.5
4.0
3.5
3.0
2.5
2.0
1.5
1.0
0.5
0.0

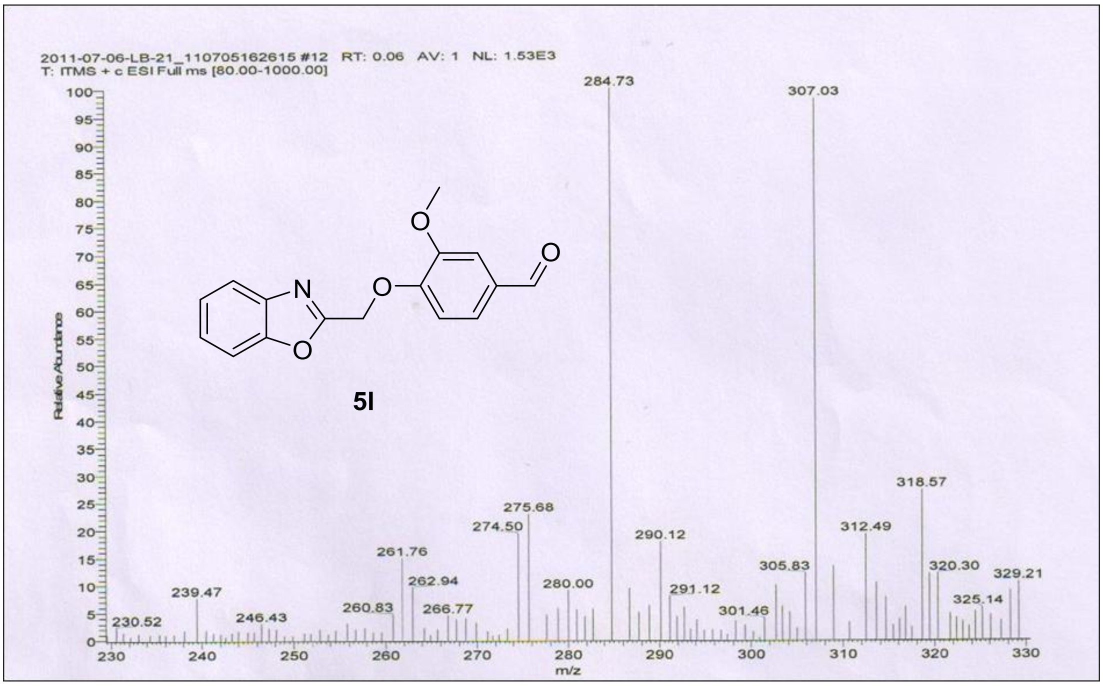
2011-07-06-LB-21_110705162615 #12 RT: 0.06 AV: 1 NL: 1.53E3
T: ITMS + c ESI Full ms [80.00-1000.00]
Relative Abundance
m/z
5l
284.73
307.03
318.57
275.68
274.50
312.49
290.12
261.76
305.83
320.30
329.21
262.94
280.00
239.47
291.12
325.14
266.77
260.83
301.46
246.43
230.52

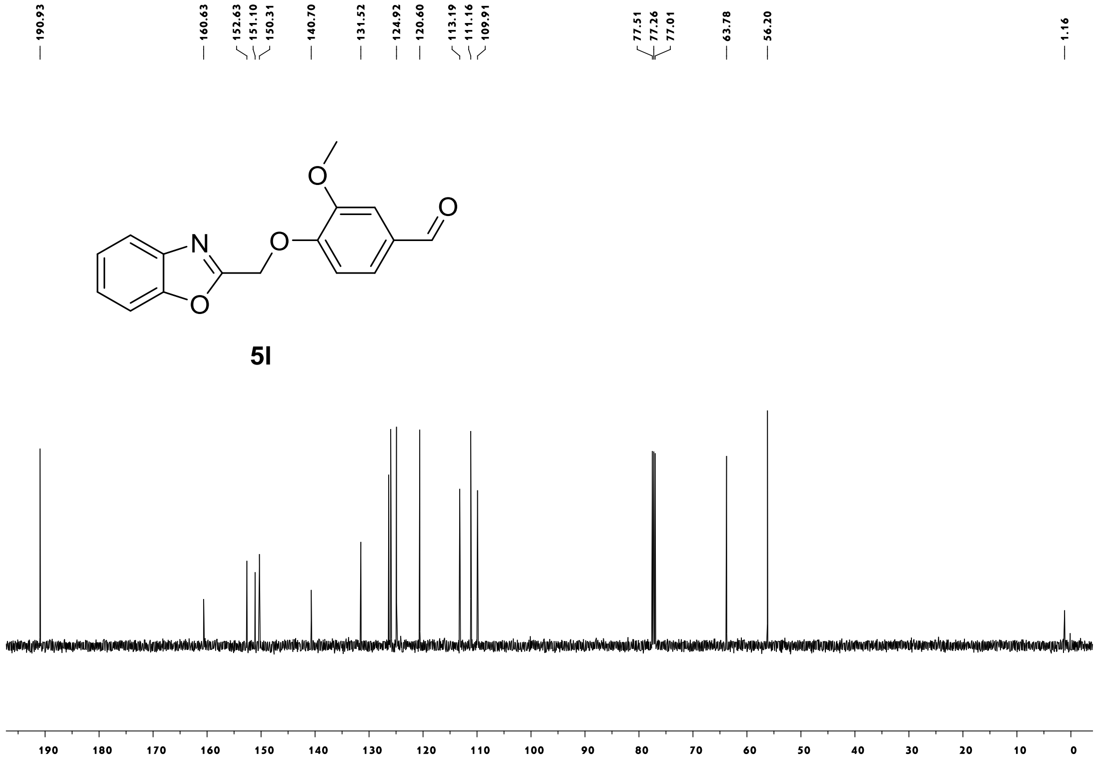
190.93
160.63
152.63
151.10
150.31
140.70
131.52
124.92
120.60
113.19
111.16
109.91
77.51
77.26
77.01
63.78
56.20
1.16
N
O
O
O
O
5l
190
180
170
160
150
140
130
120
110
100
90
80
70
60
50
40
30
20
10
0

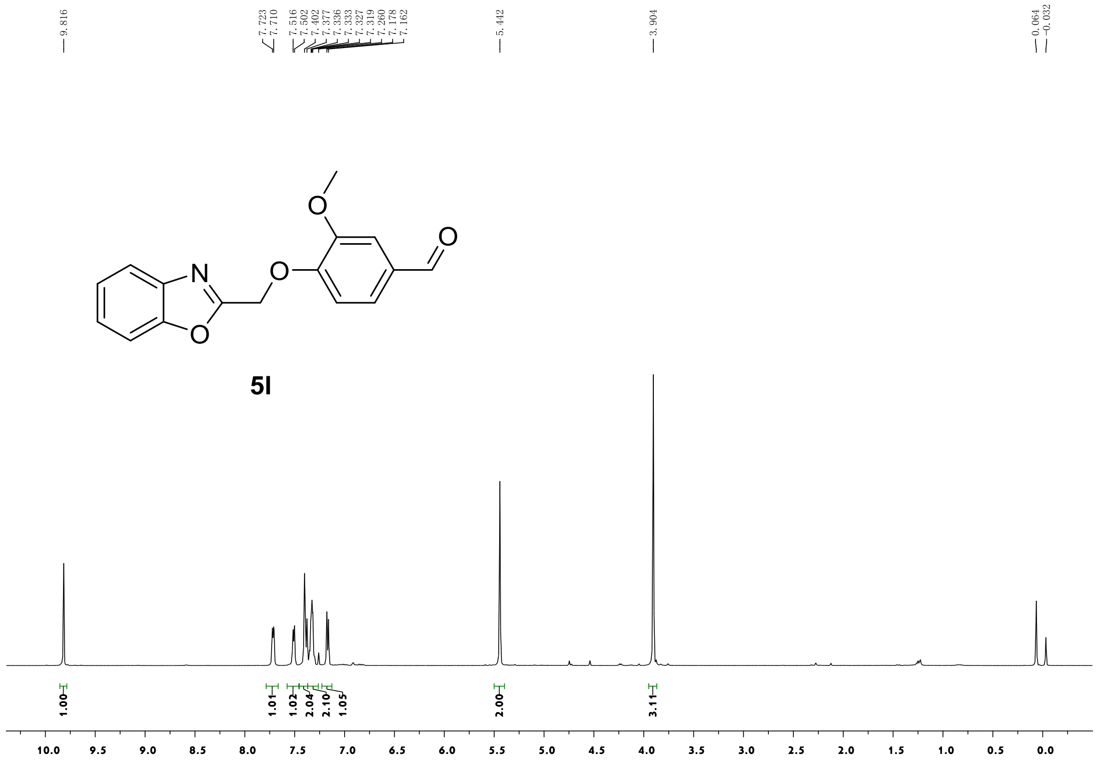
9.816
7.723
7.710
7.516
7.502
7.402
7.377
7.336
7.333
7.327
7.319
7.260
7.178
7.162
5.442
3.904
0.064
-0.032
N
O
O
O
O
5l
1.00
1.01
1.02
2.04
2.10
1.05
2.00
3.11
10.0
9.5
9.0
8.5
8.0
7.5
7.0
6.5
6.0
5.5
5.0
4.5
4.0
3.5
3.0
2.5
2.0
1.5
1.0
0.5
0.0

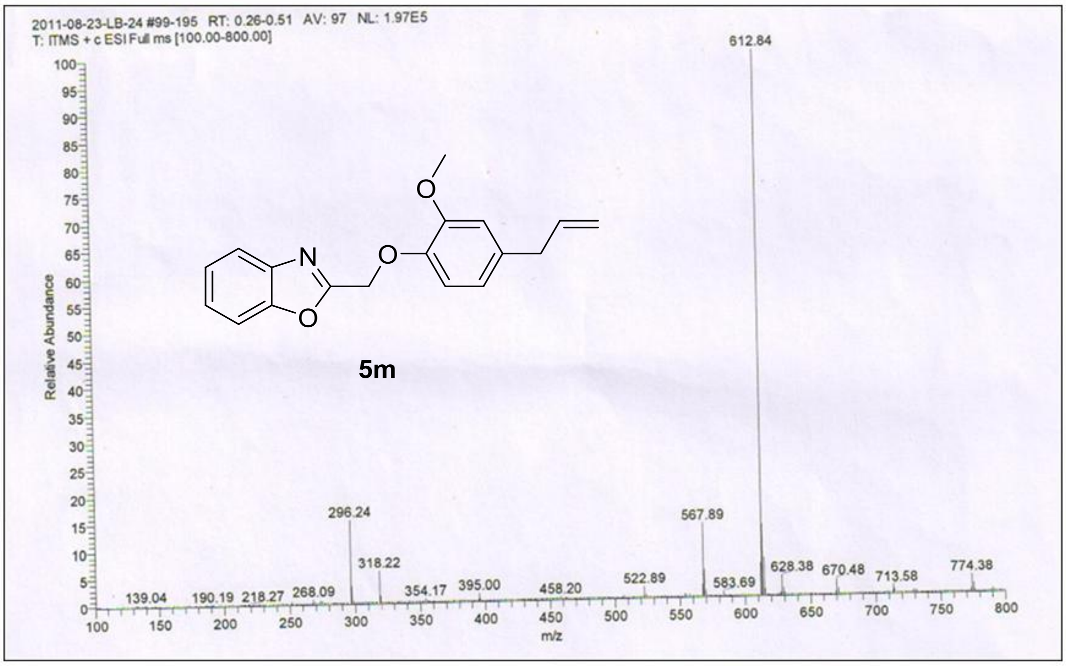
2011-08-23-LB-24 #99-195 RT: 0.26-0.51 AV: 97 NL: 1.97E5
T: ITMS + c ESI Full ms [100.00-800.00]
Relative Abundance
m/z
612.84
296.24
318.22
567.89
139.04
190.19
218.27
268.09
354.17
395.00
458.20
522.89
583.69
628.38
670.48
713.58
774.38
N
O
O
O
5m

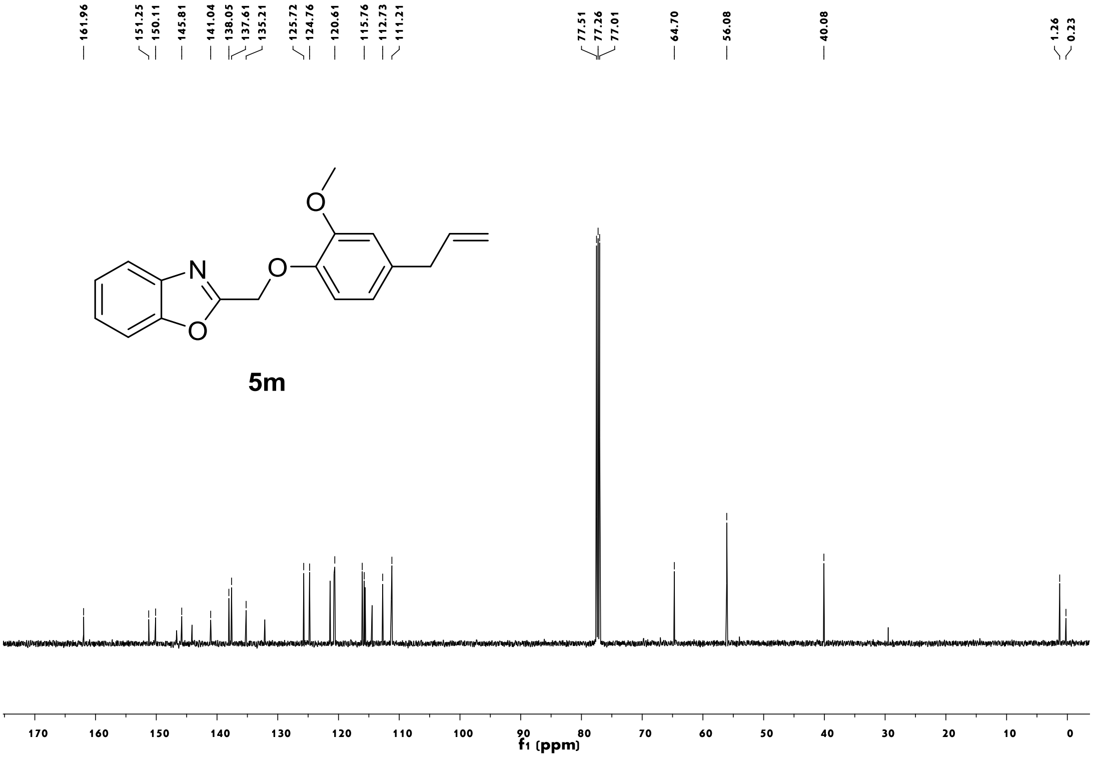
161.96
151.25
150.11
145.81
141.04
138.05
137.61
135.21
125.72
124.76
120.61
115.76
112.73
111.21
77.51
77.26
77.01
64.70
56.08
40.08
1.26
0.23
N
O
O
O
5m
f1 (ppm)

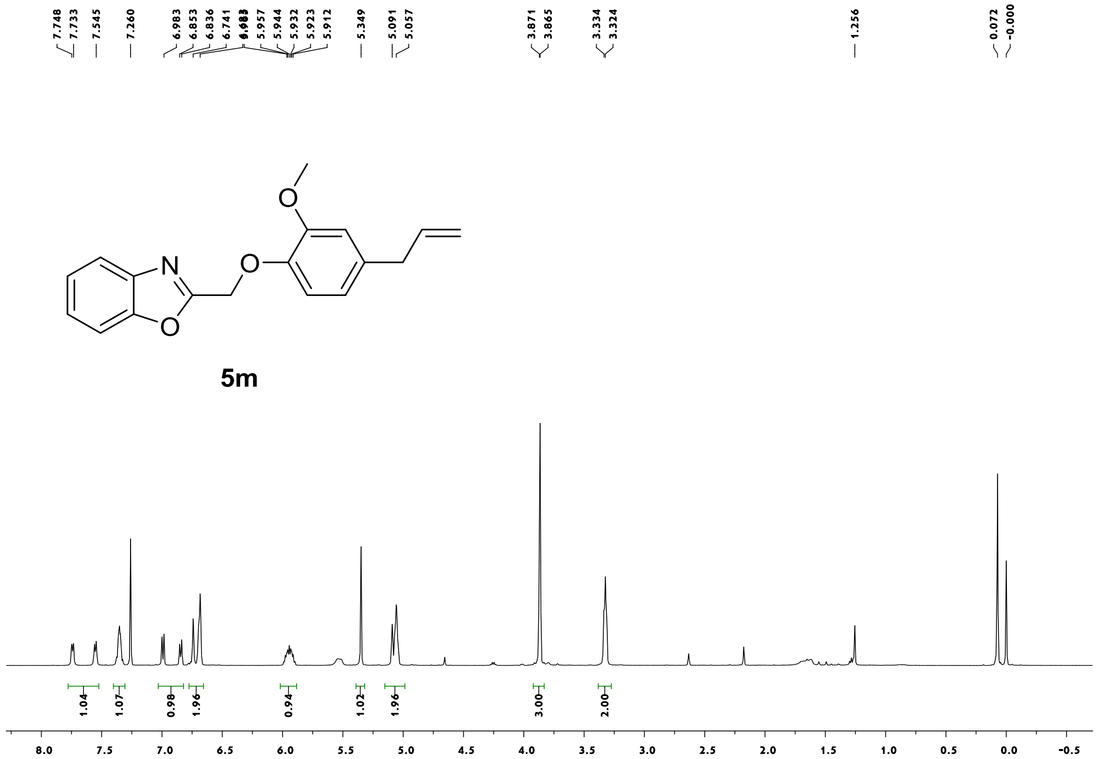

7.748
7.733
7.545
7.260
6.983
6.853
6.836
6.741
5.957
5.944
5.932
5.923
5.912
5.349
5.091
5.057
3.871
3.865
3.334
3.324
1.256
0.072
-0.000
N
O
O
O
5m
1.04
1.07
0.98
1.96
0.94
1.02
1.96
3.00
2.00
8.0
7.5
7.0
6.5
6.0
5.5
5.0
4.5
4.0
3.5
3.0
2.5
2.0
1.5
1.0
0.5
0.0
-0.5

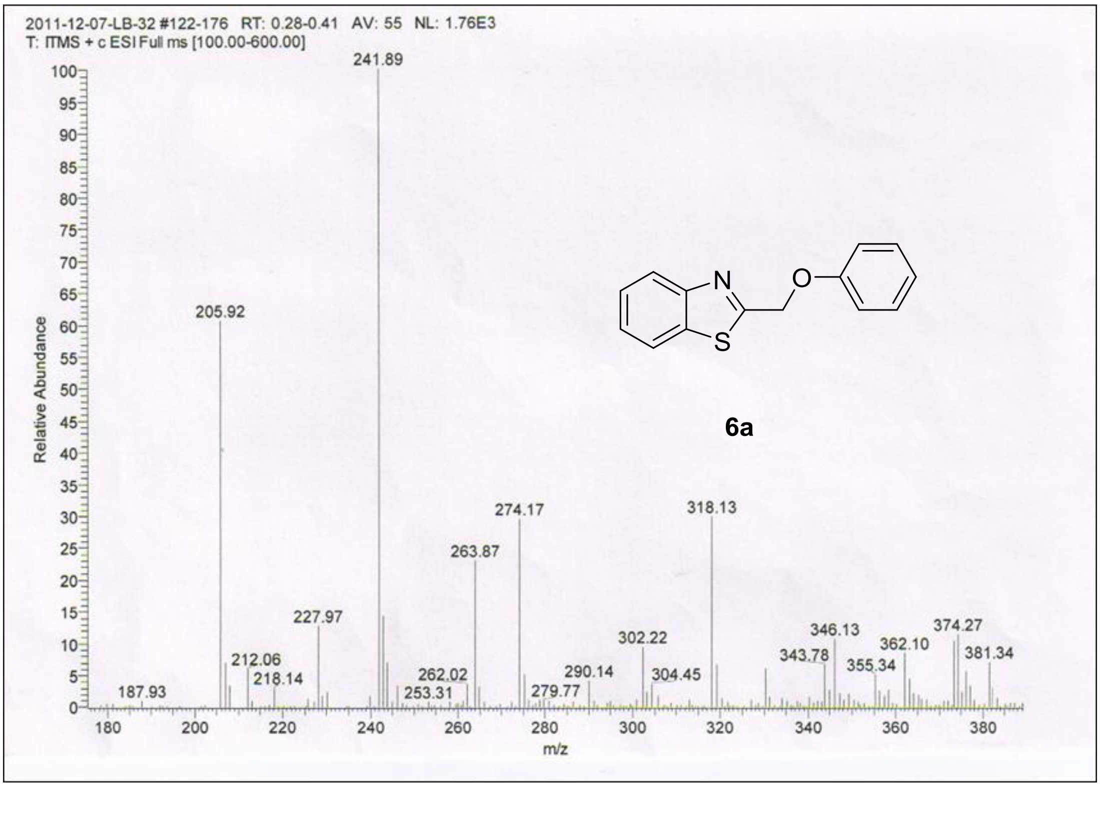

2011-12-07-LB-32 #122-176 RT: 0.28-0.41 AV: 55 NL: 1.76E3
T: ITMS + c ESI Full ms [100.00-600.00]
Relative Abundance
m/z
241.89
205.92
274.17
318.13
263.87
227.97
212.06
218.14
187.93
262.02
253.31
279.77
290.14
302.22
304.45
343.78
346.13
355.34
362.10
374.27
381.34
N
O
S
6a

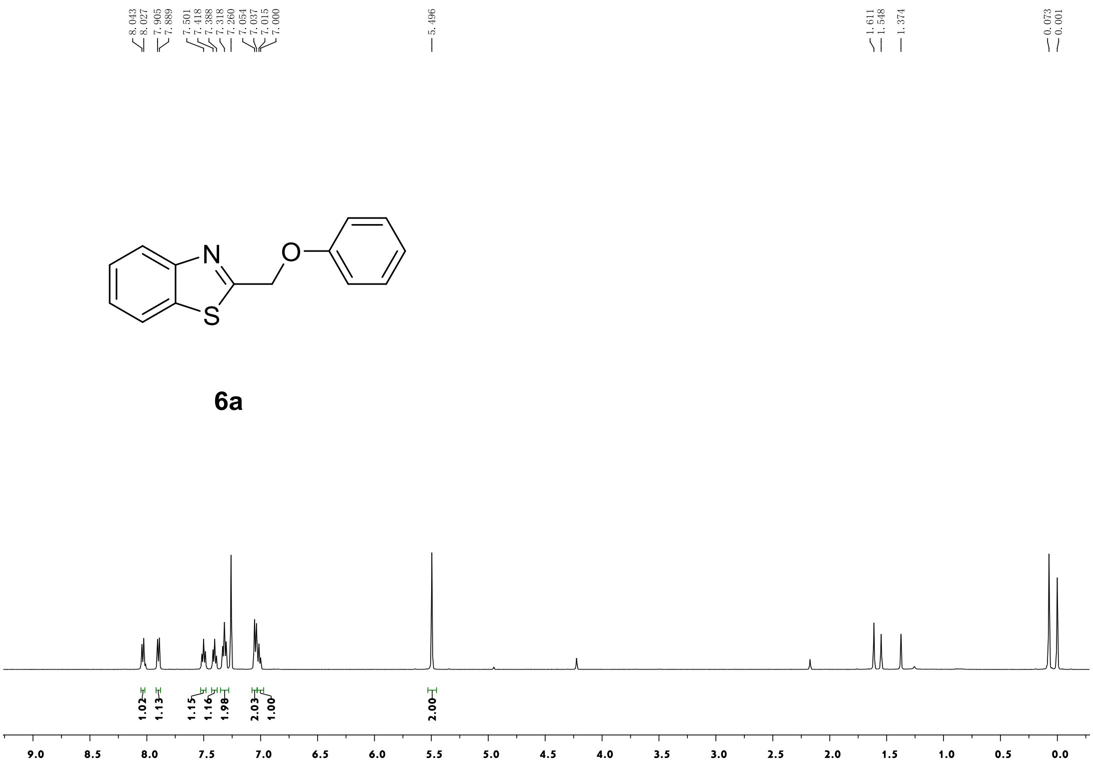
8.043
8.027
7.905
7.889
7.501
7.418
7.388
7.318
7.260
7.054
7.037
7.015
7.000
5.496
1.611
1.548
1.374
0.073
0.001
N
O
S
6a
1.02
1.13
1.15
1.16
1.98
2.03
1.00
2.00
9.0 8.5 8.0 7.5 7.0 6.5 6.0 5.5 5.0 4.5 4.0 3.5 3.0 2.5 2.0 1.5 1.0 0.5 0.0

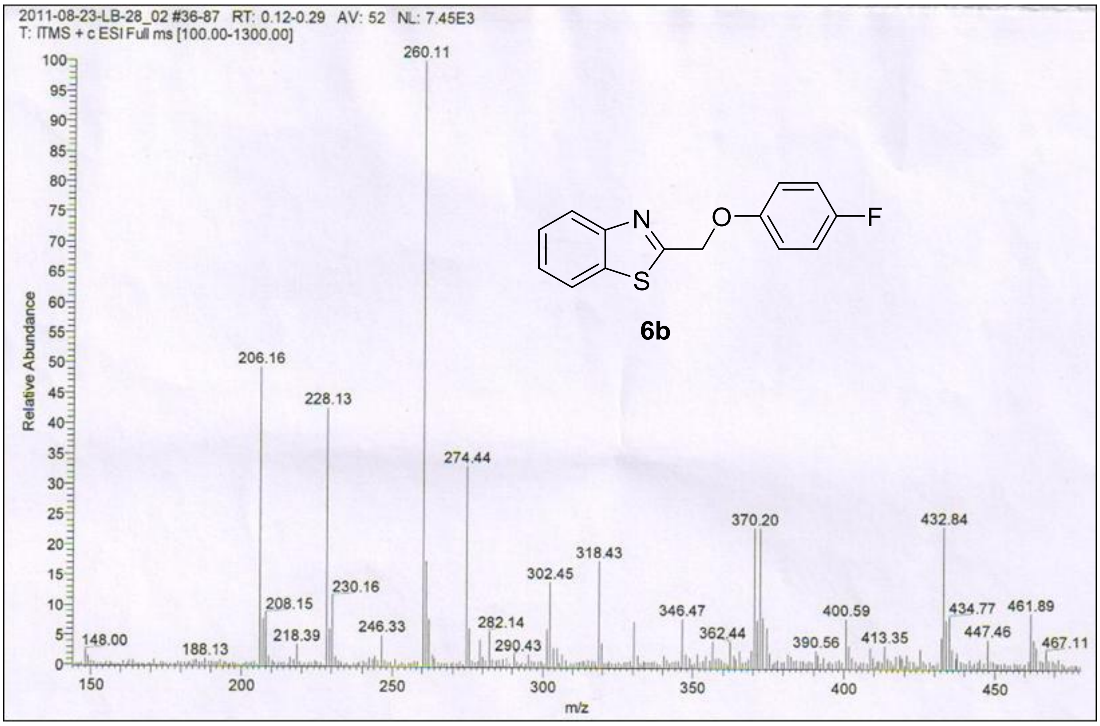
2011-08-23-LB-28_02 #36-87 RT: 0.12-0.29 AV: 52 NL: 7.45E3
T: ITMS + c ESI Full ms [100.00-1300.00]
Relative Abundance
100 95 90 85 80 75 70 65 60 55 50 45 40 35 30 25 20 15 10 5 0
260.11
206.16
228.13
274.44
370.20
432.84
318.43
302.45
230.16
208.15
400.59
434.77
461.89
346.47
246.33
282.14
218.39
362.44
447.46
148.00
188.13
290.43
390.56
413.35
467.11
150 200 250 300 350 400 450
m/z
N
O
F
S
6b

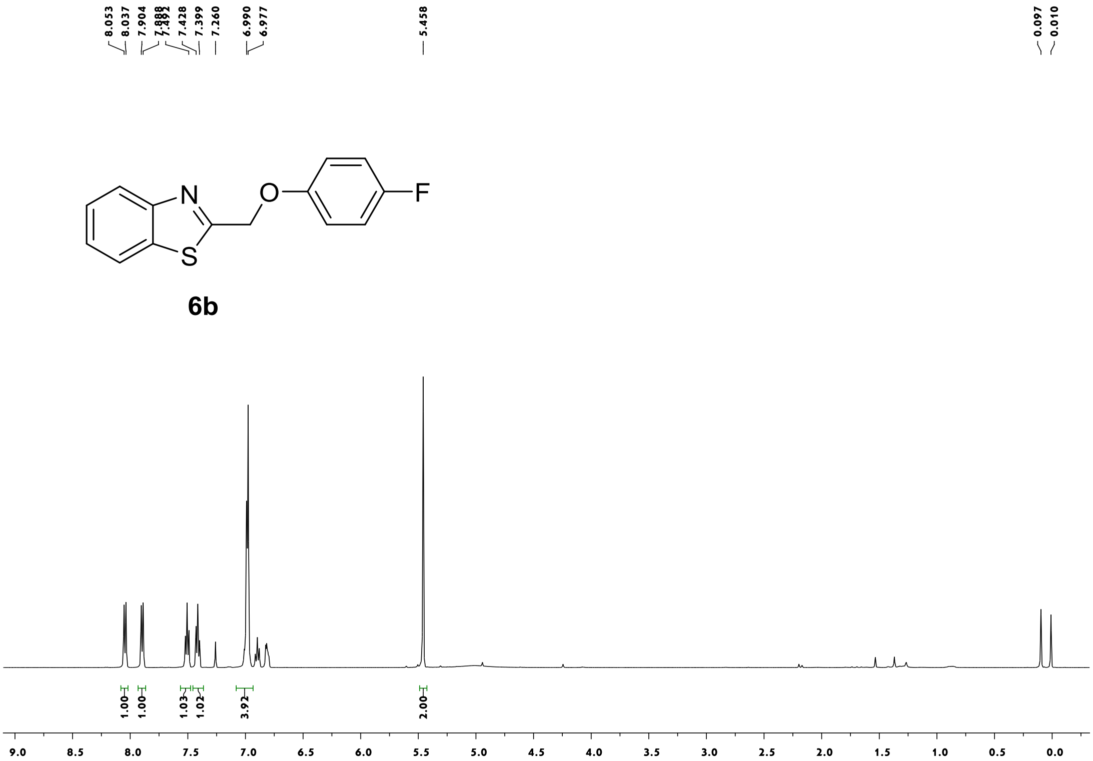
8.053
8.037
7.904
7.428
7.399
7.260
6.990
6.977
5.458
0.097
0.010
N
O
F
S
6b
1.00
1.00
1.03
1.02
3.92
2.00
9.0 8.5 8.0 7.5 7.0 6.5 6.0 5.5 5.0 4.5 4.0 3.5 3.0 2.5 2.0 1.5 1.0 0.5 0.0

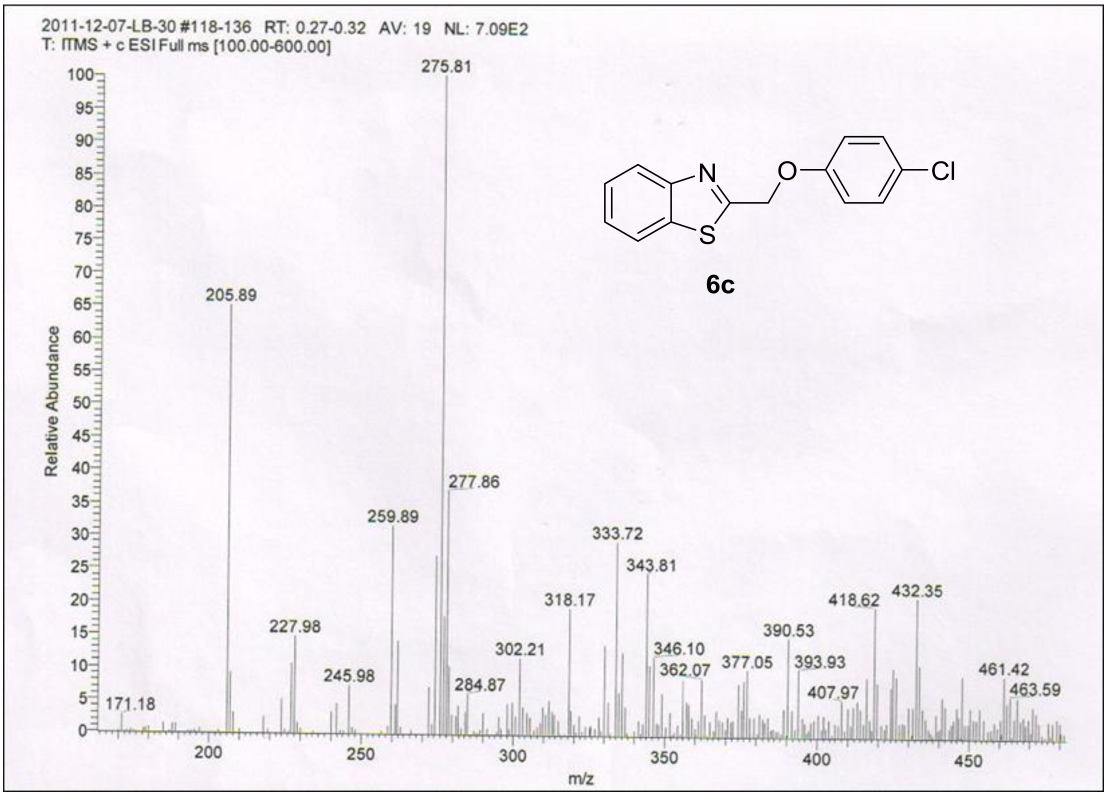
2011-12-07-LB-30 #118-136 RT: 0.27-0.32 AV: 19 NL: 7.09E2
T: ITMS + c ESI Full ms [100.00-600.00]
Relative Abundance
m/z
275.81
205.89
277.86
259.89
333.72
343.81
318.17
418.62
432.35
227.98
390.53
302.21
346.10
362.07
377.05
393.93
245.98
284.87
461.42
463.59
407.97
171.18
N
O
Cl
S
6c

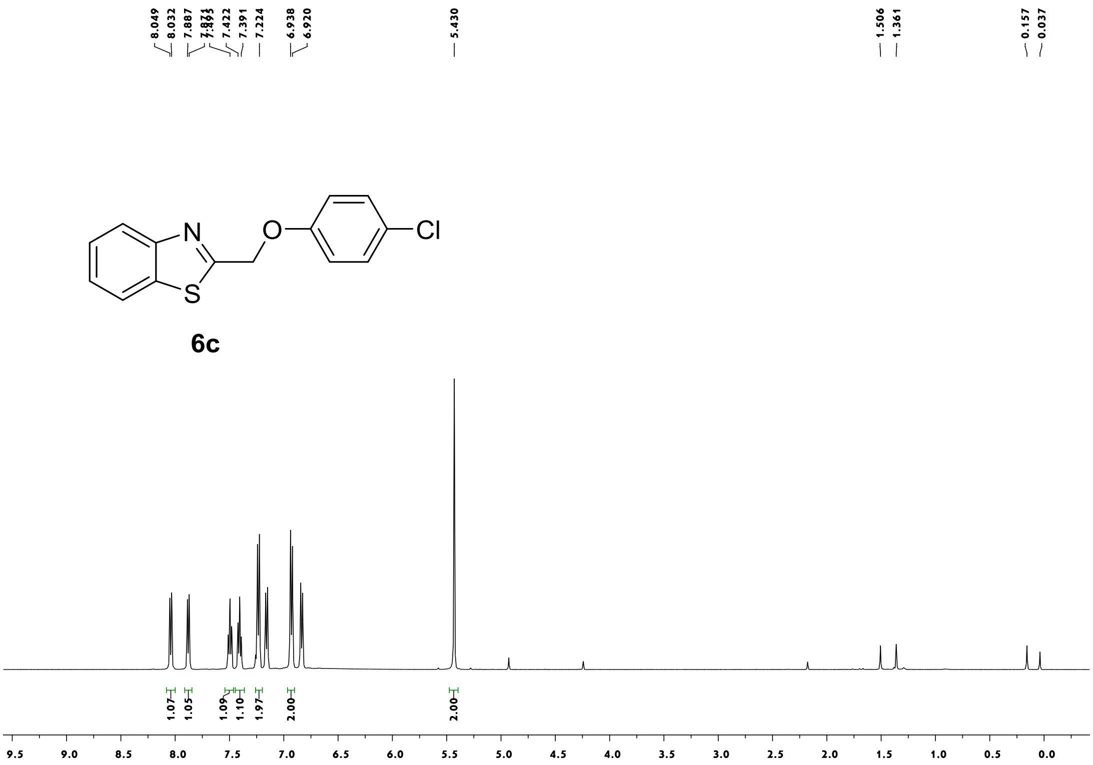
8.049
8.032
7.887
7.871
7.505
7.422
7.391
7.224
6.938
6.920
5.430
1.506
1.361
0.157
0.037
N
O
Cl
S
6c
1.07
1.05
1.09
1.10
1.97
2.00
2.00
9.5 9.0 8.5 8.0 7.5 7.0 6.5 6.0 5.5 5.0 4.5 4.0 3.5 3.0 2.5 2.0 1.5 1.0 0.5 0.0

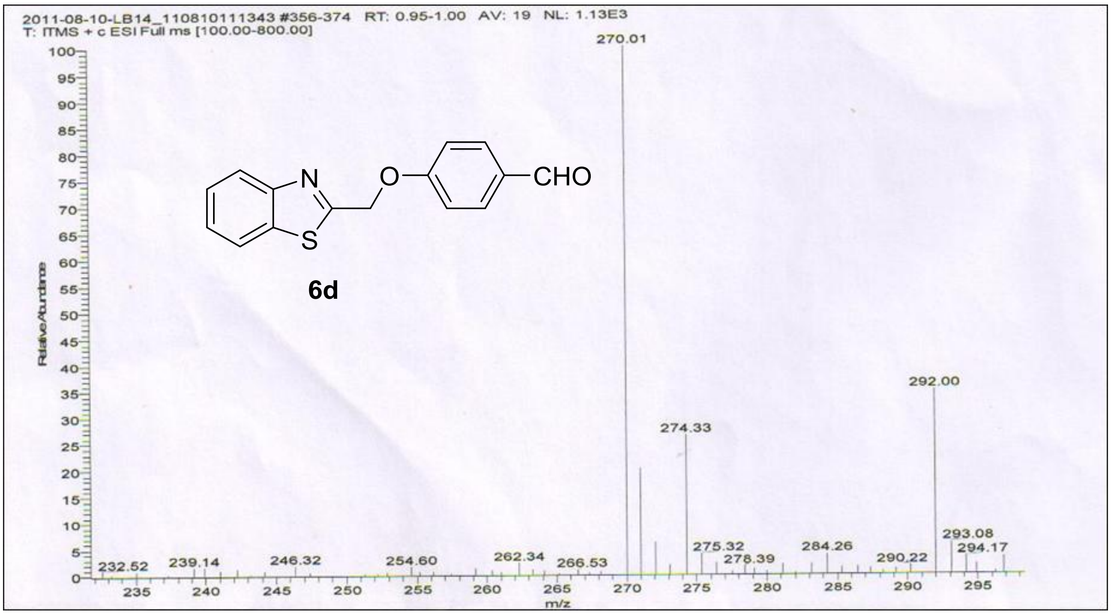
2011-08-10-LB14_110810111343 #356-374 RT: 0.95-1.00 AV: 19 NL: 1.13E3
T: ITMS + c ESI Full ms [100.00-800.00]
Relative Abundance
m/z
N
O
CHO
S
6d
270.01
292.00
274.33
275.32
278.39
284.26
290.22
293.08
294.17
232.52
239.14
246.32
254.60
262.34
266.53

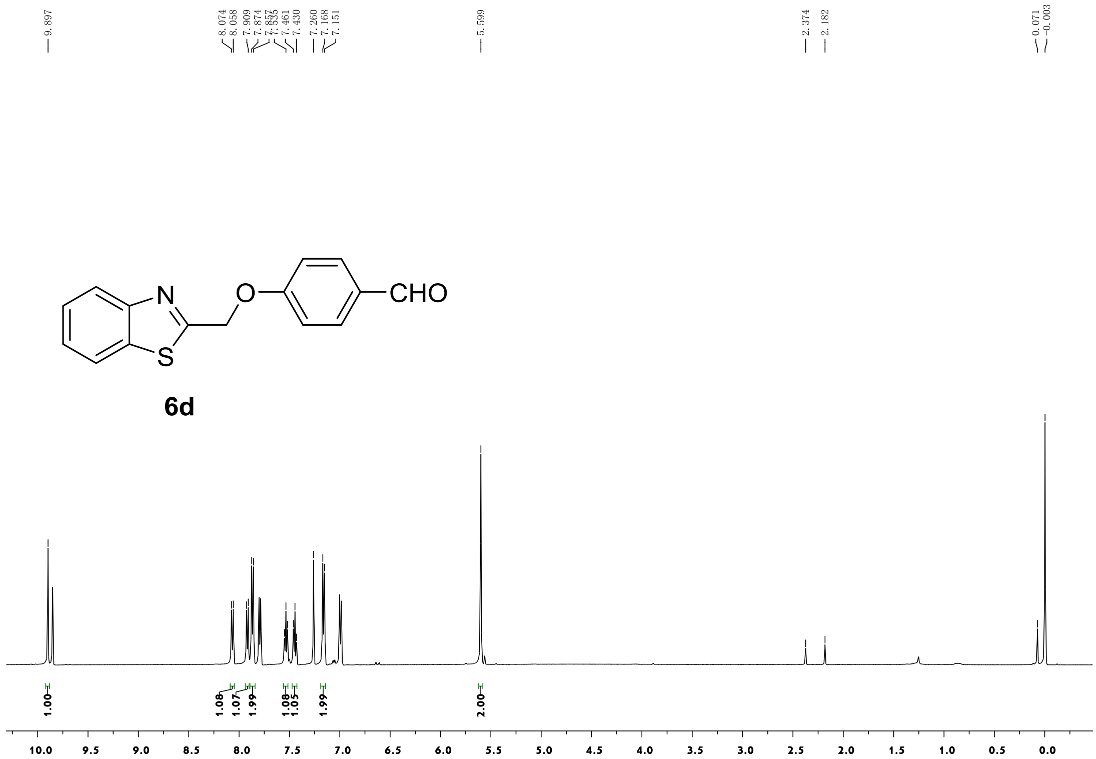

9.897
8.074
8.058
7.909
7.874
7.857
7.535
7.461
7.430
7.260
7.168
7.151
5.599
2.374
2.182
0.071
-0.003
N
O
CHO
S
1.00
1.08
1.07
1.99
1.08
1.05
1.99
2.00
10.0
9.5
9.0
8.5
8.0
7.5
7.0
6.5
6.0
5.5
5.0
4.5
4.0
3.5
3.0
2.5
2.0
1.5
1.0
0.5
0.0

6d

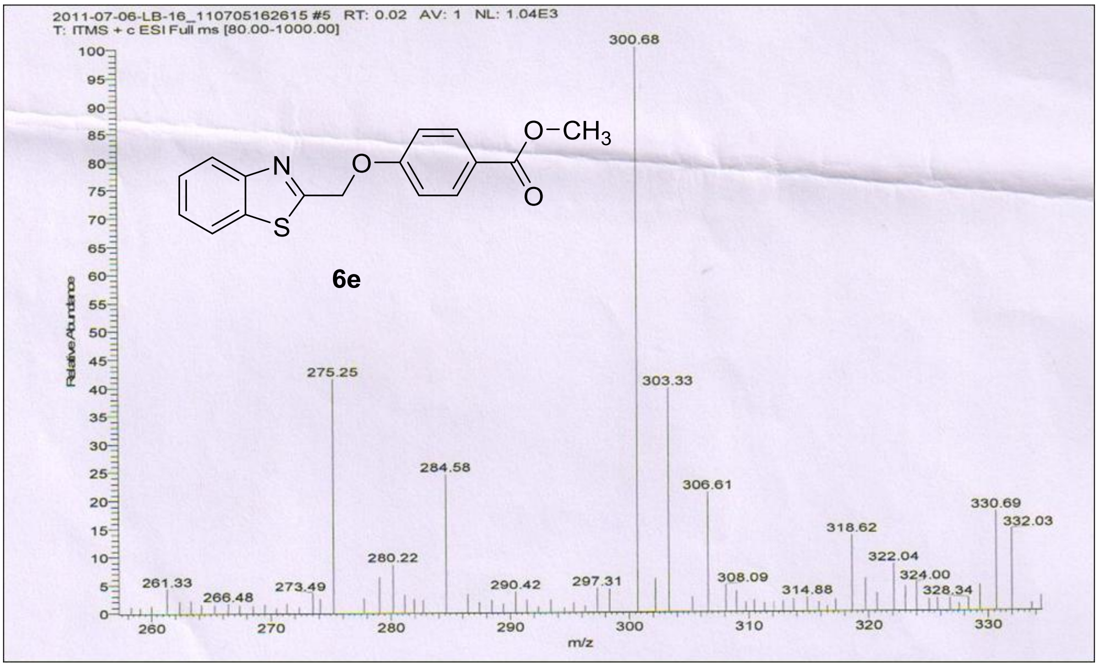

2011-07-06-LB-16_110705162615 #5 RT: 0.02 AV: 1 NL: 1.04E3
T: ITMS + c ESI Full ms [80.00-1000.00]
Relative Abundance
m/z
300.68
275.25
303.33
284.58
306.61
330.69
332.03
318.62
322.04
324.00
328.34
280.22
261.33
266.48
273.49
290.42
297.31
308.09
314.88
N
O
O−CH3
O
S

6e

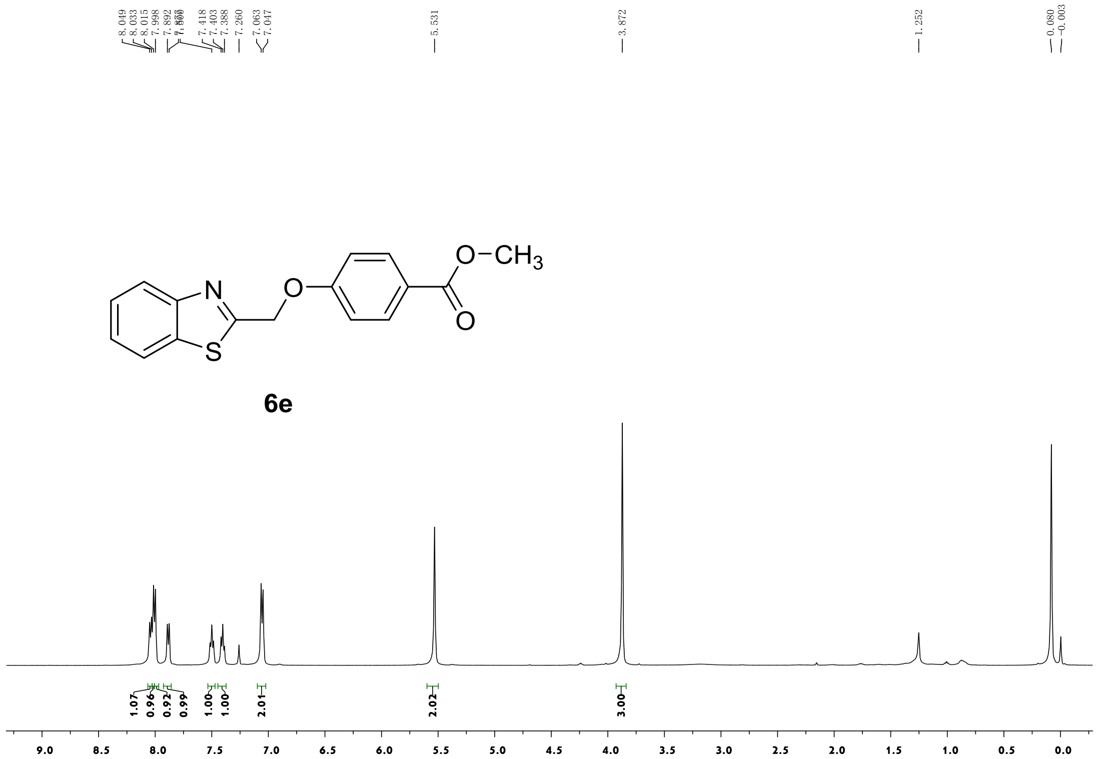
8.049
8.033
8.015
7.998
7.892
7.418
7.403
7.388
7.260
7.063
7.047
5.531
3.872
1.252
0.080
-0.003
O−CH3
N
O
S
O
6e
1.07
0.96
0.92
0.99
1.00
1.00
2.01
2.02
3.00
9.0 8.5 8.0 7.5 7.0 6.5 6.0 5.5 5.0 4.5 4.0 3.5 3.0 2.5 2.0 1.5 1.0 0.5 0.0

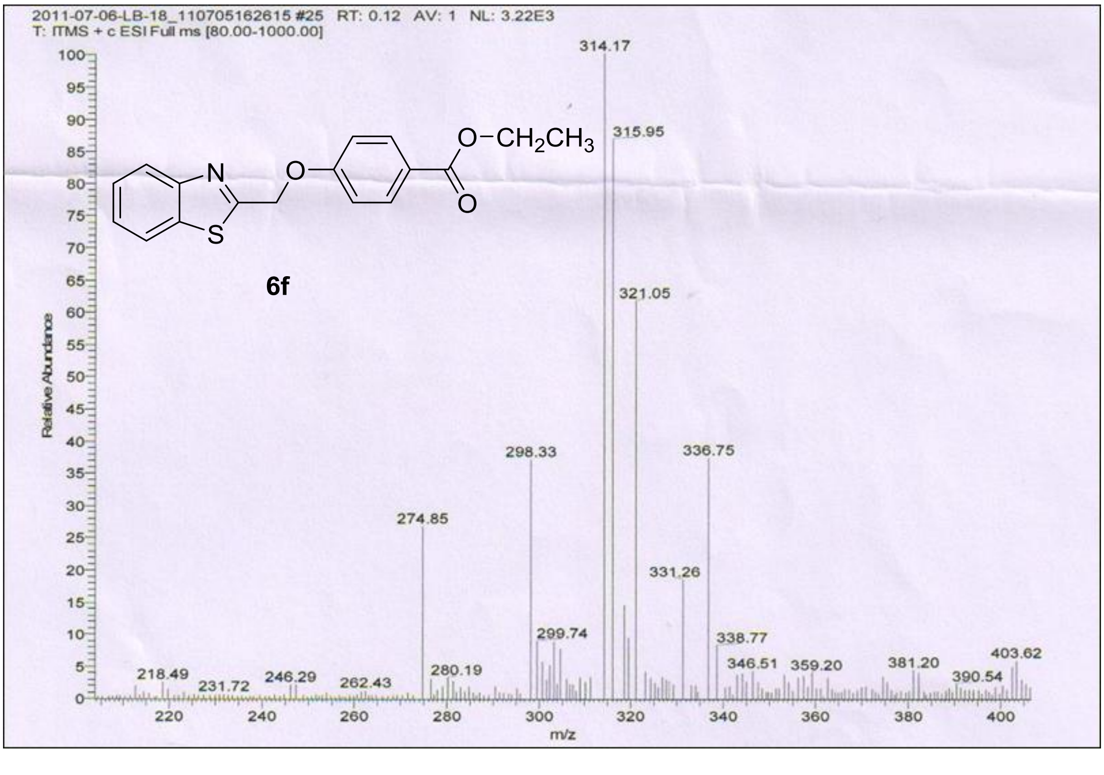
2011-07-06-LB-18_110705162615 #25 RT: 0.12 AV: 1 NL: 3.22E3
T: ITMS + c ESI Full ms [80.00-1000.00]
O−CH2CH3
N
O
S
O
6f
Relative Abundance
m/z
314.17
315.95
321.05
298.33
336.75
274.85
331.26
299.74
338.77
218.49
231.72
246.29
262.43
280.19
346.51
359.20
381.20
390.54
403.62

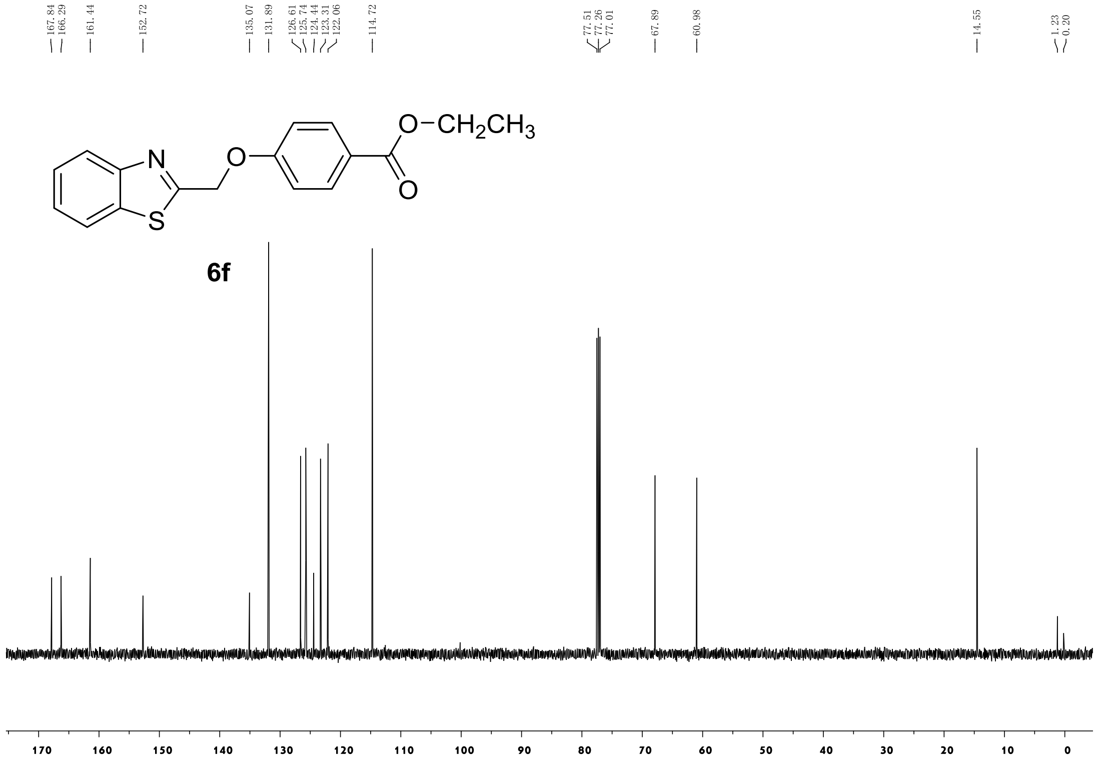
167.84
166.29
161.44
152.72
135.07
131.89
126.61
125.74
124.44
123.31
122.06
114.72
77.51
77.26
77.01
67.89
60.98
14.55
1.23
0.20
O−CH2CH3
N
O
S
O
6f
170 160 150 140 130 120 110 100 90 80 70 60 50 40 30 20 10 0

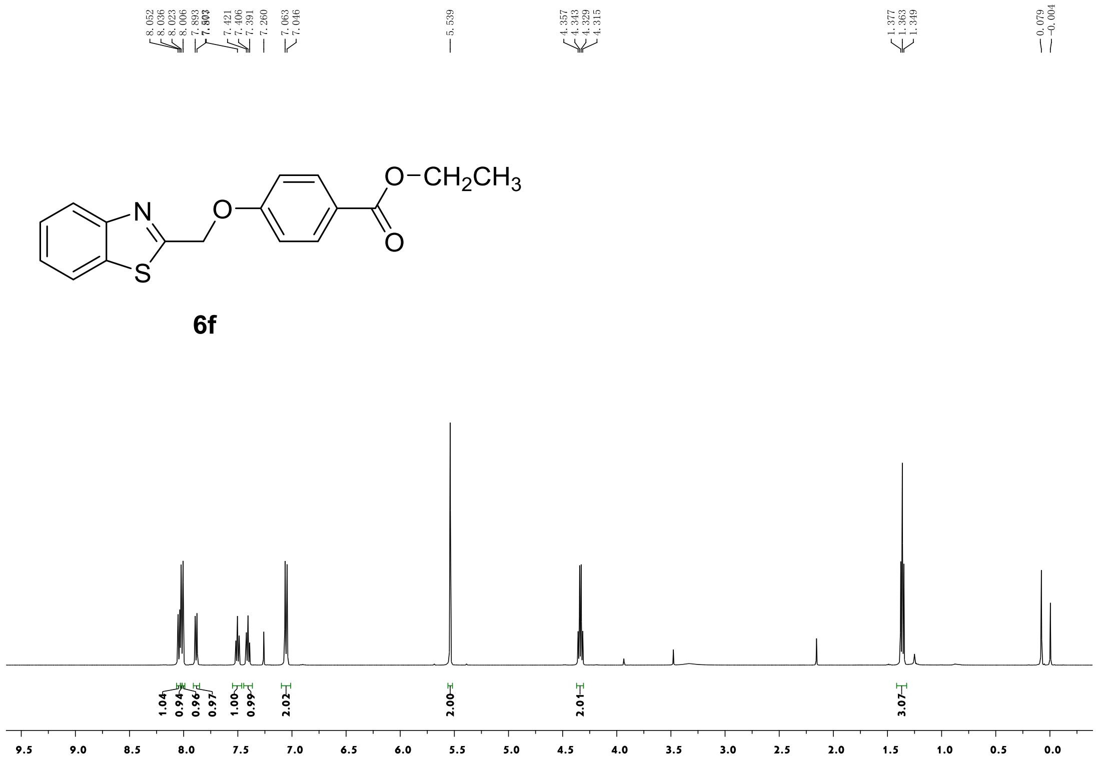
8.052
8.036
8.023
8.006
7.893
7.421
7.406
7.391
7.260
7.063
7.046
5.539
4.357
4.343
4.329
4.315
1.377
1.363
1.349
0.079
−0.004
O−CH2CH3
N
O
S
O
6f
1.04
0.94
0.96
0.97
1.00
0.99
2.02
2.00
2.01
3.07
9.5 9.0 8.5 8.0 7.5 7.0 6.5 6.0 5.5 5.0 4.5 4.0 3.5 3.0 2.5 2.0 1.5 1.0 0.5 0.0

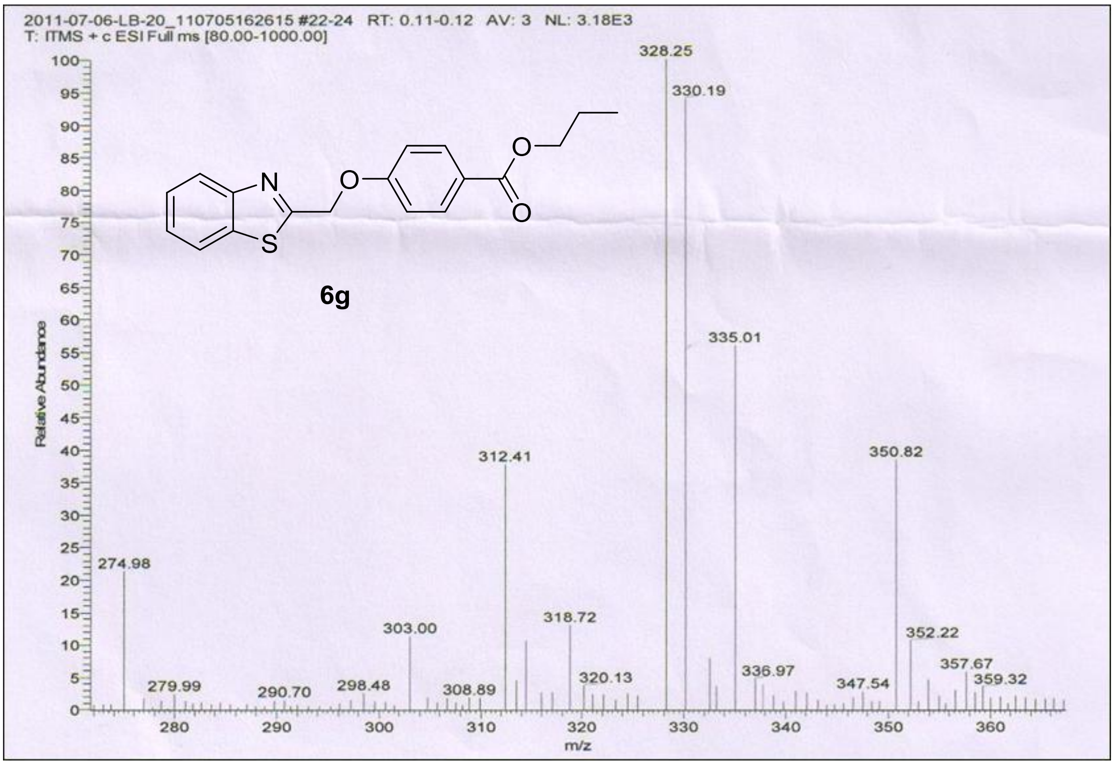
2011-07-06-LB-20_110705162615 #22-24 RT: 0.11-0.12 AV: 3 NL: 3.18E3
T: ITMS + c ESI Full ms [80.00-1000.00]
Relative Abundance
m/z
328.25
330.19
335.01
312.41
350.82
274.98
303.00
318.72
352.22
357.67
359.32
320.13
336.97
347.54
279.99
290.70
298.48
308.89
6g

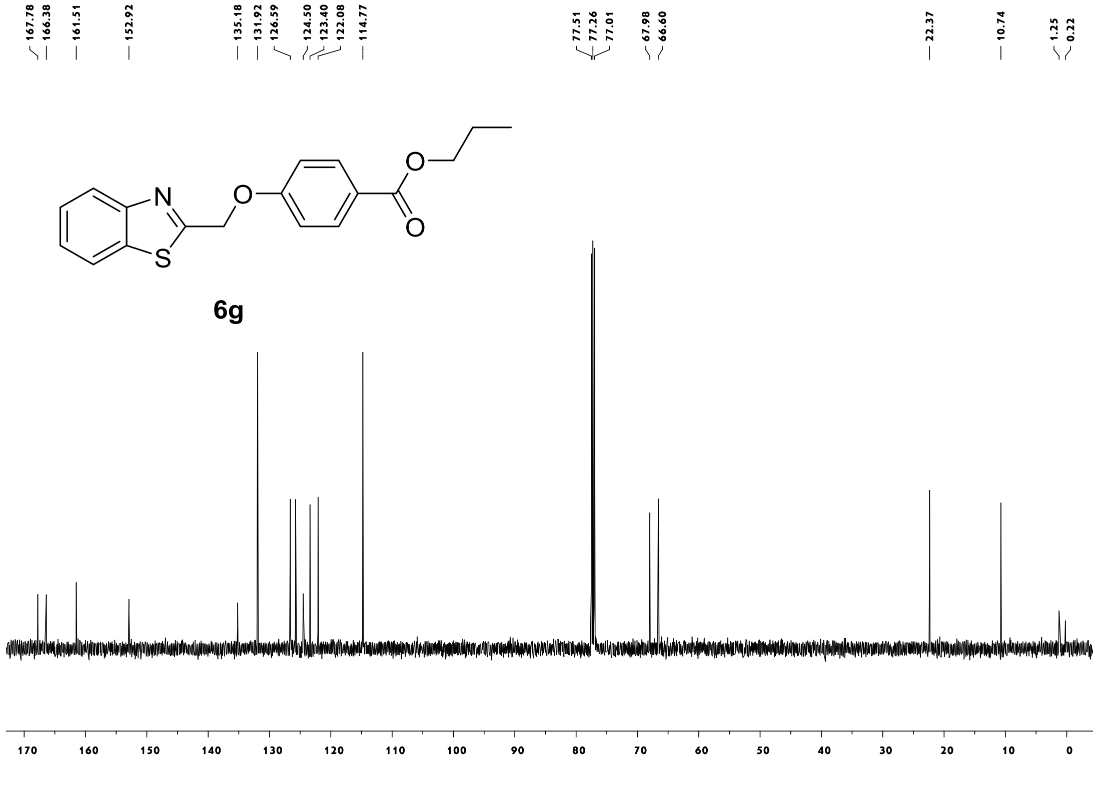
167.78
166.38
161.51
152.92
135.18
131.92
126.59
124.50
123.40
122.08
114.77
77.51
77.26
77.01
67.98
66.60
22.37
10.74
1.25
0.22
6g

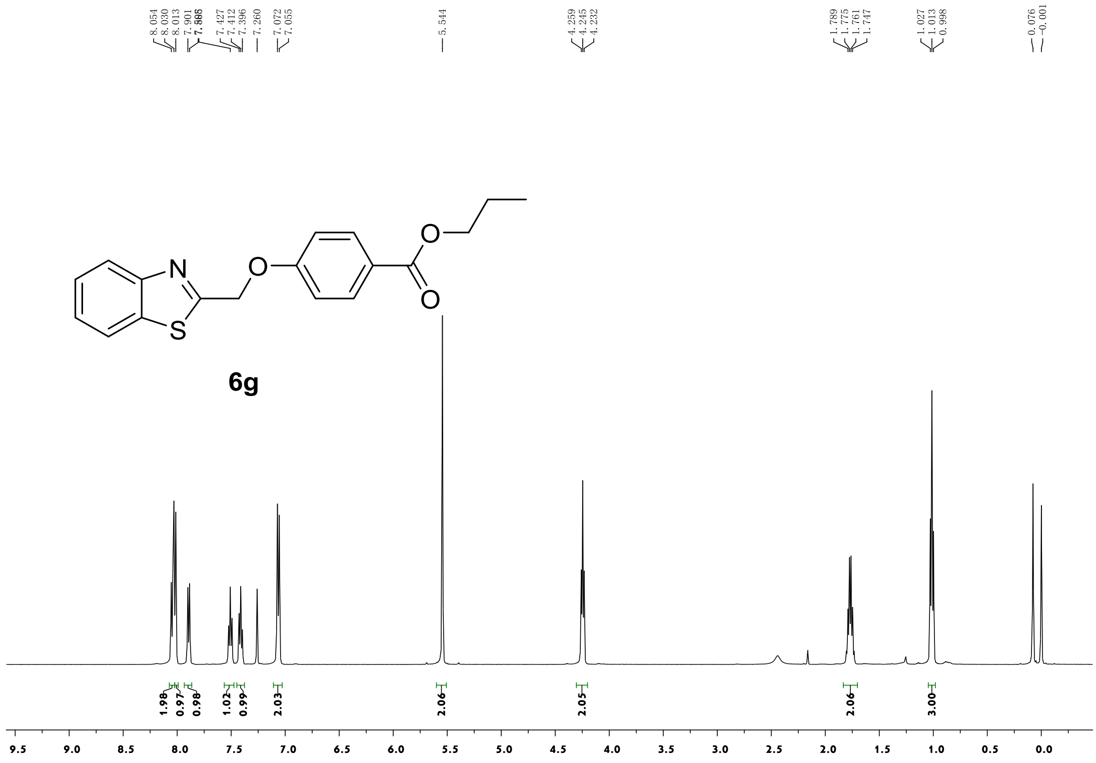

N
O
O
O
S
6g
1.98
0.97
0.98
1.02
0.99
2.03
2.06
2.05
2.06
3.00
9.5 9.0 8.5 8.0 7.5 7.0 6.5 6.0 5.5 5.0 4.5 4.0 3.5 3.0 2.5 2.0 1.5 1.0 0.5 0.0

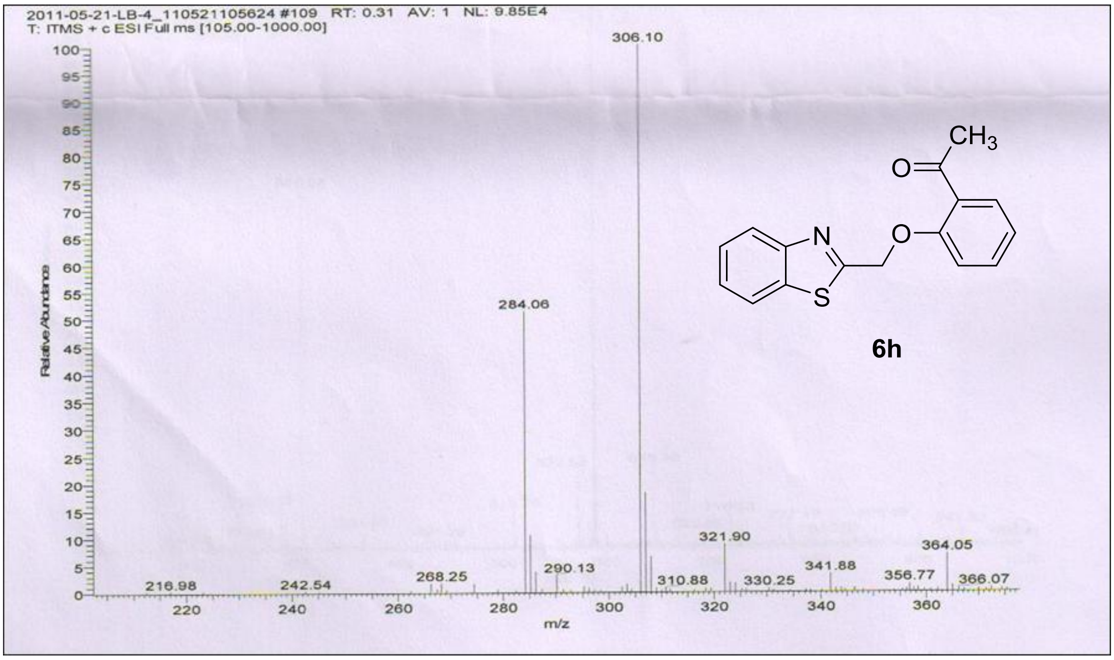

2011-05-21-LB-4_110521105624 #109 RT: 0.31 AV: 1 NL: 9.85E4
T: ITMS + c ESI Full ms [105.00-1000.00]
306.10
284.06
321.90
364.05
290.13
268.25
341.88
356.77
366.07
310.88
330.25
216.98
242.54
Relative Abundance
m/z
CH3
O
N
O
S
6h

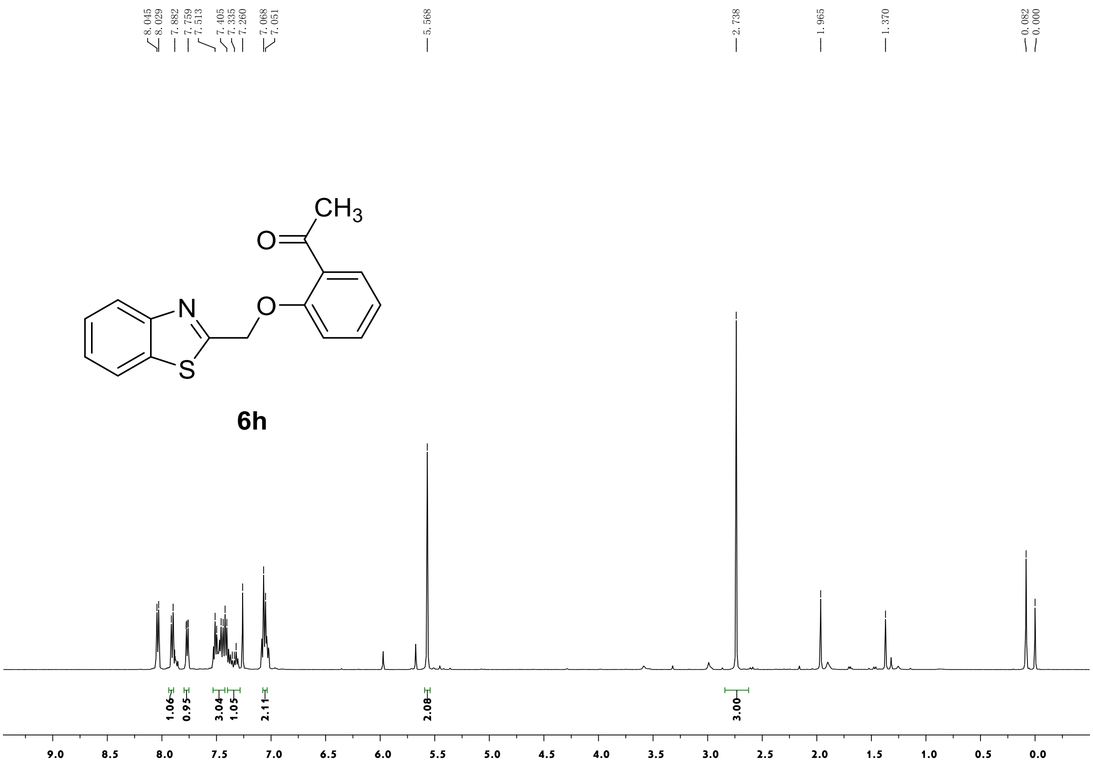
8.045
8.029
7.882
7.759
7.513
7.405
7.335
7.260
7.068
7.051
5.568
2.738
1.965
1.370
0.082
0.000
CH3
O
N
O
S
6h
1.06
0.95
3.04
1.05
2.11
2.08
3.00
9.0
8.5
8.0
7.5
7.0
6.5
6.0
5.5
5.0
4.5
4.0
3.5
3.0
2.5
2.0
1.5
1.0
0.5
0.0

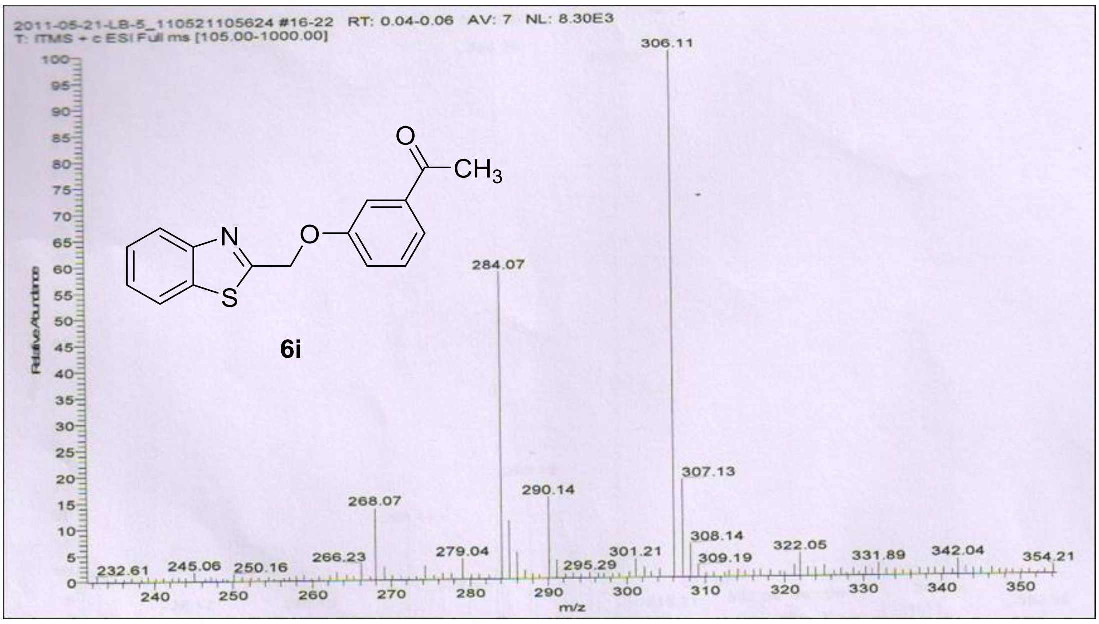
2011-05-21-LB-5_110521105624 #16-22 RT: 0.04-0.06 AV: 7 NL: 8.30E3
T: ITMS + c ESI Full ms [105.00-1000.00]
Relative Abundance
m/z
O
CH3
N
O
S
6i
306.11
284.07
307.13
290.14
268.07
308.14
322.05
279.04
301.21
331.89
342.04
354.21
232.61
245.06
250.16
266.23
295.29
309.19

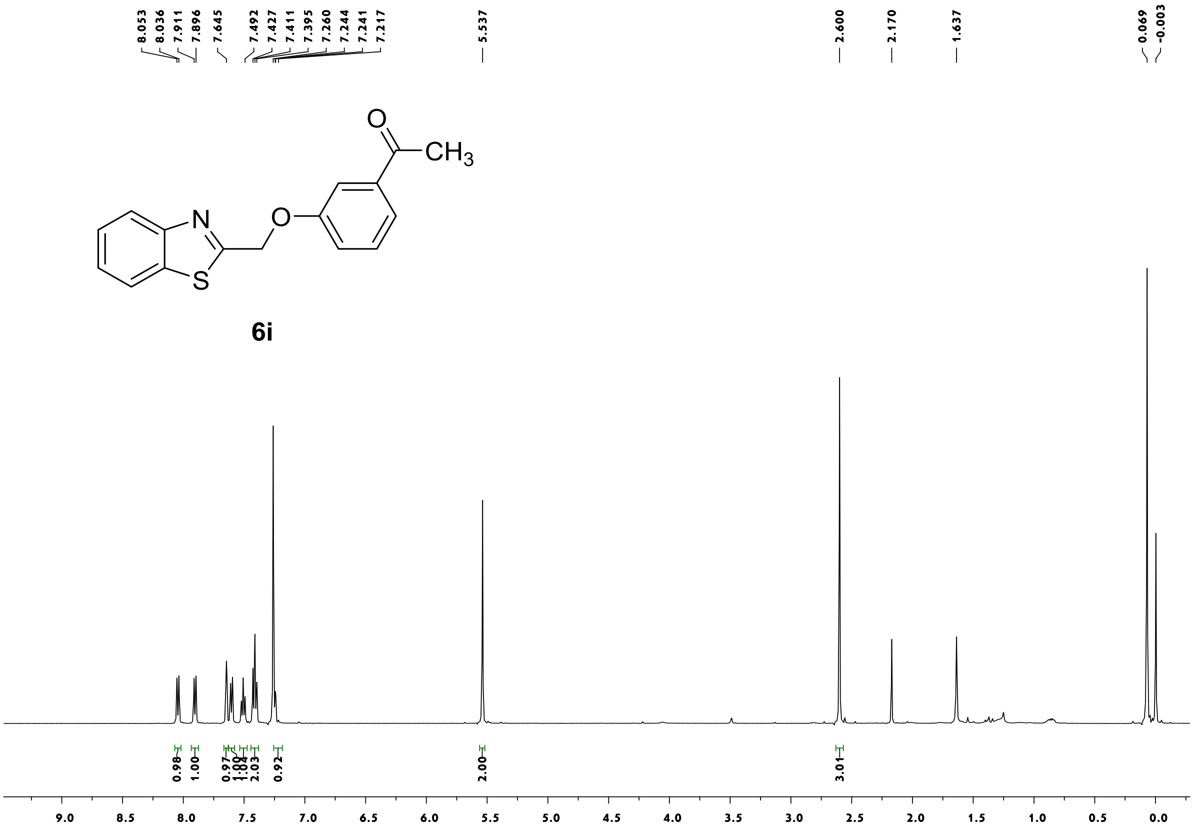

8.053 8.036 7.911 7.896 7.645 7.492 7.427 7.411 7.395 7.260 7.244 7.241 7.217
5.537
2.600
2.170
1.637
0.069 -0.003
O
CH3
N
O
S
0.98 1.00 0.97 1.02 1.00 2.03 0.92
2.00
3.01
9.0 8.5 8.0 7.5 7.0 6.5 6.0 5.5 5.0 4.5 4.0 3.5 3.0 2.5 2.0 1.5 1.0 0.5 0.0

6i

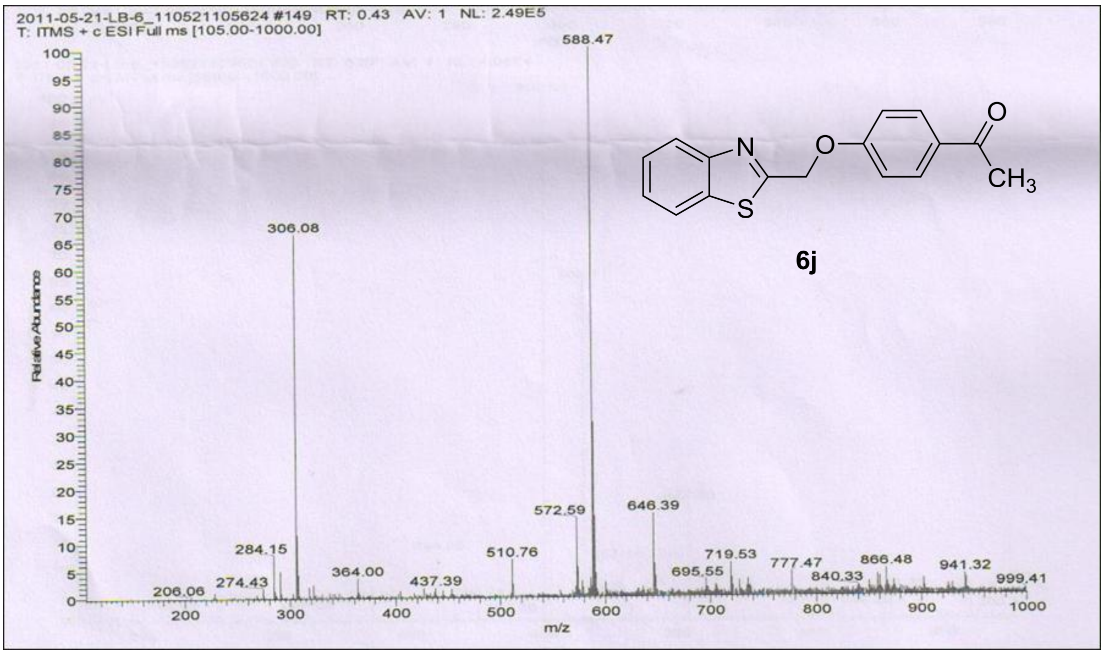

2011-05-21-LB-6_110521105624 #149 RT: 0.43 AV: 1 NL: 2.49E5
T: ITMS + c ESI Full ms [105.00-1000.00]
Relative Abundance
588.47
306.08
572.59
646.39
284.15
510.76
719.53
777.47
866.48
364.00
941.32
437.39
695.55
840.33
999.41
206.06
274.43
m/z
N
O
O
S
CH3

6j

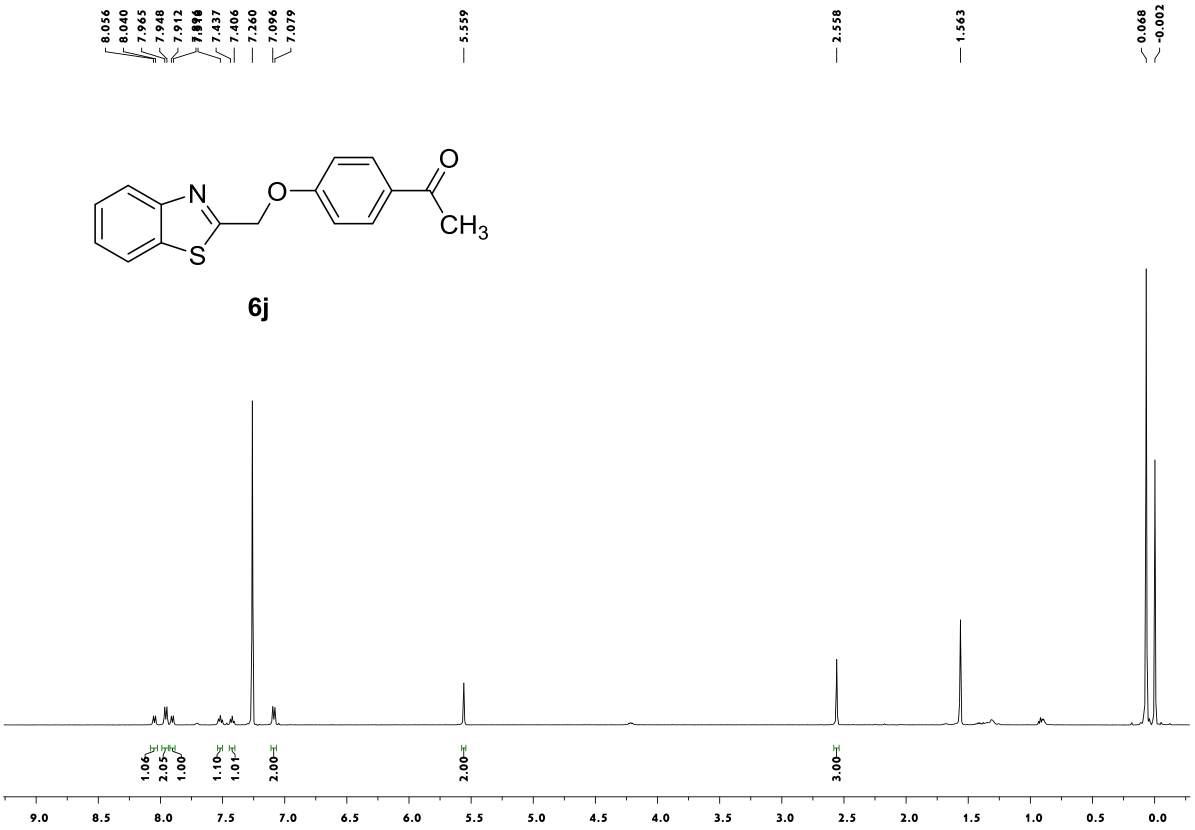

8.056
8.040
7.965
7.948
7.912
7.437
7.406
7.260
7.096
7.079
5.559
2.558
1.563
0.068
-0.002
N
O
O
S
CH3
6j
1.06
2.05
1.00
1.10
1.01
2.00
2.00
3.00
9.0 8.5 8.0 7.5 7.0 6.5 6.0 5.5 5.0 4.5 4.0 3.5 3.0 2.5 2.0 1.5 1.0 0.5 0.0

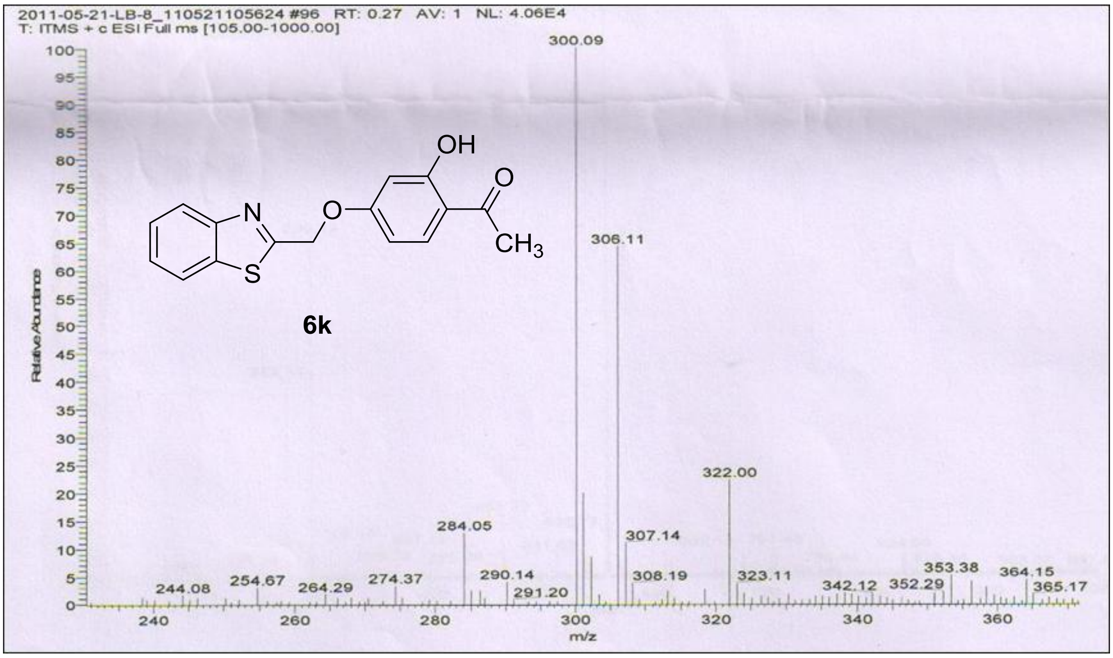

2011-05-21-LB-8_110521105624 #96 RT: 0.27 AV: 1 NL: 4.06E4
T: ITMS + c ESI Full ms [105.00-1000.00]
Relative Abundance
m/z
300.09
306.11
322.00
284.05
307.14
308.19
290.14
291.20
323.11
244.08
254.67
264.29
274.37
342.12
352.29
353.38
364.15
365.17
OH
O
N
O
S
CH3
6k

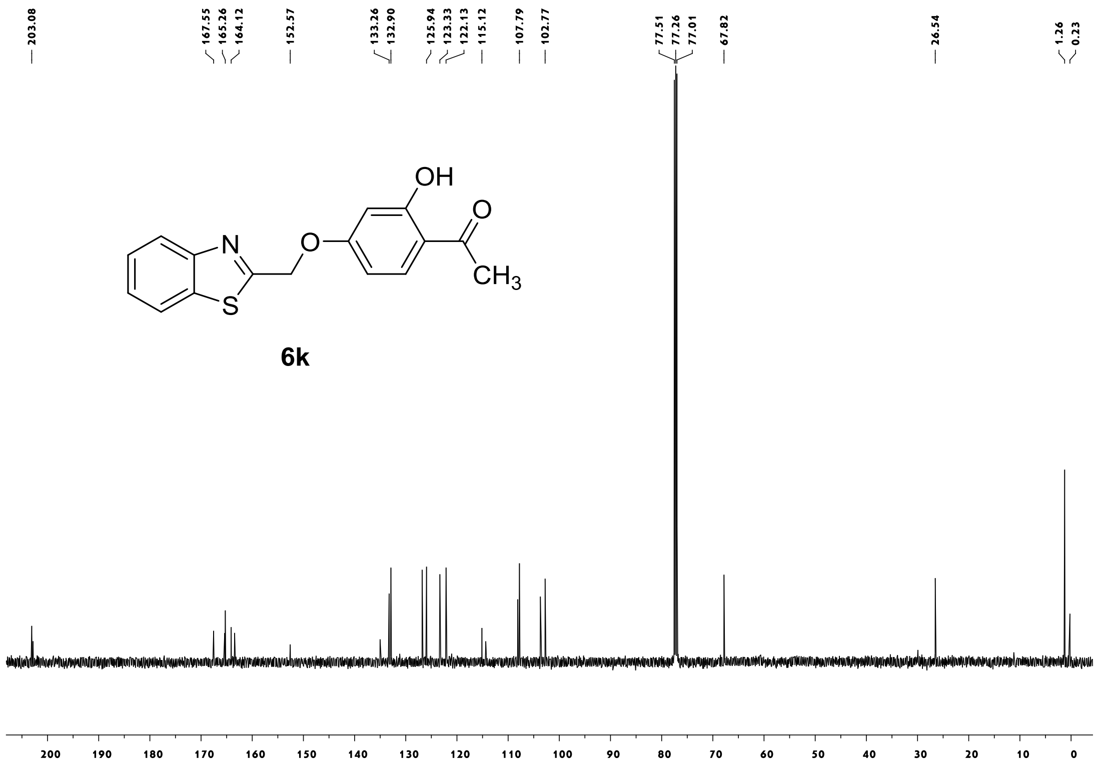
203.08
167.55
165.26
164.12
152.57
133.26
132.90
125.94
123.33
122.13
115.12
107.79
102.77
77.51
77.26
77.01
67.82
26.54
1.26
0.23
OH
O
N
S
CH3
6k
200 190 180 170 160 150 140 130 120 110 100 90 80 70 60 50 40 30 20 10 0

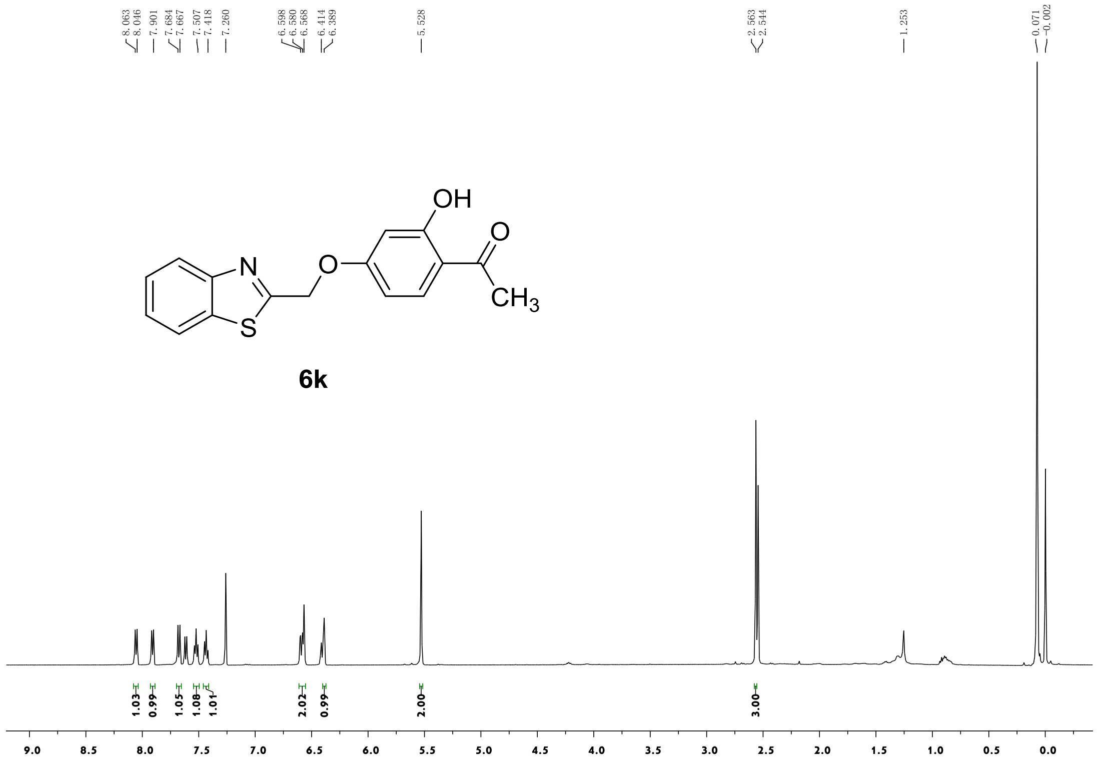
8.063
8.046
7.901
7.684
7.667
7.507
7.418
7.260
6.598
6.580
6.568
6.414
6.389
5.528
2.563
2.544
1.253
0.071
-0.002
OH
O
N
S
CH3
6k
1.03
0.99
1.05
1.08
1.01
2.02
0.99
2.00
3.00
9.0 8.5 8.0 7.5 7.0 6.5 6.0 5.5 5.0 4.5 4.0 3.5 3.0 2.5 2.0 1.5 1.0 0.5 0.0

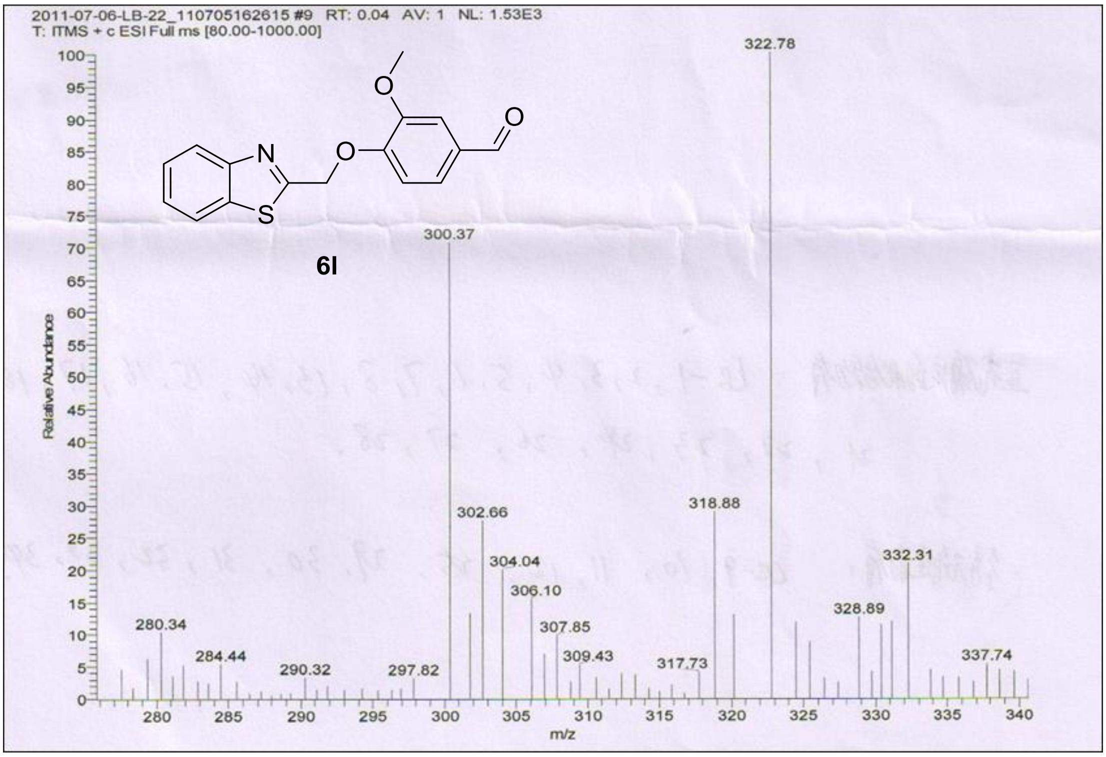
2011-07-06-LB-22_110705162615 #9 RT: 0.04 AV: 1 NL: 1.53E3
T: ITMS + c ESI Full ms [80.00-1000.00]
6l
322.78
300.37
302.66
304.04
306.10
307.85
309.43
317.73
318.88
332.31
328.89
337.74
280.34
284.44
290.32
297.82
Relative Abundance
m/z

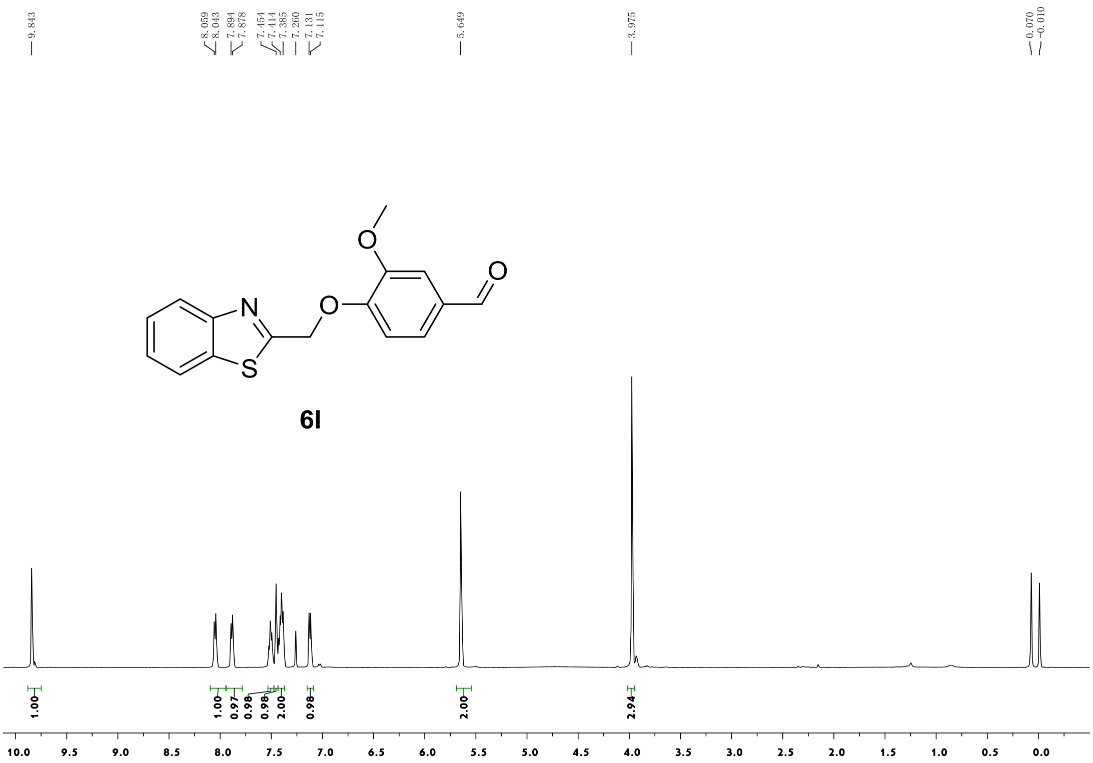
9.843
8.059
8.043
7.894
7.878
7.454
7.414
7.385
7.260
7.131
7.115
5.649
3.975
0.070
-0.010
6l
1.00
1.00
0.97
0.98
0.98
2.00
0.98
2.00
2.94

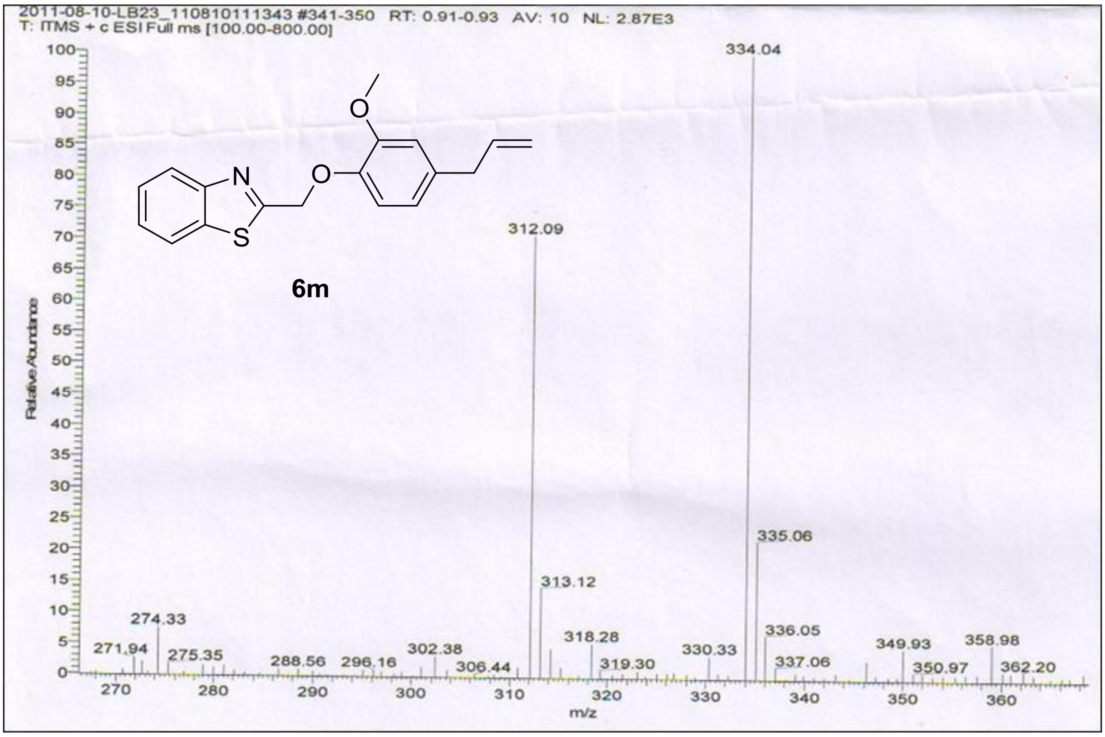
2011-08-10-LB23_110810111343 #341-350 RT: 0.91-0.93 AV: 10 NL: 2.87E3
T: ITMS + c ESI Full ms [100.00-800.00]
Relative Abundance
m/z
334.04
312.09
335.06
313.12
274.33
271.94
275.35
288.56
296.16
302.38
306.44
318.28
319.30
330.33
336.05
337.06
349.93
350.97
358.98
362.20
N
S
O
6m

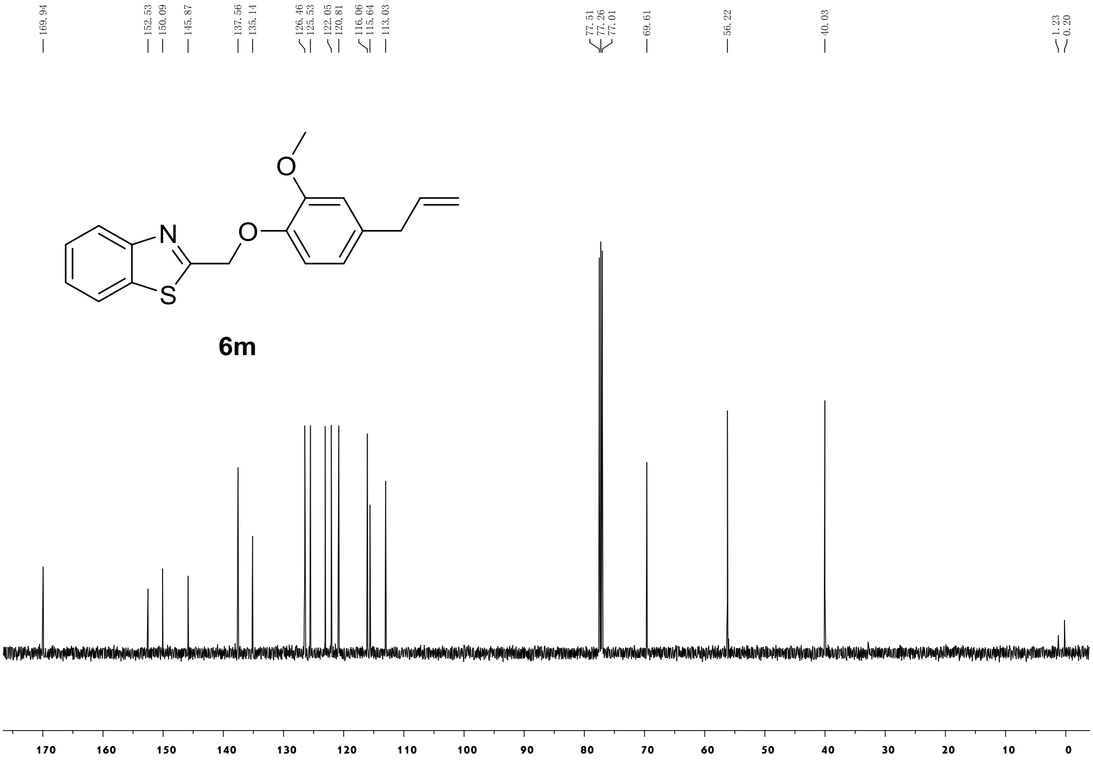
169.94
152.53
150.09
145.87
137.56
135.14
126.46
125.53
122.05
120.81
116.06
115.64
113.03
77.51
77.26
77.01
69.61
56.22
40.03
1.23
0.20
N
S
O
6m

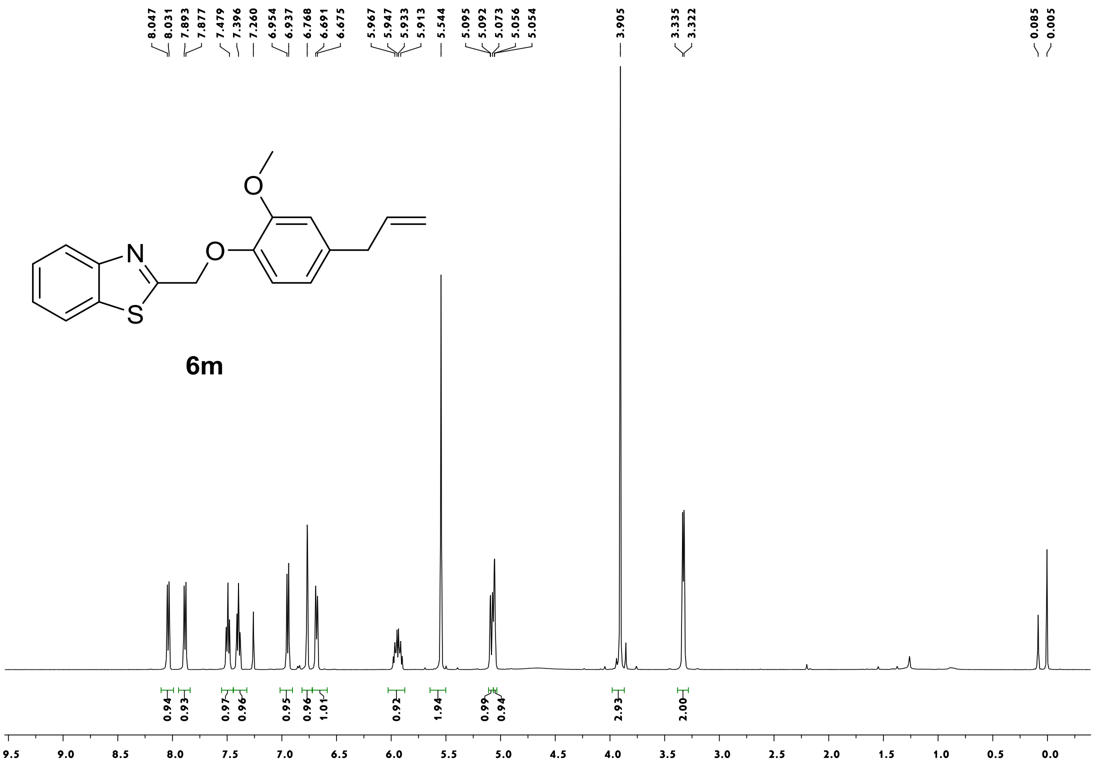
8.047
8.031
7.893
7.877
7.479
7.396
7.260
6.954
6.937
6.768
6.691
6.675
5.967
5.947
5.933
5.913
5.544
5.095
5.092
5.073
5.056
5.054
3.905
3.335
3.322
0.085
0.005
N
O
O
S
6m
0.94
0.93
0.97
0.96
0.95
0.96
1.01
0.92
1.94
0.99
0.94
2.93
2.00
9.5
9.0
8.5
8.0
7.5
7.0
6.5
6.0
5.5
5.0
4.5
4.0
3.5
3.0
2.5
2.0
1.5
1.0
0.5
0.0
